# Condensate-driven triglyceride reduction links α-synuclein to mitochondrial dysfunction

**DOI:** 10.1038/s44319-026-00954-7

**Published:** 2026-09-29

**Authors:** Tao Zhang, Alejandro Herron-Bedoya, María Eugenia Goya, Jorien C van der Weerd, Dikaia Tsagkari, Suzanne Couzijn, Lale Güngördü, Anna P Ainslie, Renée I Seinstra, M Rebecca Heiner Fokkema, Ming Chang, Nektarios Tavernarakis, Folkert Kuipers, Ellen A A Nollen

**Affiliations:** 1 European Research Institute for the Biology of Ageing, University of Groningen, University Medical Center Groningen, Groningen, The Netherlands; 2 College of Food Science and Technology, Huazhong Agricultural University, Wuhan, China; 3 Department of Laboratory Medicine, University Medical Center Groningen, University of Groningen, Groningen, The Netherlands; 4 Institute of Molecular Biology and Biotechnology, Foundation for Research and Technology-Hellas, Heraklion, Greece; 5 Division of Basic Sciences, School of Medicine, University of Crete, Heraklion, Greece; 6 State Key Laboratory of Food Science and Technology, School of Food Science and Technology, Jiangnan University, Wuxi, China

**Keywords:** Molecular Biology of Disease, Neuroscience

## Abstract

α-Synuclein (αSyn) inclusions characterize multiple age-related neurodegenerative diseases, including Parkinson’s disease (PD). While interactions between αSyn and lipids are known to contribute to αSyn pathobiology, the precise cellular mechanisms linking lipids to αSyn toxicity have yet to be elucidated. Through lipidomic profiling of *Caenorhabditis elegans*, we find that αSyn progressively alters lipid metabolism in aging worms. αSyn reduces the overall content of triacylglycerols (TAG) and disrupts the structure of lipid droplets (LD) and mitochondria. These pathological changes depend on αSyn’s properties to bind lipid and to condensate into inclusions. Apart from lowering TAG levels, αSyn proportionally increases long-chain unsaturated fatty acids (LCUFAs). Consequently, genetic inhibition of LCUFA biosynthesis alleviates αSyn-induced loss of *C. elegans* motility. Supplementing Medium-Chain Triglyceride (MCT) on the other hand also improves αSyn-associated toxicity phenotypes. These results link αSyn lipid binding and condensation to impaired TAG metabolism, which drives cellular toxicity. Combined with observed lower plasma TAGs in Parkinson cohorts, our findings reveal contributions of TAG remodelling to αSyn toxicity and point at MCT-supplementation as a mechanism-based therapeutic opportunity in age-related synucleinopathies.

## Synopsis

αSyn reduces and remodels triacylglycerol content and disrupts lipid droplet and mitochondrial structure in *C. elegans* in a lipid-binding and condensation-dependent manner. Blocking long-chain unsaturated fatty acid (LCUFA) biosynthesis or increasing medium-chain triglycerides (MCT) rescues αSyn-induced motor impairment.

- αSyn reduces overall TAG content and disrupts lipid droplet and mitochondrial structure in *C. elegans* in a lipid-binding and condensation-dependent manner.
- αSyn proportionally elevates long-chain unsaturated fatty acids (LCUFAs), and blocking LCUFA biosynthesis genetically rescues αSyn-induced motility defects.
- Medium-chain triglyceride (MCT) supplementation rescues αSyn-induced motor impairment.

## Abstract (web summary)

αSyn reduces and remodels triacylglycerol content and disrupts lipid droplet and mitochondrial structure in *C. elegans* in a lipid-binding and condensation-dependent manner. Blocking long-chain unsaturated fatty acid (LCUFA) biosynthesis or increasing medium-chain triglycerides (MCT) rescues αSyn-induced motor impairment.

## Graphical abstract

**Figure:**
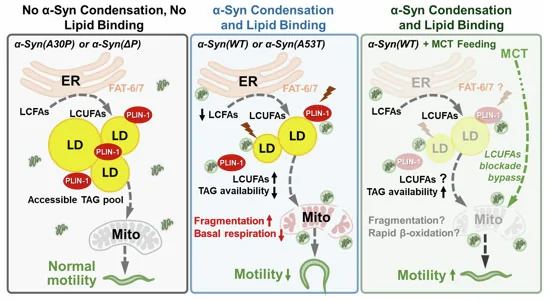

## Introduction

α-Synuclein (αSyn) is an intrinsically disordered protein implicated in the pathogenesis of a number of age-related neurodegenerative diseases, including Parkinson’s Disease (PD), Multiple System Atrophy (MSA), and Dementia with Lewy Bodies (DLB) (Spillantini et al, 1997). Abnormal αSyn conformations and trafficking defects promote formation of proteinaceous inclusions such as Lewy bodies, which disrupt cellular function and eventually lead to disease symptoms (Fanning et al, 2021; Lee and Trojanowski, 2006). The N-terminal region of αSyn contains seven conserved KTKEGV motif repeats that mediate binding to negatively charged membrane lipids. Recent studies suggest that αSyn-induced toxicity arises from its dual role in disrupting lipid raft architecture and interfering with metabolic pathways (Imberdis et al, 2019; Tsagkari et al, 2025; Zhao et al, 2024). Specifically, αSyn penetrates lipid rafts via hydrophobic interactions with the membrane lipids, ultimately forming pores that disrupt the integrity of the membrane (Fusco et al, 2017). This lipid-based mechanism is consistent with disease-associated mutations in genes such as glucocerebrosidase (GBA) (Mazzulli et al, 2011), sterol regulatory element-binding protein 1 (SREBP-1) (Do et al, 2011), and diacylglycerol kinase theta (DGKQ) (Nalls et al, 2014). However, while in vitro interactions between lipids and αSyn have been characterized (Girard et al, 2021; Imberdis et al, 2019), downstream impacts on lipid metabolism and resulting consequences on αSyn toxicity remain poorly understood.

Multiple lines of evidence suggest that αSyn toxicity is associated with disruptions in fatty acid saturation and overall lipid composition, both of which are essential for maintaining cellular homeostasis. These disruptions involve changes in monounsaturated fatty acids (MUFAs) (Kula and Kuter, 2024), sphingolipids (Goya et al, 2020), and phospholipid species (Wang et al, 2014). Given the diversity of lipids and their alterations following αSyn condensation, it is clear that disturbed lipid homeostasis is closely associated with αSyn toxicity during development and progression of synucleinopathies. However, whether specific disruptions in lipid metabolism result from the loss of αSyn’s physiological function or from its toxic gain-of-function remains an unresolved question.

Previously, we developed a transgenic αSyn model in *Caenorhabditis elegans* (*C. elegans*) in which the human αSyn gene, fused to the Yellow Fluorescent Protein (YFP) gene, is expressed in the body-wall muscle cells (van Ham et al, 2008). This model enables real-time visualization of αSyn condensation, aggregation and age-dependent motility decline, recapitulating aspects of αSyn pathology observed in patients (Hardenberg et al, 2021; Perni et al, 2021; van Ham et al, 2008). In our previous genetic screen in this model, we identified several modifiers of αSyn toxicity and candidates of interest, in particular genes linked to lipid metabolism (Perni et al, 2017; van der Goot et al, 2012). Because central features of lipid metabolism pathways are conserved between humans and *C. elegans*, including shared homologs of human lipid-associated genes (Watts and Ristow, 2017), *C. elegans* is a valuable model for identifying the origins of lipid dysregulation and its contribution to age-related diseases (Kaletta and Hengartner, 2006; Srinivasan, 2015).

Here, by performing a lipid metabolism screen in *C. elegans* expressing αSyn-YFP, we identified that αSyn condensation is associated with lower triacylglycerol (TAG) content and an increased proportion of long-chain unsaturated fatty acids (LCUFAs) in the worm. This phenotype is associated with changed properties of αSyn condensates and formation of inclusions that disrupt dynamics of lipid droplet (LD) formation and alter localization of the LD structural protein PLIN-1. Notably, we found that bypassing defects in TAG metabolism by medium-chain triglyceride (MCT) feeding rescues mitochondrial function and motility toxicity. Taken together, our findings provide insights into cellular mechanisms promoting αSyn toxicity and propose potential therapeutic strategies for alleviating synuclein pathology.

## Results

### αSyn condensation causes changes in lipidome and lowers triacylglycerol levels in *C. elegans*

**Figure 1:**
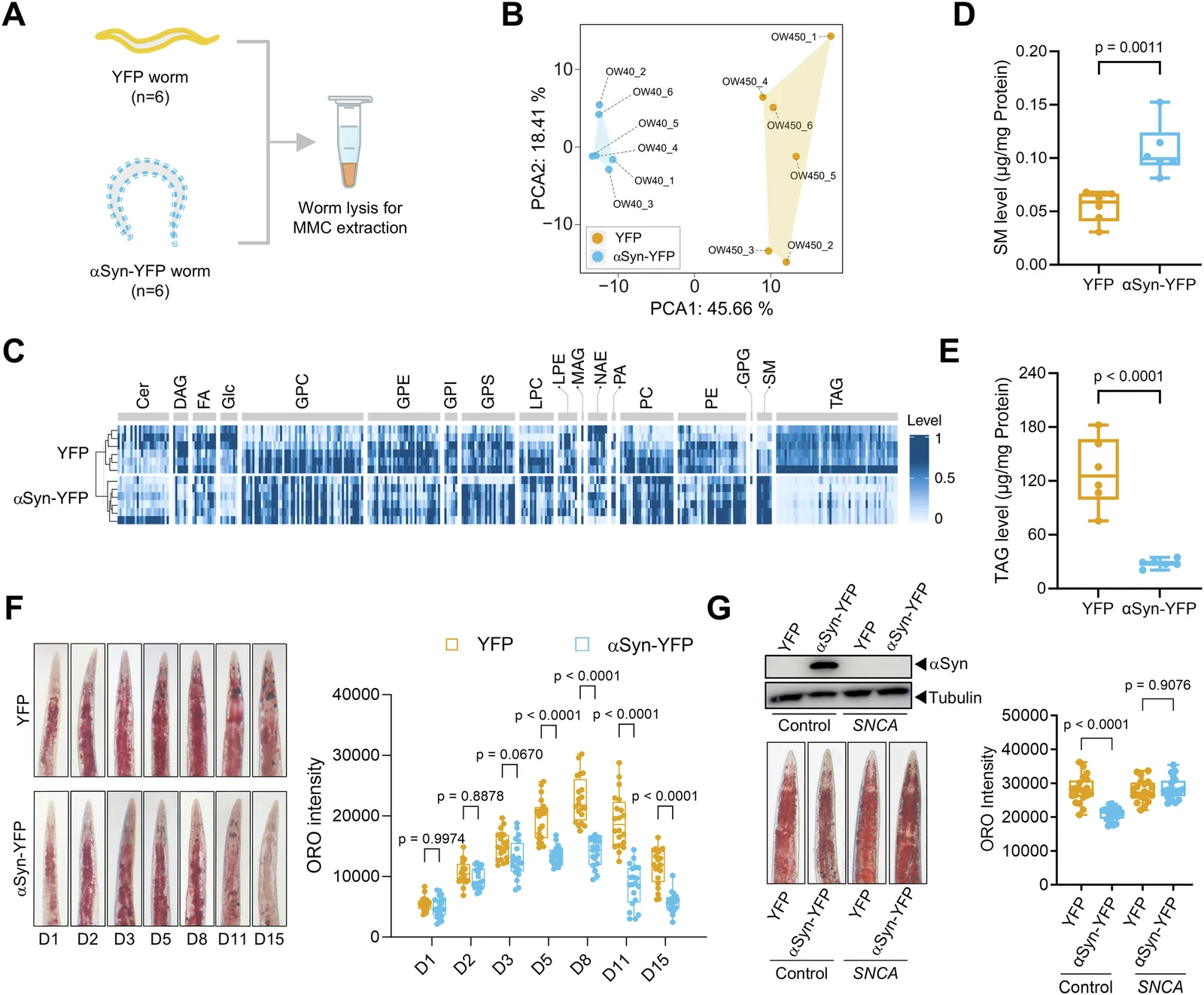
αSyn alters the lipid profile and reduces total triacylglycerol (TAG) content in *C. elegans*. (**A**) Schematic of the sample preparation workflow for untargeted lipidomics. Lipids were extracted using the MMC method from whole-worm lysates of YFP and αSyn-YFP worms at day 11 of adulthood. Each lipidomics sample contained ~3000 worms collected from six replicate plates. (**B**) Principal component analysis (PCA) of lipidomics data showing distinct clustering of αSyn-YFP and YFP worms (*n* = 6 replicates). (**C**) Heatmap showing normalized lipid abundances across lipid classes, with reduced TAG abundance in αSyn-YFP worms compared with YFP controls (*n* = 6). Data were normalized using min–max scaling followed by log transformation. (**D**) Sphingomyelin (SM) abundance in αSyn-YFP and YFP worms. Box plots show the median, IQR, and min–max whiskers (unpaired *t* test, *n* = 6). (**E**) TAG abundance in αSyn-YFP and YFP worms. Box plots show the median, IQR, and min–max whiskers (unpaired *t* test, *n* = 6). (**F**) Oil Red O staining shows decreased total TAG level in αSyn-YFP worms from day 5 of adulthood compared to YFP controls. Box plots show the median, IQR, and min–max whiskers (*n* = 20 animals; unpaired *t* test). (**G**) Knockdown of αSyn (*SNCA*) rescues TAG levels in αSyn-YFP worms at day 5 of adulthood. Box plots show the median, IQR, and min–max whiskers (*n* = 20 animals; one-way ANOVA). (**F**, **G**) One representative ORO staining worm is shown in each condition. Cer ceramide, DAG diacylglycerol, FA fatty acid, Glc glucosylceramide, GPC glycerophosphocholine, GPE glycerophosphoethanolamine, GPI glycosylphosphatidylinositol, GPS glycerophosphoserine, LPC lysophosphatidylcholine, LPE lysophosphatidylethanolamine, MAG monoacylglycerol, NAE *N*-acylethanolamine, PA phosphatidic acid, PC phosphatidylcholine, PE phosphatidylethanolamine, GPG phosphatidylglycerol, SM sphingomyelin, TAG triacylglycerol. Source data are available online for this figure.

In previous work, we identified that αSyn initiates self-assembly of amyloid-like condensates in *C. elegans* on day 11 of adulthood (a stage of advanced aging) (Hardenberg et al, 2021). Here, we conducted an untargeted lipidomic screen on day 11 αSyn-YFP expressing worms to investigate how αSyn-dependent activity influences lipid metabolism (Fig. 1A). To ensure that we acquired a broad representation of the lipidome, and in particular of moderately and highly apolar lipids, we used the MMC (MeOH/MTBE/CHCl_3_) approach (Gil et al, 2018). When we applied unsupervised principal component analysis (PCA), we identified distinct clustering between the lipid profiles of the αSyn-YFP and YFP strains (Fig. 1B), indicating significant lipidome differences. In all, we quantified a total of 18 different lipid classes (Fig. 1C). Consistent with previous related work, we observed an increase in SM levels in αSyn-YFP worms (Fig. 1D) (Signorelli et al, 2021). Intriguingly, our analyses revealed significant lower TAG abundance with a 72% reduction compared to the YFP control (*P* < 0.001) (Fig. 1E).

We next used Oil Red O (ORO) staining to validate the lower TAG level in a time-course analysis (days 1–15). While ORO intensity initially increased and then declined in both groups, staining intensity was markedly lower after day five in αSyn-YFP worms (Fig. 1F), consistent with our lipidomic analysis (Fig. 1C,E). Moreover, as no significant difference in overall ORO intensity was observed at the young adult stage (Fig. 1F), the decreased TAG levels are unlikely to be caused by developmental defects. Taken together, these results align with the age-dependent progression of αSyn toxicity that manifests in later stages of PD and related synucleinopathies.

**Figure EV1:**
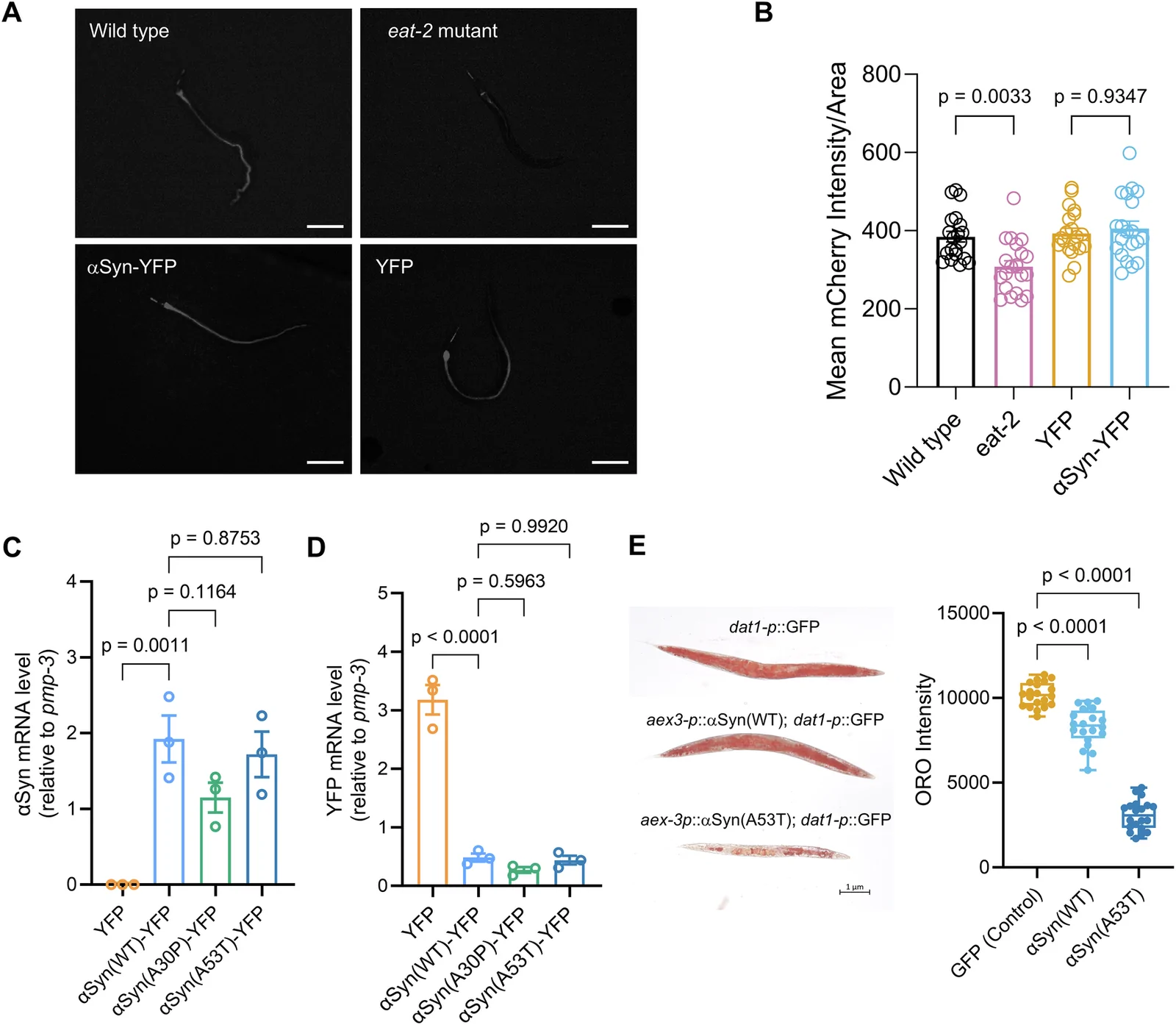
Comparable food intake and αSyn/YFP transcript levels across strains, with neuronal αSyn expression associated with reduced TAG content. (**A**) Representative images of intestinal fluorescence in αSyn-YFP and YFP control worms fed mCherry-expressing *E. coli* OP50. Scale bar, 20 μm. (**B**) Quantification of intestinal OP50::mCherry fluorescence intensity shows comparable bacterial intake between αSyn-YFP and YFP worms (*n* = 20 animals per group; one-way ANOVA, error bars represent s.e.m.). (**C**) αSyn mRNA levels were comparable among the indicated αSyn-expressing strains (one-way ANOVA, error bars represent s.e.m.). (**D**) YFP mRNA levels were likewise comparable among the indicated αSyn-expressing strains (one-way ANOVA, error bars represent s.e.m.). (**E**) Pan-neuronal expression of αSyn(WT) or αSyn(A53T), driven by the *dat-1* promoter, reduced TAG levels relative to *dat-1p*::GFP controls at day 5 of adulthood. Box plots show the median, IQR, and min–max whiskers (*n* = 20 animals; one-way ANOVA). (**A**, **E**) One representative image is shown in each condition. All experiments were performed in triplicate. Source data are available online for this figure.

To assess whether the TAG reduction was due to decreased food intake, we quantified intestinal fluorescence in worms fed mCherry-expressing *E. coli* OP50 (Fig. EV1A). As we did not detect any significant difference in fluorescence intensity between αSyn-YFP and YFP control worms (Fig. EV1B), we ruled out reduced food consumption as a contributing factor to the lower TAG levels. We also performed RNAi-mediated knockdown of the *SNCA* gene to investigate whether reduced αSyn expression directly drives the reduction in TAG levels (Fig. 1G). While the intensity of ORO staining in control αSyn RNAi worms was lower than in YFP RNAi worms, depletion of *SNCA* restored TAG levels to those of YFP worms (Fig. 1G). Together, these results demonstrate that αSyn causes lipidomic changes and lowers TAG levels in *C. elegans*.

### Reduction in TAG contents is associated with the formation of αSyn inclusions

**Figure 2:**
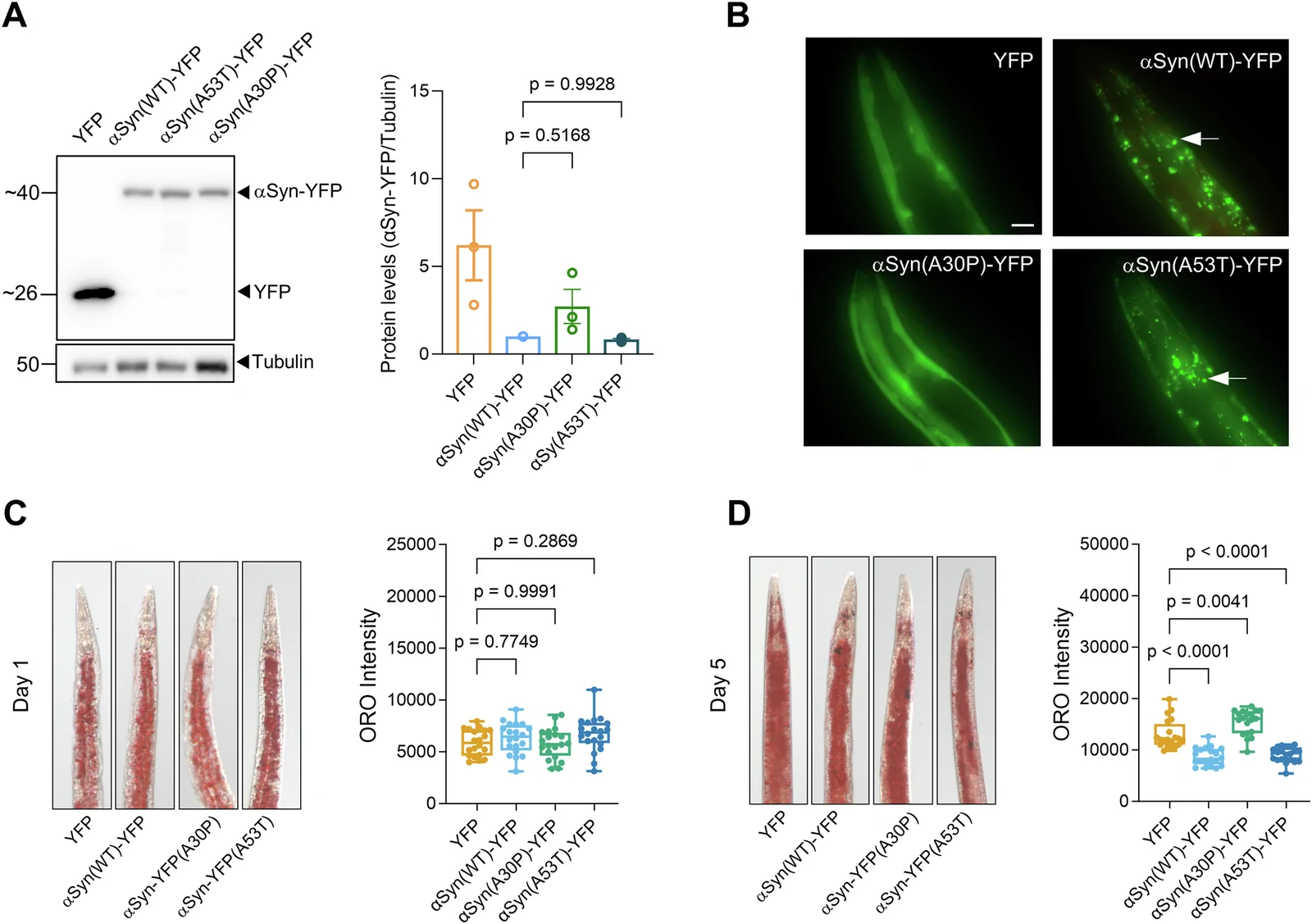
Reduced total TAG levels depend on αSyn inclusion-formation. (**A**) Western blot analysis shows comparable αSyn expression levels in αSyn(WT)-YFP, αSyn(A30P)-YFP, and αSyn(A53T)-YFP worms using an anti-GFP antibody. Tubulin was used as control (*n* = 3; one-way ANOVA, error bars represent s.e.m). (**B**) Representative fluorescence images showing αSyn inclusions (white arrow) in αSyn(WT)-YFP and αSyn(A53T)-YFP worms, but not in YFP or αSyn(A30P)-YFP worms. Scale bar: 20 µm. (**C**) ORO staining reveals no significant difference was observed at day 1 of adulthood. Box plots show the median, IQR, and min–max whiskers (*n* = 20 animals per group; one-way ANOVA). (**D**) ORO staining reveals significantly lower TAG levels in inclusion-forming αSyn(WT)-YFP and αSyn(A53T)-YFP worms compared to YFP and αSyn(A30P)-YFP controls at day 5 of adulthood. Box plots show the median, IQR, and min–max whiskers (*n* = 20 animals per group; one-way ANOVA). All experiments were performed in triplicate. Source data are available online for this figure.

We next asked whether the reduction in TAG is dependent on αSyn’s properties to form condensates and inclusions. To address this question, we assessed TAG levels across wild-type αSyn(WT) and strains expressing two pathogenic αSyn variants (A53T and A30P), which are associated with familial PD and related synucleinopathies. As shown in Fig. 2A, αSyn protein levels were comparable among all αSyn-expressing strains. Consistently, the transcript levels of both αSyn and YFP were not significantly different across these strains (Fig. EV1C,D). Despite similar αSyn expression levels, αSyn(WT)-YFP worms and αSyn(A53T)-YFP worms exhibit the stronger inclusion formation phenotype, while αSyn(A30P)-YFP worms display substantially attenuated inclusion formation (Fig. 2B), which is consistent with our previous findings (Perni et al, 2021). Importantly, TAG levels were significantly reduced in αSyn(WT)-YFP and αSyn(A53T)-YFP worms by day 5 (Fig. 2D), whereas no significant TAG reduction was observed in αSyn(A30P)-YFP worms at day 1 (Fig. 2C).

Pan-neuronal expression of αSyn(WT) or αSyn(A53T) was associated with reduced TAG at day 5 of adulthood (Fig. EV1E). These data support that TAG depletion is in part driven by αSyn’s ability to form condensates. In addition, and consistent with its reported stronger neurotoxicity (Perni et al, 2021), TAG reduction was more pronounced in neuronal αSyn(A53T)-expressing worms, supporting that the A53T mutation exerts a potent disruption on lipid homeostasis. These results suggest that αSyn-mediated TAG reduction is associated with the inclusion-forming capacity of αSyn and is unlikely to result from differences in transgene expression, supporting a fundamental disruption of TAG metabolism.

### αSyn reduces lipid droplet abundance and disrupts PLIN-1 localization in muscle

**Figure EV2:**
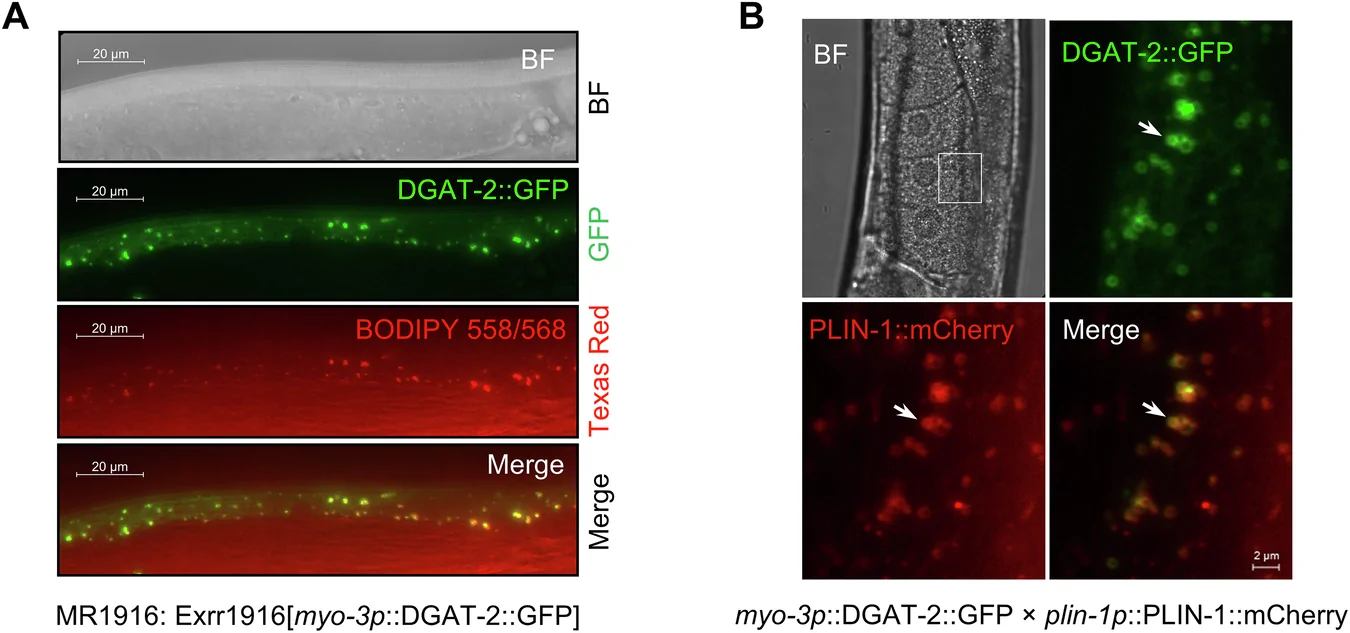
BODIPY staining identifies muscle LDs and confirms PLIN-1 localization to muscle-specific LDs. (**A**) Muscle-specific lipid droplets (LDs), visualized by BODIPY 558/568 C12 staining, colocalize with DGAT-2::GFP expressed under the *myo-3* promoter in strain MR1916, confirming the identity of BODIPY-positive structures as LDs. (**B**) Muscle-specific co-expression of DGAT-2::GFP (green) and PLIN-1::mCherry (red) demonstrates localization of PLIN-1 to muscle LDs. BF brightfield. Source data are available online for this figure.

**Figure 3:**
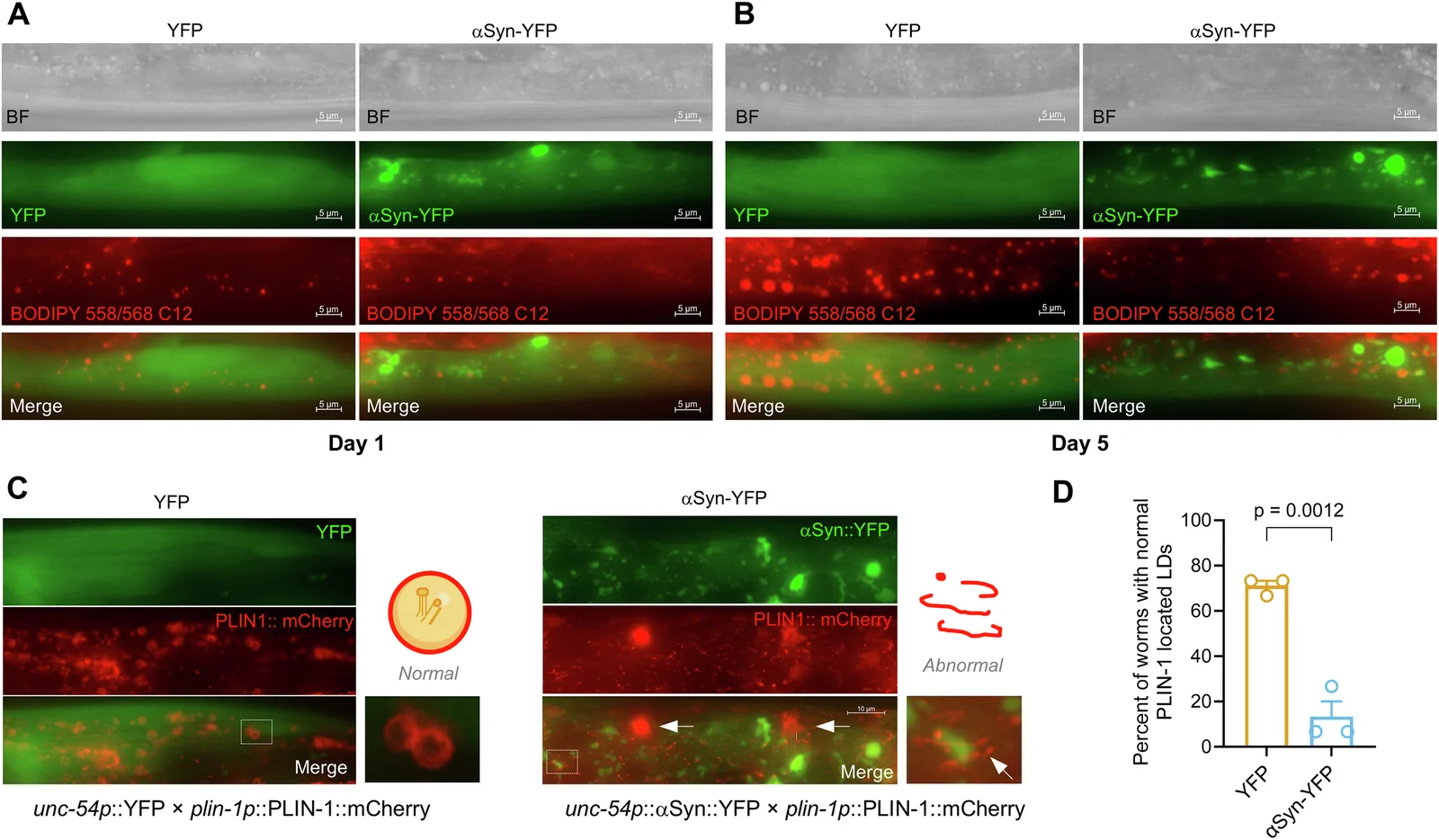
αSyn reduces lipid droplet (LD) abundance and disrupts PLIN-1 localization. (**A**) αSyn significantly reduces LD abundance in muscles compared to YFP controls at earlier day 1 of adulthood. Scale bar: 5 µm. One representative image is shown. (**B**) αSyn significantly reduces LD abundance in muscles compared to YFP controls at day 5 of adulthood. Scale bar: 5 µm. One representative image is shown. (**C**) Fluorescence reveals αSyn (green) induces abnormal PLIN-1 distributions including frilly morphologies (white arrow) at day 5 of adulthood. Insets show magnified views of disrupted PLIN-1 localization (white-dotted box regions) and the simplified diagram of disrupted PLIN-1 structure in αSyn-YFP worms. Scale bar: 10 μm. One representative image is shown. (**D**) αSyn inclusion formation decreases the percentage of worms with normal PLIN-1-coated LDs in muscle at day 5 of adulthood (Percentages calculated for three biological replicates; *n* = 15 animals per replicate; unpaired *t* test, error bars represent s.e.m). All experiments were performed in triplicate. Source data are available online for this figure.

ORO staining reveals overall TAG levels in the worms, and we therefore next questioned whether these are similarly reduced in body-wall muscle cells. TAG is primarily stored in lipid droplets (LDs), which can be visualized using the fluorescent fatty acid analogue BODIPY 558/568 C12. This probe is preferentially incorporated into TAG-rich LDs, allowing detection of LDs (Schmeisser et al, 2019). By feeding worms with BODIPY 558/568 C12, we confirmed LD labelling in muscle cells through co-localization with the LD-resident protein DGAT-2, tagged with GFP and expressed under the muscle-specific promoter (*myo-3*) (Fig. EV2A). In αSyn-expressing worms, we identified a striking reduction in muscle LDs at both day 1 and day 5 of adulthood (Fig. 3A,B), suggesting that αSyn-mediated TAG reduction occurs earlier at its primary site of expression.

**Figure EV3:**
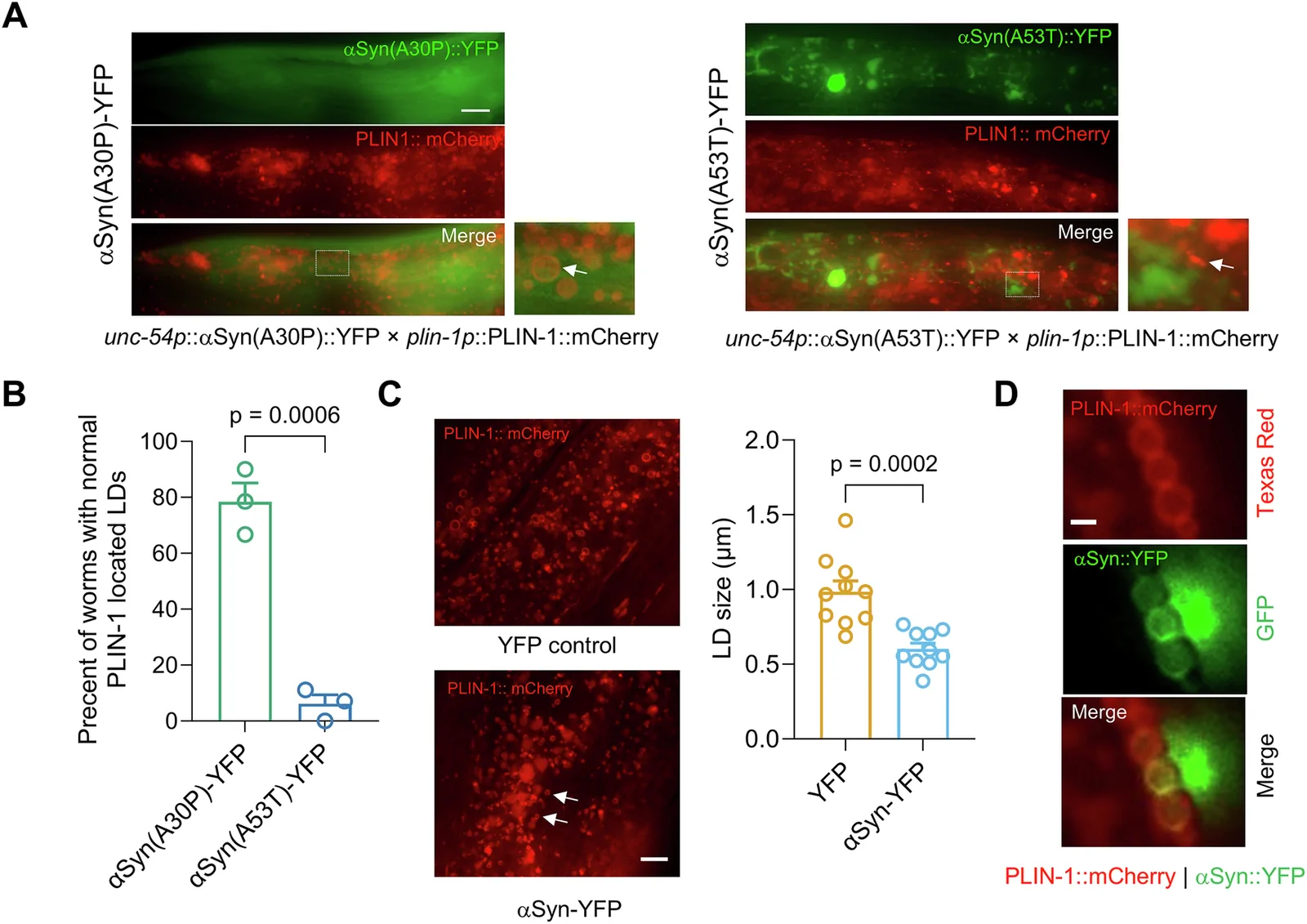
αSyn-YFP expression and condensation disrupts PLIN-1 localization and LD organization. (**A**) Compared with αSyn(A30P)-YFP, αSyn(A53T)-YFP expression is associated with abnormal PLIN-1 distribution and the appearance of filamentous PLIN-1 structures (white arrows). Insets show magnified views of the indicated regions (white dashed boxes). Scale bar, 10 μm. (**B**) αSyn(A53T) significantly reduces the proportion of worms displaying normal PLIN-1-coated lipid droplets (LDs) at day 5 of adulthood (percentages calculated for three biological replicates; *n* = 15 animals per replicate; unpaired *t* test, error bars represent s.e.m.). (**C**) αSyn-YFP worms exhibit significantly smaller LDs than YFP controls at day 11 of adulthood (*n* = 10 animals; unpaired *t* test, error bars represent s.e.m.). Scale bar, 5 μm. (**D**) Co-localization between αSyn::YFP and PLIN-1::mCherry is observed at day 1 of adulthood. Scale bar, 1 μm. Source data are available online for this figure.

We next examined whether αSyn condensation affects LD-associated proteins. We focused specifically on PLIN-1, a structural LD-coating protein that regulates lipolysis by controlling fatty acid release from LD-stored TAG for energy supply (Xie et al, 2019; Xu et al, 2012). We first confirmed expression of PLIN-1::mCherry in muscle by demonstrating its co-localization with DGAT-2::GFP under the muscle-specific promoter (Fig. EV2B). Next, we examined the changes in PLIN-1 co-localization. In αSyn-YFP worms, we confirmed that PLIN-1 was severely mislocalized at day 5 of adulthood (Fig. 3C), with a lower percentage of worms displaying normal PLIN-1-coated LDs compared to YFP controls (Fig. 3D). αSyn induces abnormal PLIN-1 distributions as shown in the magnified insets and the simplified diagram of PLIN-1 structure. The abnormal distribution was also more prominent in condensation-prone αSyn(A53T)-YFP worms relative to non-condensating αSyn(A30P)-YFP worms (Fig. EV3A,B). Given these results, we hypothesize that αSyn-associated lipid dysregulation alters LD organization, leading to remodeling of PLIN-1 distribution. Supporting this hypothesis, LDs are smaller in αSyn-YFP worms and with clumpy structures at day 11 of adulthood (Fig. EV3C). Notably, physical contact or interaction between αSyn inclusions and LDs is observed at day 1 of adulthood (Fig. EV3D) with no apparent co-localization by day 5 of adulthood (Figs. 3C and EV3A). These data suggest that TAG depletion and PLIN-1 mislocalization are not solely mediated by direct physical interactions. Instead, these disruptions are more likely to reflect a broader lipid metabolic dysfunction induced by αSyn condensates.

### αSyn-induced reductions in TAG levels and impaired motility are indirectly linked

**Figure EV4:**
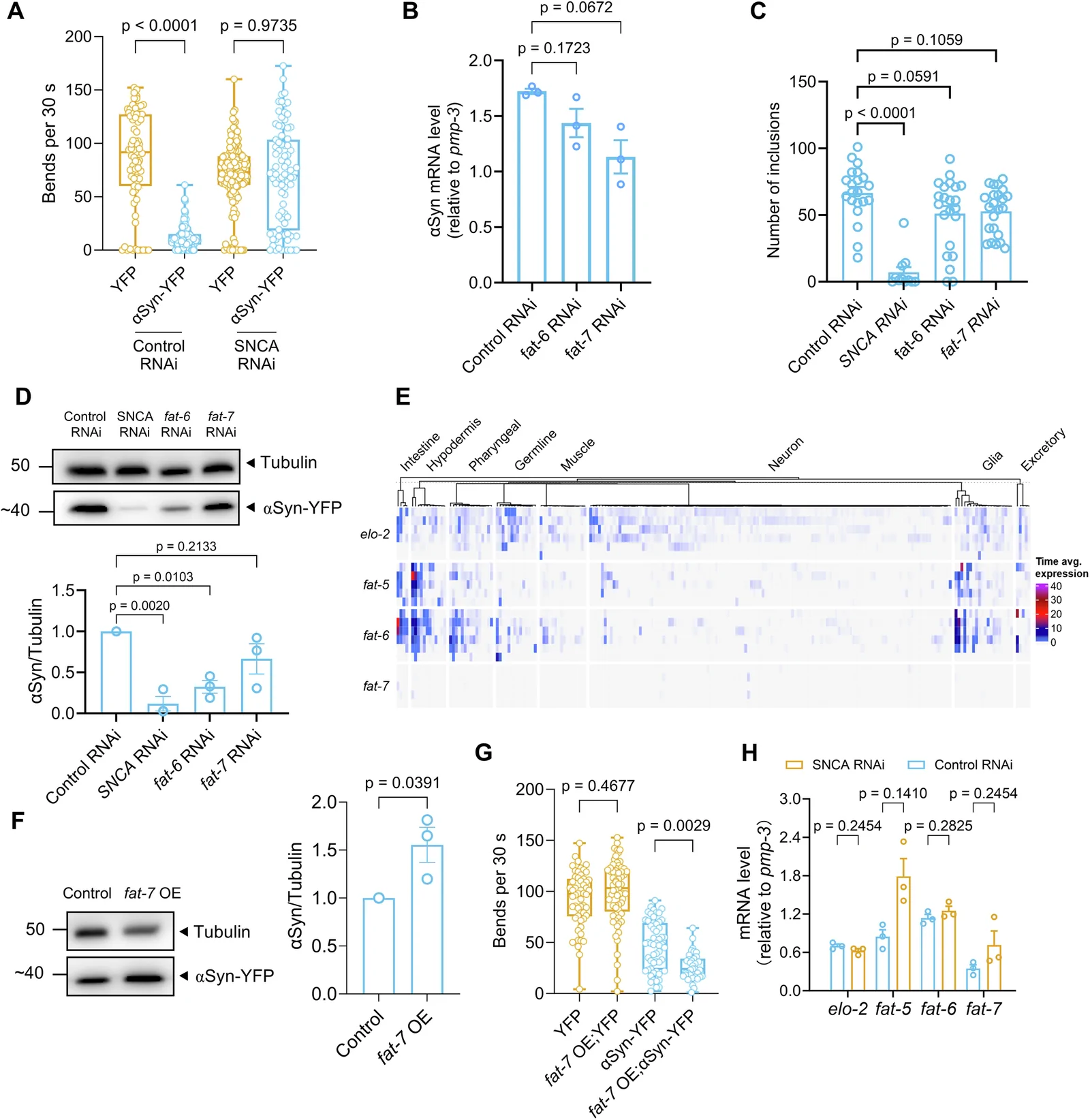
Differential effects of *fat-6* and *fat-7* modulation on αSyn toxicity. (**A**) αSyn-YFP worms exhibit significantly reduced body-bend frequency compared with YFP controls at day 5 of adulthood. Box plots show the median, IQR, and min–max whiskers (*n* = 50–100 animals per condition; one-way ANOVA). (**B**) qPCR analysis shows that *fat-6* or *fat-7* RNAi does not significantly alter *SNCA* mRNA levels in day-5 adult αSyn-YFP worms (one-way ANOVA, error bars represent s.e.m.). (**C**) Quantification of αSyn inclusions shows no significant change following *fat-6* or *fat-7* RNAi in day-5 adult αSyn-YFP worms (one-way ANOVA, error bars represent s.e.m.). (**D**) Western blot analysis shows that *fat-6* RNAi reduces αSyn protein abundance in day-5 adult αSyn-YFP worms (one-way ANOVA, error bars represent s.e.m.). (**E**) Tissue-resolved expression analysis in wild-type worms shows broad and relatively high *fat-6* expression across multiple tissues, whereas *fat-7* expression is low or undetectable in most examined tissues. (**F**) *fat-7* overexpression (OE) increases αSyn protein abundance in αSyn-YFP worms (unpaired *t* test, error bars represent s.e.m.). (**G**) *fat-7* OE exacerbates the motility defect of αSyn-YFP worms. Box plots show the median, IQR, and min–max whiskers (*n* = 50–100 animals per condition; one-way ANOVA). (**H**) Expression levels of *fat-6* and *fat-7* are not significantly altered by *SNCA* RNAi (one-way ANOVA, error bars represent s.e.m.). All experiments were performed in triplicate. Source data are available online for this figure.

**Figure 4:**
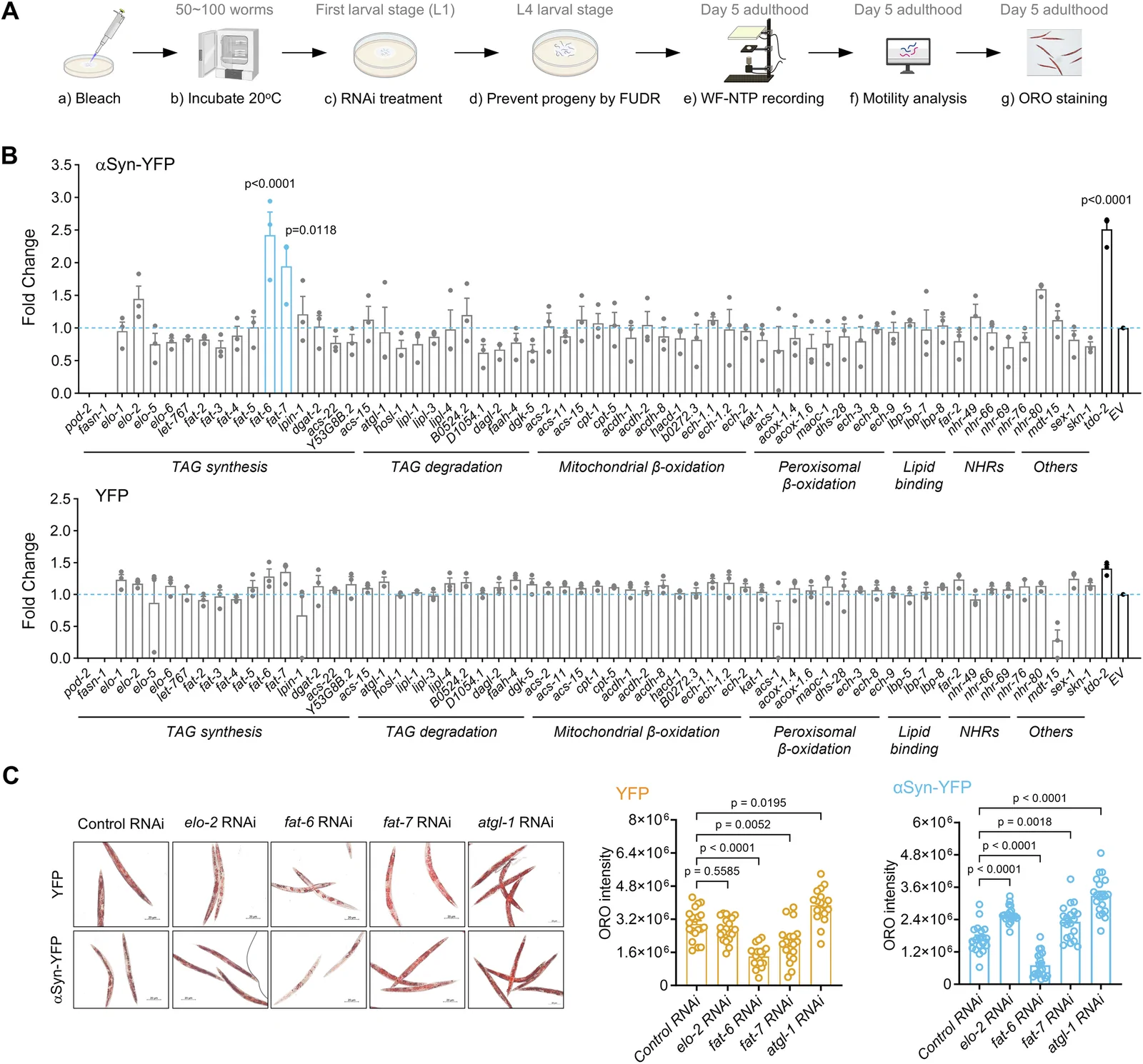
Inhibition of enzymes involved in long-chain unsaturated lipid synthesis reduces αSyn toxicity. (**A**) Experimental design for RNAi screening. Synchronized L1-stage worms were cultured on RNAi plates at 20 °C. FUDR was added at L4 stage to prevent progeny production. Motility screenings were performed in day 5 of adulthood. (**B**) RNAi-mediated knockdown of *fat-6* and *fat-7* (blue) significantly reduces αSyn toxicity in day 5 adult αSyn-YFP worms but not in YFP control worms. Motility was quantified as body bends per 30 s. Empty vector (EV) serves as a negative control and *tdo-2* RNAi as a positive control. Data represent fold changes from three biological replicates (one-way ANOVA, error bars represent s.e.m). (**C**) ORO staining shows *fat-6* and *fat-7* RNAi reduce TAG levels in YFP controls at day 5 adulthood, while *fat-7* RNAi restore TAG level in αSyn-YFP worms. *atgl-1* RNAi increases TAG in both strains (*n* = 15–20 animals; one-way ANOVA, error bars represent s.e.m.). One representative image is shown in each condition. All experiments were performed in triplicate. Source data are available online for this figure.

**Figure EV5:**
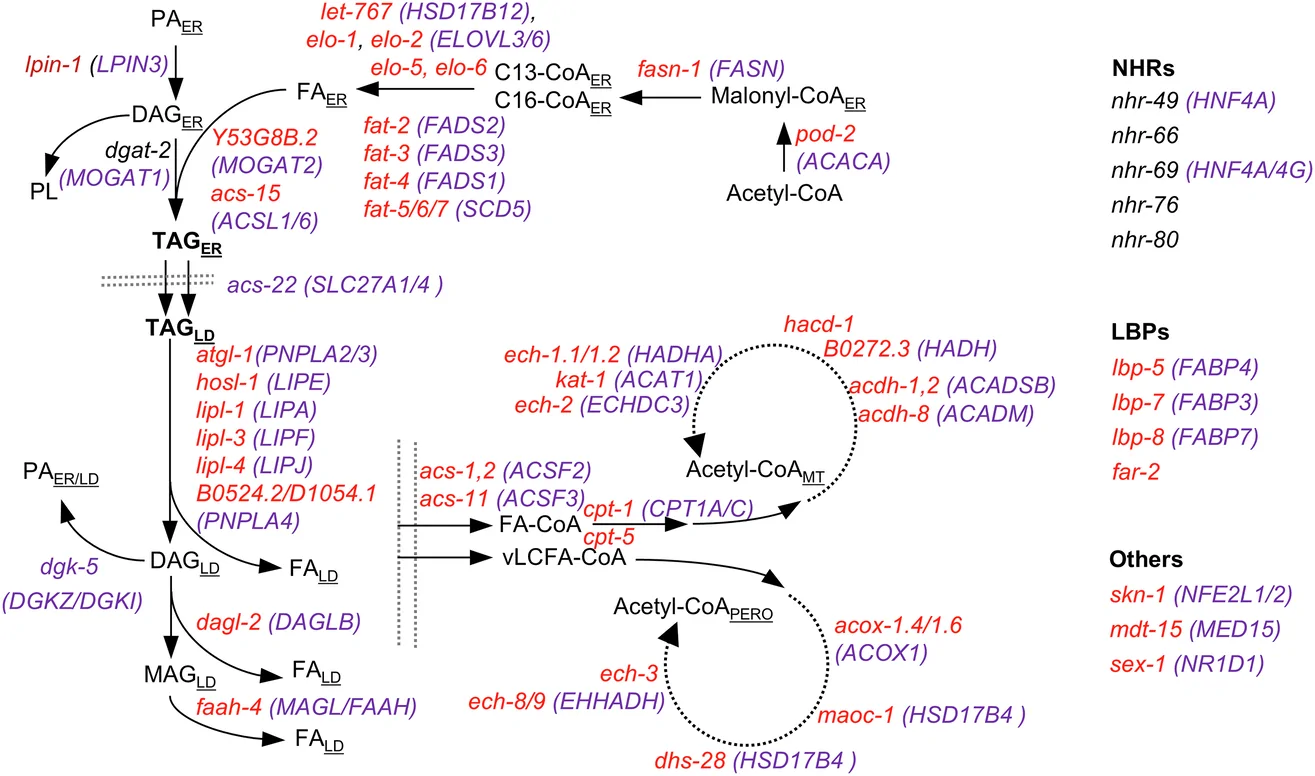
The lipid metabolism pathway in *C. elegans*. Human homologs of worm genes (red) are shown in purple.

We also detected significantly decreased motility at day 5 of adulthood in αSyn-YFP worms compared to YFP controls, a phenotype that was rescued by *SNCA* RNAi (Fig. EV4A). To explore the underlying mechanisms, we leveraged our established automated wide-field-of-view tracking platform to perform a targeted RNAi screen for TAG metabolism genes (Fig. 4A) (Koopman et al, 2020). A schematic representation of the TAG metabolic pathways is shown in Fig. EV5. Tryptophan 2,3-dioxygenase (*tdo-2*) RNAi, which was previously shown to enhance motility (van der Goot et al, 2012), served as the positive control, while empty vector (EV) functioned as the negative control. Among all tested genes, we found that knockdown of the stearoyl-CoA desaturases *fat-6* and *fat-7* significantly rescued the impaired motility in αSyn-YFP worms (Fig. 4B). These enzymes play critical roles in long-chain unsaturated fatty acids (LCUFAs) and downstream synthesis of other fatty acids and complex lipids. We also observed modest improvement with knockdown of genes critical for LCUFAs synthesis, such as *elo-2*, a fatty acid elongase enzyme, and the nuclear hormone receptor (*nhr-80*), a known transcriptional regulator of *fat-6* and *fat-7* desaturases (Brock et al, 2006). Interestingly, restoration of total TAG content by *atgl-1* RNAi failed to rescue motility (Fig. 4B,C). These results indicate that the impaired mobility induced by αSyn is independent of the total levels of TAGs but may depend on their properties, such as their length and saturation levels.

In contrast, *fat-6* knockdown significantly reduced TAG levels in both YFP and αSyn-YFP worms, whereas *fat-7* RNAi restored TAG levels in αSyn-YFP worms (Fig. 4C). Although the αSyn mRNA level and inclusion formation were not significantly altered by *fat-6* RNAi (Fig. EV4B,C), the significant reductions in worm size, TAG content, and αSyn protein abundance (Figs. 4C and EV4D) suggest that the beneficial effects of *fat-6* RNAi may reflect broader systemic consequences rather than a specific suppression of αSyn toxicity. To further investigate the distinct effects of *fat-6* and *fat-7* RNAi, we examined their basal expression patterns using a single-cell RNA-seq atlas (Roux et al, 2023). As shown in Fig. EV4E, *fat-6* is broadly expressed across multiple tissues, whereas *fat-7* exhibits substantially lower basal expression in the profiled tissues, including body-wall muscle. These differences in expression pattern may contribute to the distinct phenotypic consequences of *fat-6* and *fat-7* depletion. In particular, widespread inhibition of *fat-6* may perturb multiple physiological processes, consistent with the observed reduction in body size.

Conversely, *fat-7* overexpression significantly impaired motility, although a contribution from the modest increase in αSyn abundance in *fat-7* overexpressing worm cannot be excluded (Fig. EV4F,G). However, *SNCA* knockdown did not selectively alter *fat-6* or *fat-7* transcription (Fig. EV4H). Together, these findings argue against a global transcriptional reprogramming of TAG metabolism as the primary explanation for the TAG phenotype, suggesting that selective depletion of certain lipid species may contribute to αSyn-associated lipid dysregulation.

### Suppressing biosynthesis of long-chain unsaturated fatty acids mitigates αSyn toxicity

**Figure 5:**
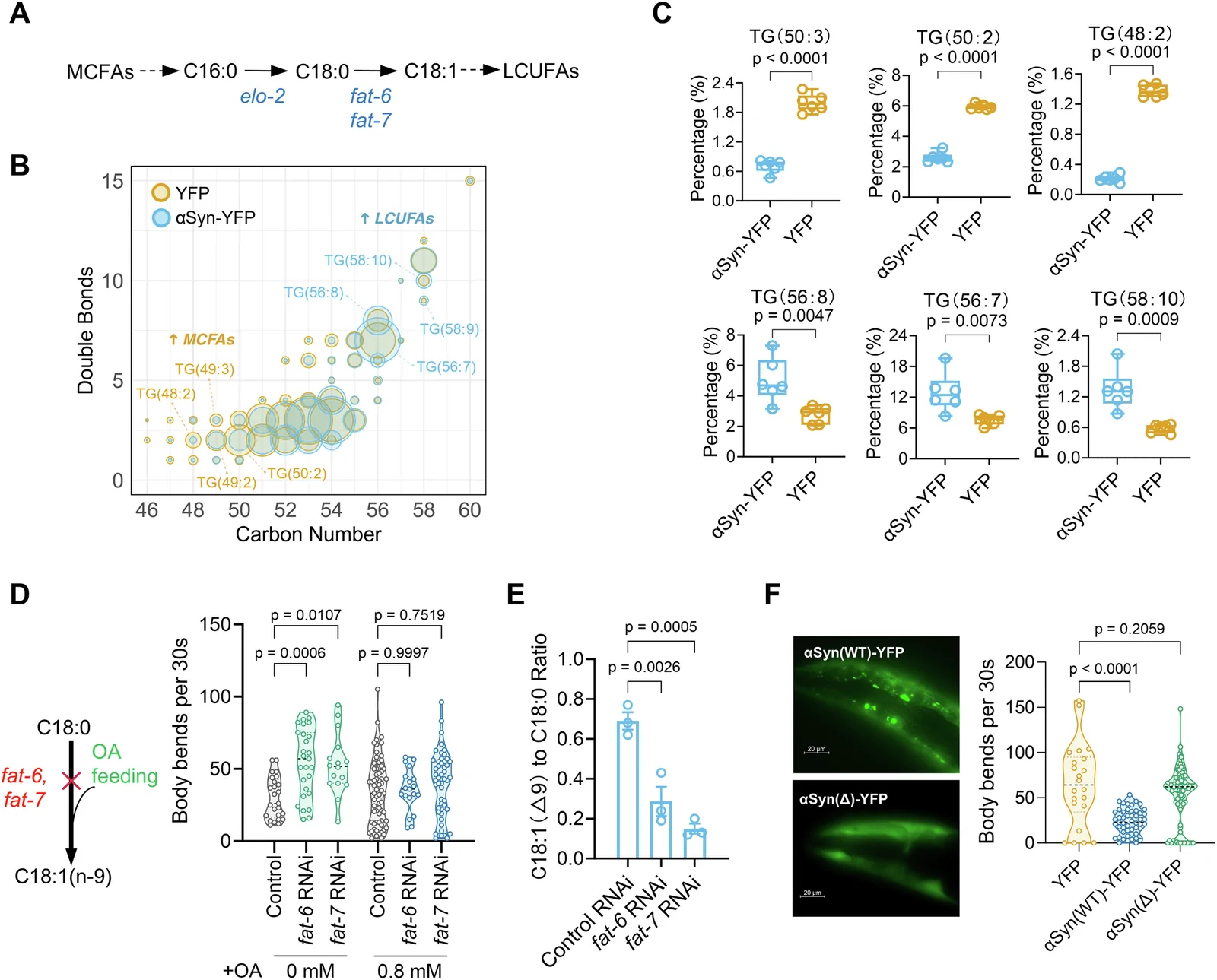
Suppression of enzymes involved in long-chain unsaturated fatty acids (LCUFAs) biosynthesis mitigates αSyn toxicity. (**A**) Schematic of the fatty acid metabolic pathway illustrating the roles of *fat-6*, *fat-7*, and *elo-2* in the biosynthesis of long-chain unsaturated fatty acids (LCUFAs) from medium-chain saturated fatty acids (MCFAs). (**B**) Lipidomic analysis reveals αSyn-YFP worms proportionally elevates LCUFA-containing TAGs (blue) versus YFP controls (MCFAs-enriched, yellow). (**C**) Percentage of six predominant TAG molecular species shows significant higher unsaturation in αSyn-YFP worms compared to YFP controls. Box plots show the median, IQR, and min–max whiskers (*n* = 6, unpaired *t* test). (**D**) Oleic acid (OA, C18:1) as LCUFA supplementation abolishes the protective effects of *fat-6* and *fat-7* RNAi, failing to rescue motility in αSyn-YFP worms (one-way ANOVA, Violin plot with all points). (**E**) Quantification of C18:1 fatty acid abundance showing that *fat-6* and *fat-7* RNAi reduce C18:1 levels in αSyn-YFP worms (*n* = 3, unpaired *t* test, error bars represent s.e.m.). (**F**) Deletion of αSyn lipid-binding domain (PVH198 strain, αSyn(Δ)-YFP) forms no inclusions and rescues motility defects of αSyn-YFP worms (one-way ANOVA, Violin plot with all points). All experiments were performed in triplicate. Source data are available online for this figure.

**Figure EV6:**
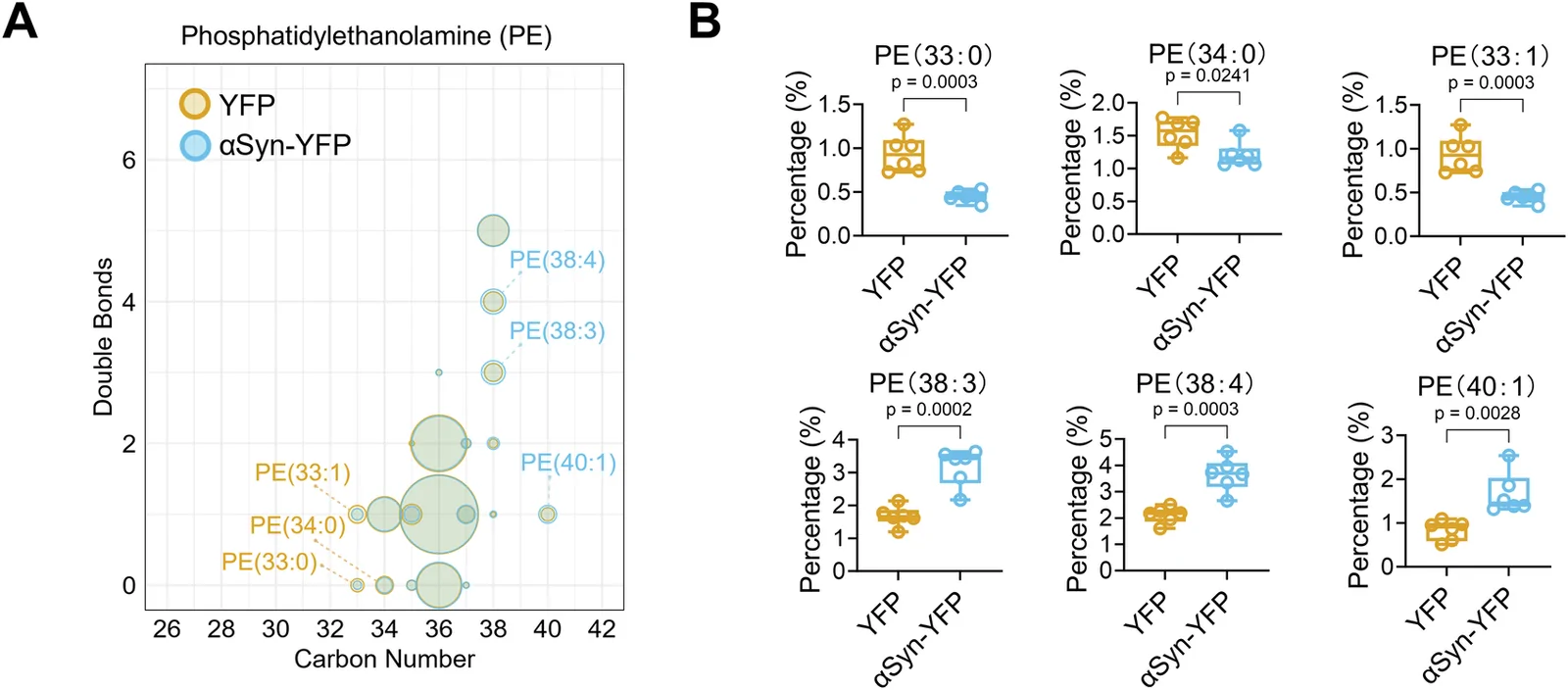
αSyn proportionally increases unsaturation of fatty acyl chains in phosphatidylethanolamine. (**A**) Lipidomics data demonstrates proportionally elevated unsaturation index (blue) in phosphatidylethanolamine (PE) species from αSyn-YFP versus YFP worms. (**B**) Percentage of six predominant PE molecular species shows higher unsaturated composition in αSyn-YFP worms compared to YFP controls. Box plots show the median, IQR, and min–max whiskers. Each lipidomics sample contained approximately 3000 worms collected from six replicate plates. Source data are available online for this figure.

Elongation and desaturation of medium-chain saturated fatty acids (MCFAs) into LCUFAs is mediated by *elo-2*, *fat-6* and *fat-7* (Fig. 5A). In our lipidomic analysis, we detected a higher proportion of LCUFAs associated with both TAG and phosphatidylethanolamine (PE), an abundant phospholipid in mammalian cells, in αSyn-YFP worms (Figs. 5B,C and EV6A,B). These data confirm that αSyn toxicity is not solely due to the reduction in total TAG levels, but is also influenced by the specific composition of its fatty acyl chains. When we fed worms with oleic acid (OA), a downstream product of *fat-6* and *fat-7* (Watts and Ristow, 2017), the protective effects of *fat-6* or *fat-7* RNAi were abolished (Fig. 5D). We further performed targeted fatty acids analyses in αSyn-expressing worms. Consistently, *fat-6* and *fat-7* RNAi significantly reduced the ratio of LCUFAs, particularly C18:1(n-9) to C18:0, in αSyn-YFP worms (Fig. 5E). Taken together, these results indicate that accumulation of LCUFAs contributes to αSyn-induced pathology and that limiting LCUFAs biosynthesis might mitigate αSyn toxicity.

It’s widely accepted that LCUFAs physically interact with αSyn in a way that modulates its toxic properties. When we examined the motility of the αSyn(Δ) strain, a transgenic αSyn strain carrying a specific deletion of its membrane-binding domain (Doherty et al, 2020), we noted significantly higher motility compared to the αSyn(WT)-YFP control (Fig. 5F). These data support that the membrane or fatty acid interactions are a crucial determinant of αSyn-induced toxicity, suggesting that αSyn requires membrane binding to reduce motility of the worms.

### Bypassing LCUFAs biosynthesis via MCT feeding rescues αSyn toxicity by restoring mitochondrial function

**Figure 6:**
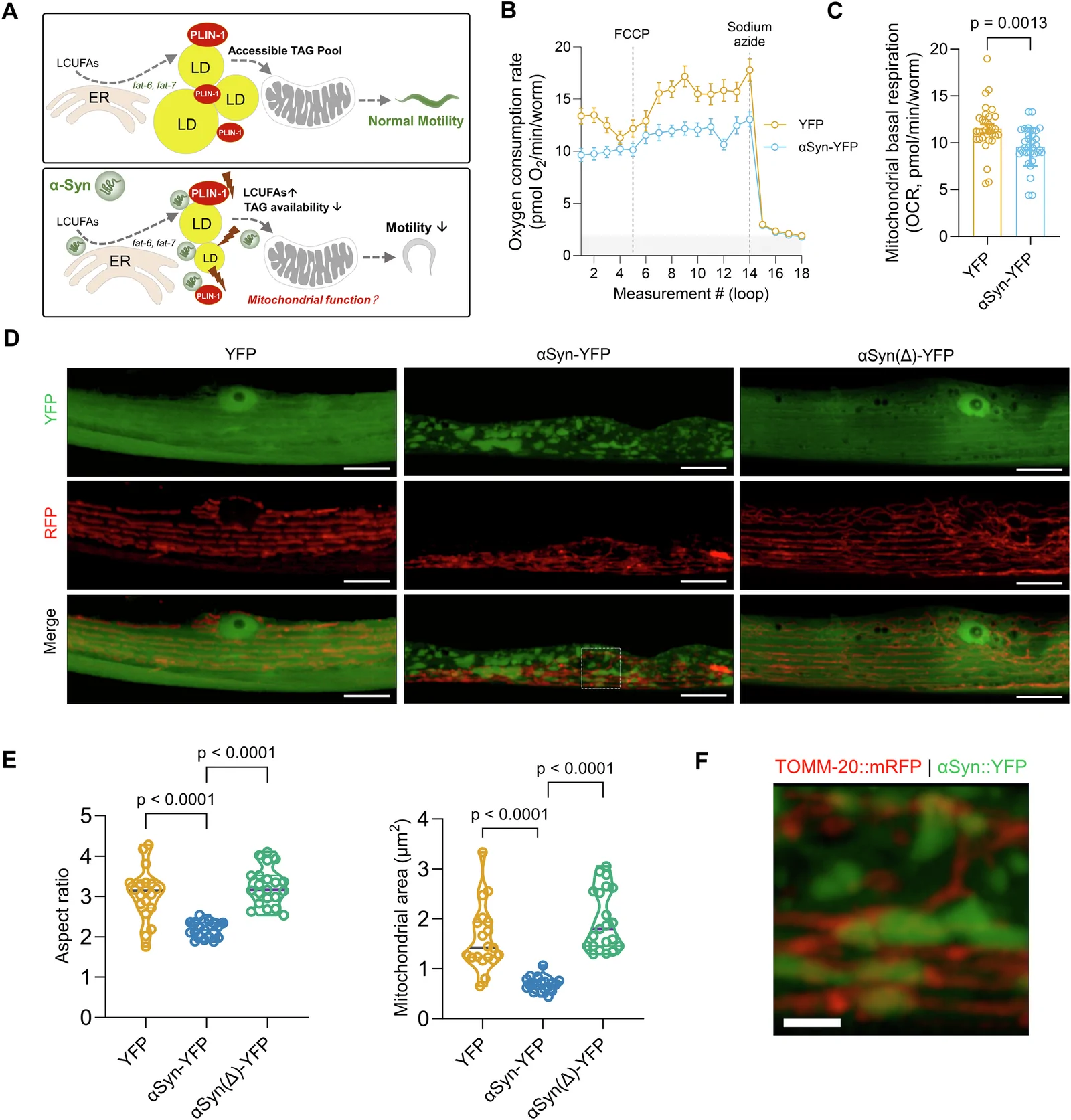
αSyn impairs mitochondrial function and promotes fragmentation in a lipid-binding-dependent manner. (**A**) Proposed model illustrating the roles of αSyn inclusion formation in disrupting TAG metabolism. In αSyn-YFP worms, αSyn inclusion formation is associated with impaired LCUFAs ratio, LD biogenesis and PLIN-1 mislocalization, potentially disrupting TAG availability and mitochondrial function. (**B**) Oxygen consumption rate (OCR) analysis shows reduced basal mitochondrial respiration in day-5 adult αSyn-YFP worms compared with YFP controls (*n* = 12 wells, error bars are s.e.m.). (**C**) Quantification of basal respiration confirms a significant reduction in mitochondrial respiratory capacity in αSyn-YFP worms compared with YFP controls (unpaired *t* test, error bars represent s.e.m). (**D**) Representative TOMM-20::mRFP images reveal increased mitochondrial fragmentation in αSyn(WT)-YFP worms, whereas this phenotype is not apparent in αSyn(Δ)-YFP worms expressing a lipid-binding-deficient αSyn variant. Scale bar, 10 μm. (**E**) Quantification of mitochondrial morphology shows reduced mitochondrial aspect ratio and area in αSyn(WT)-YFP worms compared with YFP and αSyn(Δ)-YFP worms at day 5 of adulthood (*n* ≥ 19 animals, one-way ANOVA, Violin plot with all points). (**F**) Representative image showing spatial co-localization of αSyn inclusions with mitochondria in αSyn-YFP worms. Scale bar, 2 μm. (**B**, **C**) Data were combined from three independent replicates. (**D**, **F**) One representative image is shown in each condition. Source data are available online for this figure.

**Figure EV7:**
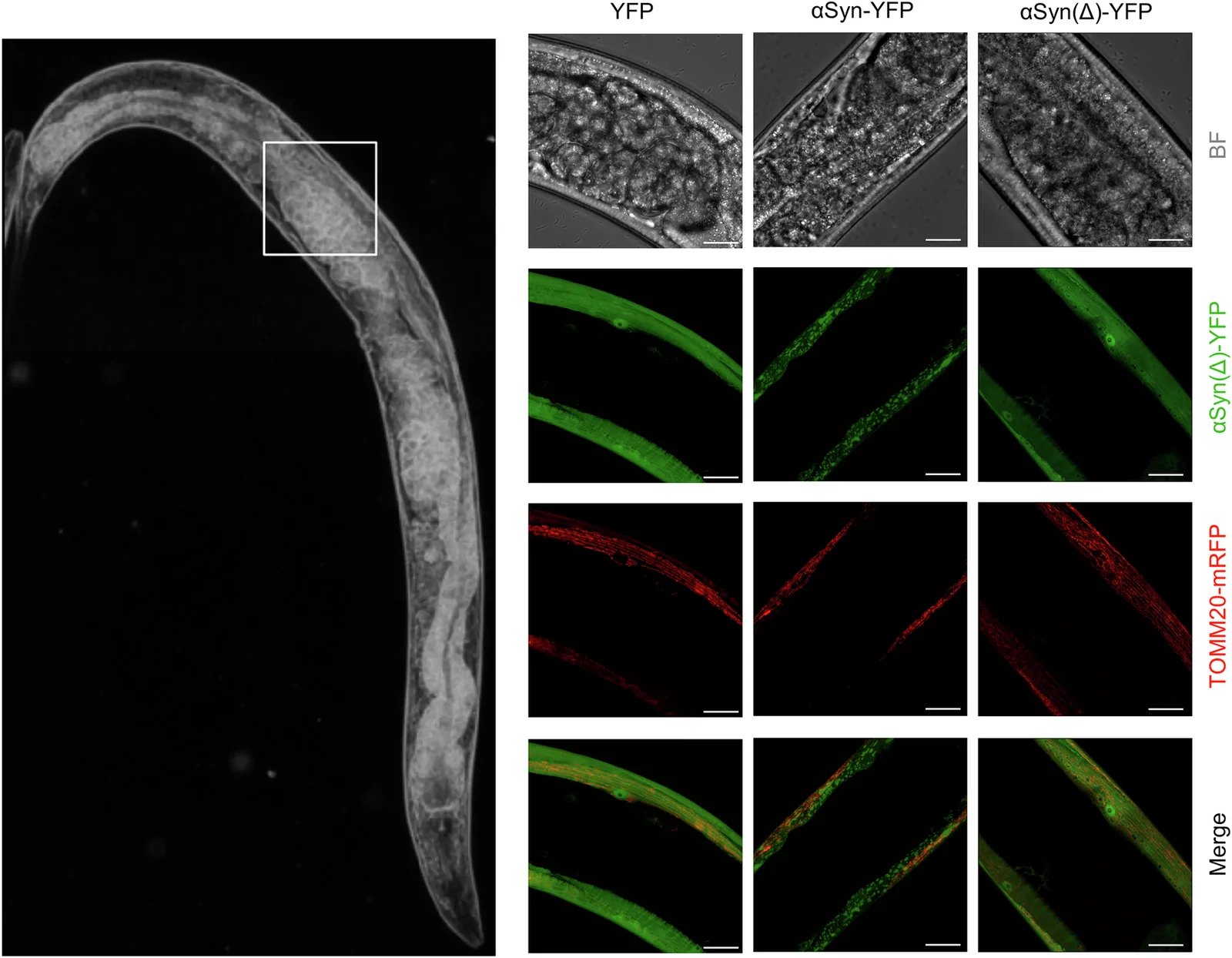
Expression of αSyn(WT)-YFP, but not αSyn(Δ)-YFP, is associated with increased mitochondrial fragmentation. Overview images of the regions used for mitochondrial morphology analysis. Scale bar, 20 µm. Images correspond to the wider-field views of those shown in Fig. 6D. Source data are available online for this figure.

Given that PLIN-1 is an important regulator of LD lipolysis and fatty-acid mobilization, disruption of PLIN-1 localization could potentially perturb the availability of LD-derived fatty acids for mitochondrial metabolism (Sztalryd and Brasaemle, 2017). We propose a mechanistic model in which αSyn inclusions disrupt LD intergrity and PLIN-1 localization, thereby reducing TAG availability and compromising mitochondrial function, ultimately leading to decreased motility (Fig. 6A). Using our established platform for assessment of basal mitochondrial respiration (Koopman et al, 2016), we found that the mitochondrial respiratory capacity is diminished in αSyn-YFP worms (Fig. 6B), particularly in basal respiratory capacity (Fig. 6C). Additionally, we observed a fragmented pattern with lower aspect ratio and mitochondrial area in αSyn-YFP worms but not in αSyn(Δ)-YFP worms, as visualized by TOMM-20::mRFP (Figs. 6D,E and EV7). αSyn inclusions were frequently observed in close proximity to mitochondria, with partial co-localization between αSyn and mitochondrial markers (Fig. 6F). Collectively, these observations suggest that αSyn aggregates may be associated with mitochondria and mitochondrial morphological abnormalities, potentially mediated by the lipid-binding properties of αSyn.

**Figure 7:**
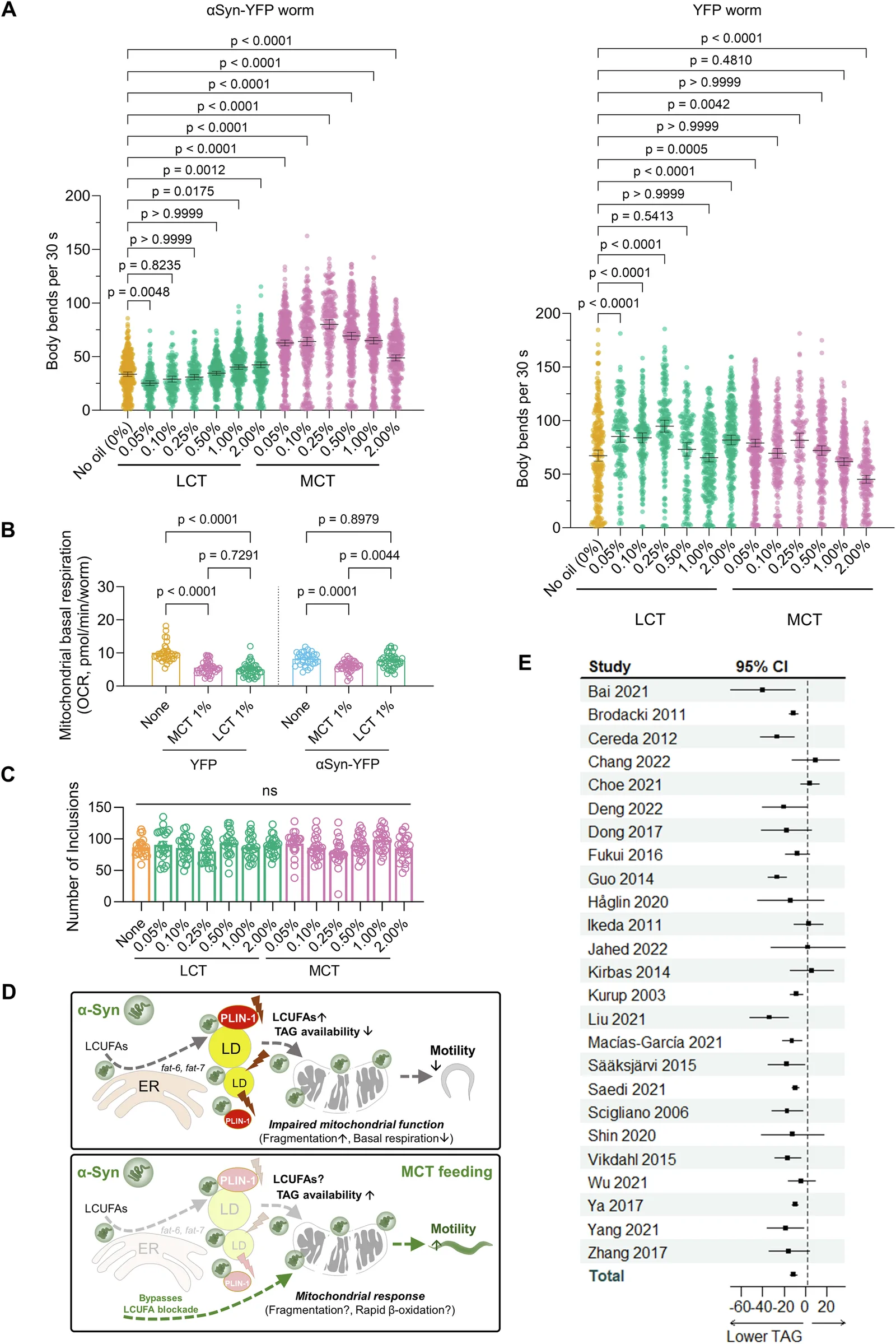
Bypassing LCUFAs synthesis via MCFAs feeding mitigates αSyn-associated toxicity. (**A**) MCT feeding dose-dependently rescues motility defects in αSyn-YFP worms but impairs motility in YFP controls at high doses (one-way ANOVA, error bars represent mean with 95% CI). (**B**) MCT supplementation reduces basal mitochondrial respiration in day-5 adult αSyn-YFP worms compared with LCT supplementation, consistent with an altered metabolic state rather than restoration of respiratory capacity (one-way ANOVA, error bars represent s.e.m.). (**C**) MCT and LCT supplementation did not significantly alter the number of αSyn inclusions in day-5 adult αSyn-YFP worms (one-way ANOVA, error bars represent s.e.m.). (**D**) Proposed model illustrating that MCT supplementation bypasses LCUFAs-dependent lipid metabolism and provides MCFAs that can be rapidly utilized by mitochondria, thereby promoting metabolic adaptation and improving motility. In the absence of MCT supplementation, αSyn-YFP worms exhibit mitochondrial dysfunction characterized by increased mitochondrial fragmentation and reduced basal respiration. (**E**) Forest plot of pooled clinical data showing significantly lower circulating TAG levels in patients with Parkinson’s disease (PD; *n* = 2958) than in healthy controls (*n* = 10,112). (**B**) Data were combined from three independent replicates. Source data are available online for this figure.

**Figure EV8:**
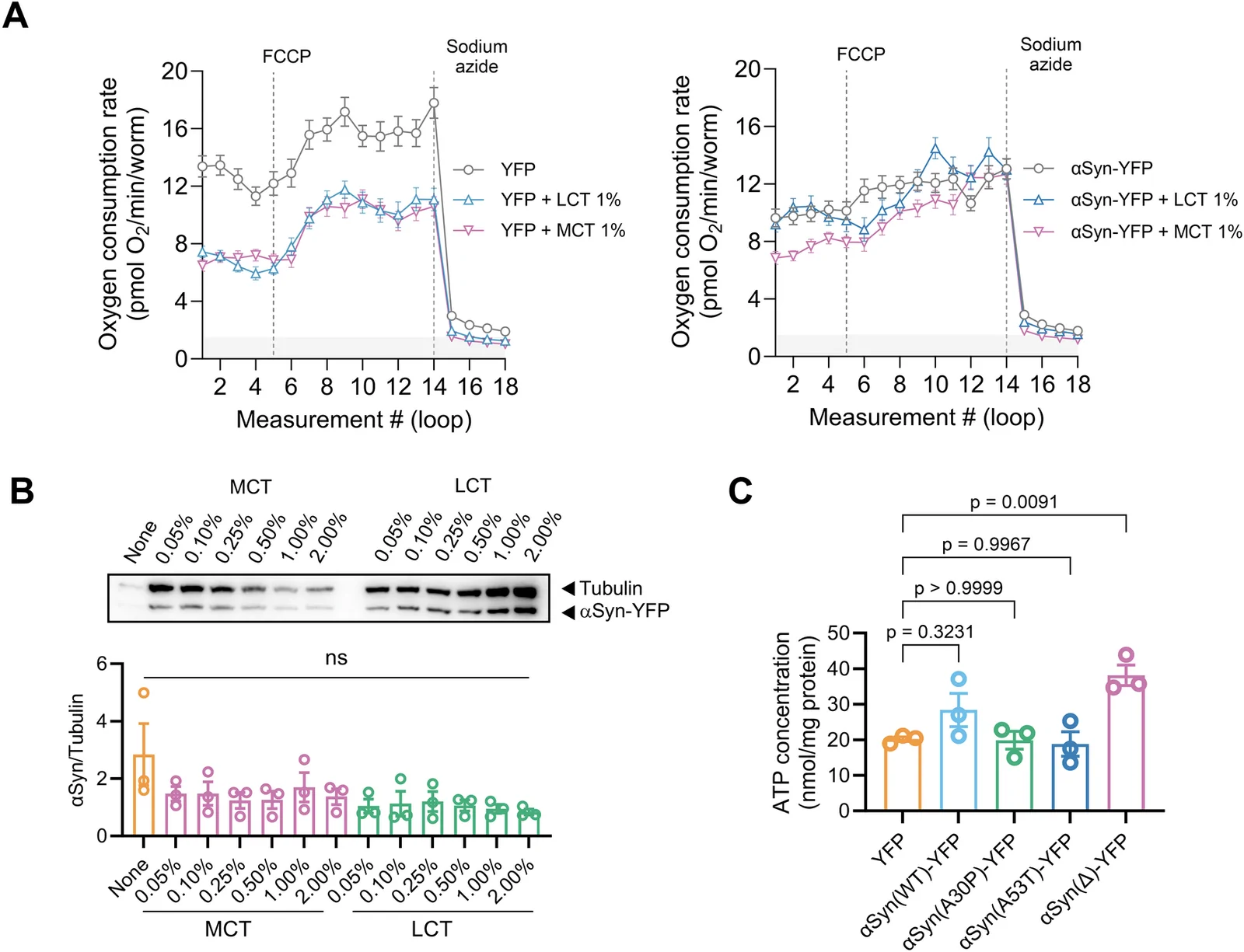
Oxygen consumption rate (OCR) measurements show that αSyn-YFP worms respond selectively to MCT supplementation, but not LCT supplementation. (**A**) Oxygen consumption rate (OCR) measurements show that αSyn-YFP worms respond selectively to MCT supplementation, but not LCT supplementation, whereas YFP control worms respond to both MCT and LCT supplementation (*n* = 12 wells, error bars represent s.e.m.). (**B**) MCT and LCT supplementation do not significantly alter αSyn protein abundance in αSyn-YFP worms (one-way ANOVA, error bars represent s.e.m.). (**C**) αSyn variants with intact membrane-binding capacity do not significantly alter ATP abundance compared with YFP controls, but αSyn(Δ)-YFP animals show significantly increased ATP abundance relative to YFP controls (one-way ANOVA, error bars represent s.e.m.). All experiments were performed in triplicate. Source data are available online for this figure.

As our data implicate LCUFAs in αSyn toxicity, we next asked whether bypassing LCUFAs utilization through dietary supplementation with MCTs could alleviate the associated functional defects. Unlike LCTs, MCT composed predominantly of C8–C12 medium-chain fatty acids, are more readily oxidized and can enter mitochondrial β-oxidation with reduced dependence on the carnitine shuttle (Rinaldo et al, 2002). As shown in Fig. 7A, MCT supplementation markedly improved motility in αSyn-YFP animals, with the strongest rescue observed at intermediate concentrations. In contrast, MCT supplementation did not enhance motility in YFP control animals and, at higher concentrations, was associated with reduced motility. This differential response indicates that MCT supplementation preferentially rescues the αSyn-associated motility defect rather than exerting a general motility-enhancing effect. Notably, the effects of MCT also differed from those of LCT. Although LCT supplementation modestly altered motility in both genotypes, it did not reproduce the robust rescue observed with MCT in αSyn-YFP animals. This result reflects a distinct metabolic properties of MCFAs. Consistent with this distinction, mitochondrial respiration measurements showed that αSyn-YFP worms exhibited a respiratory response to MCT supplementation but not to LCT, whereas YFP controls responded to both lipid sources (Fig. 7B). At 1% MCT, αSyn-YFP worms displayed reduced basal respiration, similar to the decrease observed in YFP animals following either MCT or LCT supplementation. Rather than necessarily indicating impaired mitochondrial function, this reduction in steady-state oxygen consumption may reflect altered substrate utilization or metabolic flux. Together, these findings suggest that MCT and LCT are metabolically distinct in the αSyn-YFP worms, whereas YFP worms retain the capacity to respond to both lipid sources, αSyn-YFP animals preferentially respond to MCT, consistent with an impairment in long-chain lipid utilization that can be partially bypassed by MCFAs (Figs. 7B and EV8A,B). No significant differences in inclusion number were observed following either MCT or LCT supplementation (Fig. 7C). The αSyn-YFP protein levels were also not changed across the tested concentration range (Fig. EV8B). These results indicate that the beneficial effects of MCT are unlikely to be mediated by reducing αSyn expression or suppressing αSyn aggregation.

We also examined ATP levels in worms expressing different αSyn variants with distinct membrane-binding properties. Only αSyn(Δ)-YFP worms exhibited increased ATP abundance compared to YFP worms (Fig. EV8C), suggesting that loss of membrane-binding capacity preserves cellular metabolic status, consistent with reduced toxicity observed in αSyn(Δ)-YFP animals. However, the protective effect of αSyn(Δ)-YFP is unlikely to be explained solely by ATP production, but instead reflects prevention of upstream membrane-associated disturbances that affect lipid homeostasis, organelle organization, or mitochondrial function. These results support a broader mechanism in which αSyn toxicity is driven primarily by disruption of lipid homeostasis and metabolic adaptation. In all, these results demonstrate that αSyn condensation is associated with perturbation of intracellular lipid and organelle homeostasis. In particular, the mitochondrial phenotype provides evidence that αSyn toxicity is not restricted to LDs and suggests that mitochondrial dysfunction may represent an additional cellular consequence of αSyn toxicity (Fig. 7D). Our results suggest that MCT’s bypass specific metabolic constraints associated with long-chain lipid utilization and promote a more adaptive metabolic state under αSyn-induced lipid dysregulation.

**Figure EV9:**
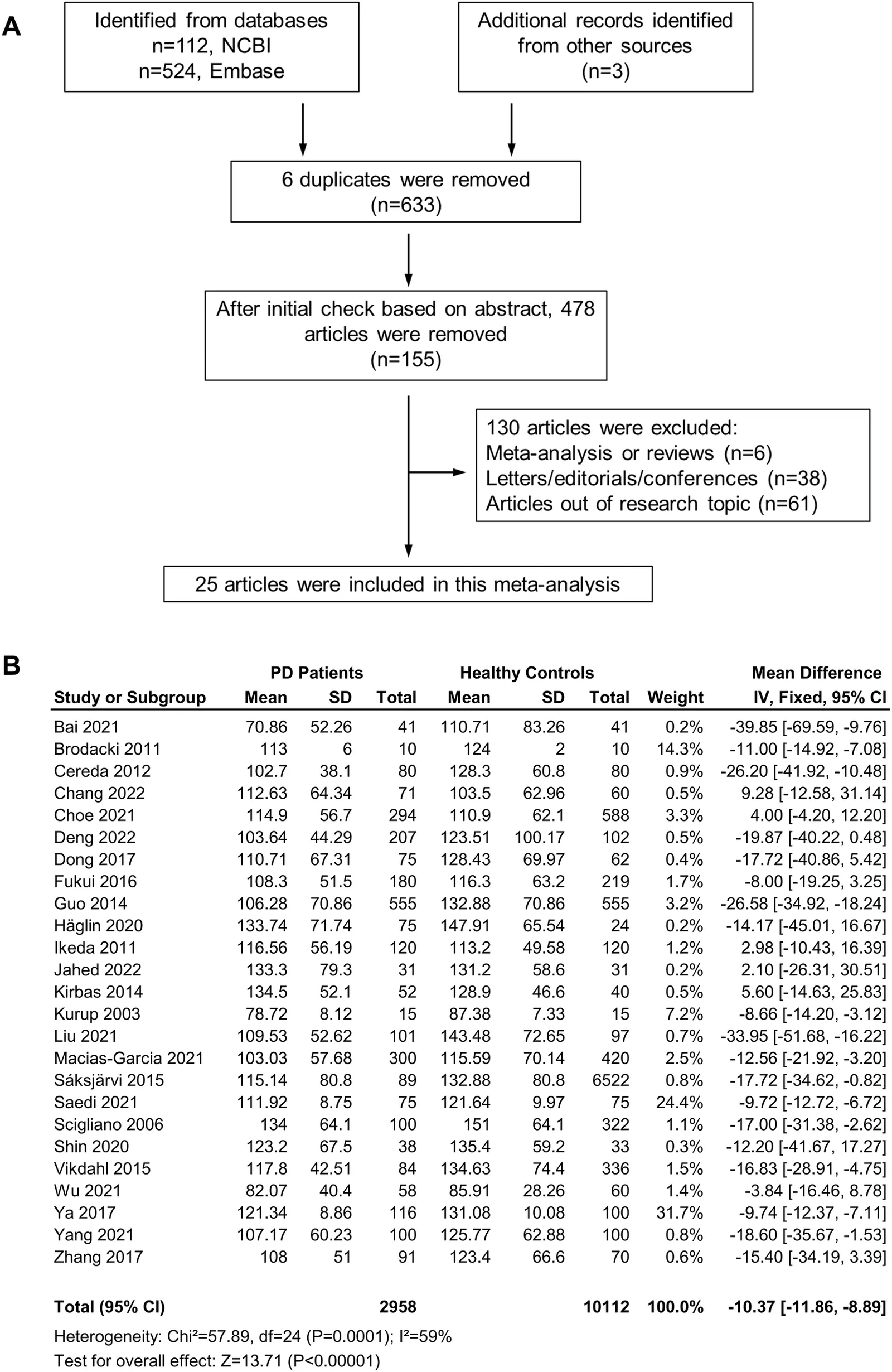
Reduced serum TAG levels in patients with Parkinson’s disease based on a systematic meta-analysis. (**A**) Flow diagram of study selection process for the meta-analysis, showing identification, screening, eligibility assessment, and final inclusion of studies. (**B**) Forest plot of pooled results demonstrating significantly lower serum TAG levels in PD patients (*n* = 2958) compared to healthy controls (*n* = 10,112). The standardized mean difference with 95% confidence intervals is shown for each study and overall effect size (random-effects model). Source data are available online for this figure.

Finally, we conducted a systematic analysis of 25 clinical studies involving 2958 PD patients and 10,112 healthy controls to evaluate potential relevance of our findings in humans (Fig. EV9A). Indeed, we found that serum TAG levels were significantly lower in PD patients compared to healthy controls (Figs. 7E and EV9B). Supporting our conclusion that reduced food intake does not lower TAG levels, a previous study also reported a reduced whole-body fat mass index in PD patients despite comparable dietary energy intake (Tan et al, 2018). In all, decreased TAG levels appear to represent a conserved feature shared between *C. elegans* PD models and clinical PD.

## Discussion

αSyn pathophysiology is closely associated with disrupted lipid homeostasis, although the underlying cellular mechanisms remain incompletely understood (Stok and Ashkenazi, 2020). Here, we identified a conserved association between αSyn expression and TAG depletion in *C. elegans* models and PD patient serum datasets. Systematic analysis of clinical studies revealed consistently lower fasting serum TAG concentrations in PD patients compared with healthy controls. Our findings suggest that αSyn condensate formation is associated with broader disruption of lipid homeostasis, characterized by reduced TAG abundance and altered LCUFAs composition. Rather than indicating that TAG depletion alone drives toxicity, these changes may represent features of a wider lipid imbalance contributing to αSyn-associated cellular dysfunction.

Supplementation with MCT, promotes a more adaptive metabolic state and rescues pathological effects by bypassing the need for TAG mobilization. As TAG mobilization from LDs is essential for supplying FFAs required for beta-oxidation as well as for mitochondrial biogenesis (Baker et al, 2025), MCT improved αSyn-associated motility defects without significantly altering αSyn protein abundance or inclusion formation. These results suggest that MCT acts downstream of αSyn pathology rather than by suppressing αSyn expression or aggregation. Although MCT may influence mitochondrial substrate utilization and metabolic flexibility, our data do not establish that MCT directly restores ATP production. Instead, MCT may alleviate lipid utilization stress by providing an alternative metabolic substrate and promoting cellular adaptation.

Among the TAG metabolic genes screened, suppression of LCUFAs biosynthesis significantly reduced αSyn toxicity. The protective effect aligns with previous studies attributing the benefit to the balance between saturated and unsaturated fatty acyl chains (Imberdis et al, 2019). Stearoyl-CoA desaturase (SCD), the mammalian homolog of *fat-6* and *fat-7*, has been extensively studied in PD and related synucleinopathies (Nuber et al, 2025). Knockdown of *fat-6* or *fat-7* likely confers protection by limiting SCD-mediated desaturation of saturated fatty acids into their monounsaturated forms (C16:0 into C16:1 and C18:0 into C18:1). Although SCD inhibition-mediated low desaturation may decrease αSyn toxicity, we observed increased proportions of unsaturated PE in αSyn worms, suggesting that *fat-6* and *fat-7* knockdown may exert protective effects through multiple SCD-dependent mechanisms. These findings are consistent with the role of SCD in regulating the ratios of phosphatidylcholine (PC) to PE (Shi et al, 2013). In line with previous findings that *fat-6*-dependent products are essential for development (Han et al, 2017), *fat-6* knockdown reduced body size and modestly improved motility in control worms, while significantly lowering αSyn expression and TAG levels. These findings suggest that the protective effect of *fat-6* knockdown reflect systemic metabolic changes rather than αSyn-specific toxicity suppression. In contrast, *fat-7* overexpression significantly increased αSyn levels and severely impaired motility without affecting body size, consistent with a distinct and possibly more beneficial role for *fat-7* in modulating αSyn toxicity. Together, these results reveal an unrecognized functional divergence between the two SCD isoforms in αSyn toxicity. Further studies are needed to determine whether *fat-6* and *fat-7* act in a tissue-specific manner or under distinct physiological conditions. For example, *fat-7*, but not *fat-6*, is required for maintaining membrane fluidity under stress (Fox et al, 2024). Additionally, whether the observed TAG depletion results from decreased de novo fatty acid synthesis or enhanced mobilization remains unclear.

We observed altered PLIN-1 organization in αSyn-expressing worms, which serves as a marker of altered LD organization rather than direct evidence of PLIN-1 dissociation from intact LDs or physical displacement by αSyn inclusions. Given that PLIN-1 is a LD-associated structural protein involved in LD stability and lipid storage, this redistribution may reflect remodeling of LD architecture associated with disrupted TAG homeostasis. As PLIN-1 facilitates FFA release from LDs for mitochondrial β-oxidation, its mislocalization could impair TAG storage and lead to premature lipolysis. This finding is consistent with recent studies indicating that TAGs act as a critical energy reserve for neuronal synaptic function (Kumar et al, 2025), with efficient FFA mobilization from LDs being essential for local mitochondrial energy production. Collectively, these findings suggest that αSyn condensation leads to a broader disruption of TAG homeostasis that extends beyond its immediate site of expression.

Interestingly, while αSyn inclusions occasionally colocalized with LDs at early developmental stages, we did not observe consistent co-localization at later stages, implicating mechanisms beyond direct LDs binding. For example, αSyn could bind FFAs or interact with TAG-metabolizing enzymes localized outside LDs (Sanluca et al, 2024), thereby impairing LDs formation or turnover. In previous studies, αSyn was reported to inhibit TAG lipolysis by associating with LDs, leading to their accumulation (Copic et al, 2018; Outeiro and Lindquist, 2003). Consistently, we observed a co-localization pattern between αSyn and mitochondria suggesting that αSyn may impair mitochondrial function and induce fragmentation. However, deletion of αSyn’s lipid-binding domain rescued defects in motility and mitochondrial morphology. These findings underscore the critical role of interactions with lipid-rich organelles in αSyn-mediated toxicity.

Our data also shows that αSyn condensates induce mitochondrial fragmentation, warranting further investigations of mitochondrial dynamics. Additionally, αSyn may exert localized effects at other organelle contact sites, such as mitochondria-associated ER membranes (MAMs) (Del Vecchio et al, 2023). αSyn has been reported to disrupt ER-Golgi trafficking and to interact with both mitochondria and LDs (Cevallos et al, 2026; Cooper et al, 2006; Di Maio et al, 2016). These interactions interfere with the ER-mediated conversion of DAG to TAG, resulting in excess FFA with potential toxicity. Sequestering excess FFAs into LDs reduces αSyn aggregation, while limiting the incorporation of unsaturated FFAs into TAG or increasing shorter saturated fatty acyl-lipids alleviates αSyn-induced toxicity (Fanning et al, 2019; Outeiro and Lindquist, 2003; Pacheco et al, 2026; Soste et al, 2019; Tardiff et al, 2022). Collectively, these findings underscore the central role of LD imbalance in αSyn pathology, particularly involving alterations in fatty acyl chain length and saturation. Our study identifies TAG imbalance as a critical pathological feature, linking upstream αSyn-induced lipid alterations to downstream impairments in mitochondrial metabolism.

In the future, it will be important to elucidate the subcellular alterations of specific TAG species and the underlying mechanisms involving organelle interactions, as well as the structural changes induced by elevated αSyn condensation. TAGs are hydrolyzed to release FFAs, which serve as essential substrates for energy production but also function as signaling molecules during tissue repair. It is also interesting to consider whether MCTs and other structurally related TAG species support mitochondrial ATP production. Such metabolic flexibility could offer a targetable therapeutic strategy for PD and related synucleinopathies, especially under conditions in which access to other energy sources is limited. Overall, manipulating mitochondrial energetics represents a promising therapeutic approach for the treatment of PD and related disorders.

## Methods

**Table:** Reagents and tools table

Reagent/resource	Source	Identifier
**Experimental models**		
OP50::mCherry	Chelo Lab	N/A
OP50	CGC	N/A
RNAi bacteria from the Ahringer library	DNAform.jp	N/A
*C. elegans* strains; see Table EV1	This paper	N/A
**Chemicals and reagents**		
Select agar	Life Technologies	Cat# 30391049
Difco™ Casein Digest	Life Technologies	Cat# 211610
isopropylthio-β-D-galactoside, Dioxane-Free (IPTG)	Promega	Cat# V3951
Ampicillin sodium salt	Fisher Scientific	Cat# 10419313
5-Fluoro-2′-deoxyuridine (FUDR)	Sigma	Cat# F0503
Paraformaldehyde	Sigma-Aldrich	Cat# P6148
Isopropyl alcohol	Biosolve	Cat# 162678
Acetonitrile	Biosolve	Cat# 012078
Methanol	Biosolve	Cat# 136841
Methyl tert-butyl ether	Biosolve	Cat# 138906
Chloroform	Merck KGaA	Cat# 1.02444
Ammonium formate	Sigma-Aldrich	Cat# 70221
Acquity UPLC CSH column	Waters	Cat# 186005297
Dimethyl sulfoxide (DMSO)	Sigma-Aldrich	Cat# D8418
( − )-Tetramisole hydrochloride (Levamisole)	Sigma-Aldrich	Cat# L9756
Oil Red O	Sigma-Aldrich	Cat# O0625
BODIPY™ 558/568 C12	Thermofischer	Cat# D3835
TERGITOL™ solution	Sigma-Aldrich	Cat# NP40S
Oleic acid	Sigma-Aldrich	Cat# O1008
Glyceryl trioctanoate (MCT)	Sigma-Aldrich	Cat# T9126
Olive oil (LCT)	Sigma-Aldrich	Cat# O1514
ECL Prime Western Blotting Detection Reagent	Amersham	Cat# RPN2232
Nitrocellulose membrane	Bio-Rad	Cat# 1620112
TRIzol™ Reagent	Life Technologies	Cat# 15596018
Carbonyl cyanide p-trifluoromethoxyphenylhydrazone (FCCP)	Abcam	Cat# ab120081
Sodium azide	Sigma-Aldrich	Cat# S2002
Seahorse XF Calibrant Solution	Agilent	Cat# 100840-000
**Kits**		
iTaq™ Universal SYBR® Green Supermix	Bio-Rad	Cat#172-5125
Pierce™ BCA protein assay kit	Thermo Scientific	Cat# 23227
RevertAid H Minus First Strand cDNA Synthesis Kit	Life Technologies	Cat# K1632
Roche ATP Bioluminescence Assay Kit CLS II	Roche	Cat# 11699695001
**Antibodies**		
Anti-Tubulin (TUBA1) antibody	Sigma-Aldrich	Cat# T6074
Anti-Synuclein antibody [LB 509]	Abcam	Cat# ab27766
Living colors A.v. Monoclonal antibody (JL-8) Anti-GFP	Clontech	Cat# 632381
Goat anti-mouse IgG (H + L)-conjugate	Bio-Rad	Cat# 170-6516
**Oligonuceotides**		
*fat-6*_fw: CGCTGCTCACTATTTCGGAT	This paper	N/A
*fat-6*_rev: AAGTTGTGACCTCCCTCTCC	This paper	N/A
*fat-7*_fw: GCGCTGCTCACTATTTTGGT	This paper	N/A
*fat-7*_rev: GTGGGAATGTGTGGTGGAAA	This paper	N/A
*pmp-3*_F3: CACTCATCTCTATGACGACGTTTC	This paper	N/A
*pmp-3*_R3: CACCGTCGAGAAGCTGTAGA	This paper	N/A
αSyn_F2: ATTGCAGCAGCCACTGGCTTTG	This paper	N/A
αSyn_R2: GGCTTCAGGTTCGTAGTCTTG	This paper	N/A
YFP_fw: TGGACGGCGACGTAAAC	This paper	N/A
YFP_rev: TGGTGCAGATGAACTTCAGG	This paper	N/A
**Software**		
WF-NTP software	Nollen Lab	NA
Fiji ImageJ	NIH	RRID: SCR_003070
Zen 3.5 (Blue edition)	Carl Zeiss Microscopy GmbH	RRID: SCR_013672
GraphPad Prism 10	GraphPad Software	RRID: SCR_002798
R version 4.5.3	Posit Software	RRID: SCR_000432

### *C. elegans* strains

Standard conditions were used for maintaining *C. elegans* strains at 20 °C. Animals were synchronized by hypochlorite bleaching, hatched overnight in M9 buffer at 20 °C and subsequently cultured on NGM agar plates seeded with OP50. Worms were grown from L1 until day 5 of adulthood at 20 °C. For ageing experiments, worms were transferred at L4 stage to NGM plates containing 5-Fluoro-2’deoxyuridine (FUDR) to inhibit growth of offspring. All the strains used in this study are listed in Table EV1.

### Oil Red O staining

To measure fat accumulation, synchronized adult animals were stained with Oil Red O (ORO) (Zhang et al, 2024). Briefly, 100 worms were washed three times with M9 and allowed to settle at the bottom by gravity. The supernatant was removed, and worms were fixed with 1% paraformaldehyde (PFA), dissolved in M9 at 1:1 ratio. The samples were left to rotate for 30 min at room temperature. They were then freeze-thawed three times and washed again with M9 three times to remove PFA. The worms were then resuspended in 60% isopropanol and incubated for 15 min at room temperature. After the worms were allowed to settle at the bottom, the isopropanol was removed, 1 mL of 60% ORO working solvent was added and the worms were incubated for 4 h. The ORO was removed by washing three times with PBS. Worms were mounted on slides and imaged using stereomicroscope (Stereo DiscoveryV12; Carl Zeiss) with Axiocam 503 color camera.

For ORO quantification, worms were imaged under identical exposure settings. The anterior intestinal region was selected for analysis for each worm. Using ImageJ, the longitudinal-sectional area of the intestine was measured from brightfield images, and the mean staining intensity within this region was quantified from corresponding ORO images. Final ORO intensity values are reported as mean staining intensity normalized to intestinal area (pixels²), representing lipid density.

### Food intake measurement

OP50::mCherry bacteria were cultured overnight in LB broth. A 0.2 mL aliquot of the harvested bacteria was spread onto a 6 cm agar plate and allowed to grow overnight. Worms were subsequently transferred onto the bacterial lawn. Five minutes after transfer, worms were picked and mounted on slides for observation under an imaging microscope. Images were captured using an AxioCam HRm camera and ZEN lite software. Fluorescence intensity was quantified and analyzed using ImageJ.

### Sample preparation for lipidomics

To analyse lipid composition using mass spectrometry, YFP (OW450) and αSyn-YFP (OW40) worms were collected at day 11 of adulthood. Synchronized animals were collected in M9 buffer in 15 mL tubes and washed at least four times, by allowing the worms to settle by gravity between washes to remove bacteria. Finally, worms were transferred to 1.5 mL tubes, frozen with liquid nitrogen and stored at −80 °C until analysis.

### Lipidomics analysis

According to a previous study (Molenaars et al, 2021), around ~2500 worms were collected in a 2 mL tube. A group of mixed internal standards were used for correcting the variation in lipid extraction and analysis. A 5 mm steel bead and 1:1 (v/v) methanol:chloroform was added to each sample to 1.5 mL. Samples were homogenized using a TissueLyser II (Qiagen) for 5 min at 30 Hz and centrifuged for 10 min at 20,000× *g*. The protein concentration of the lysate was determined using a Pierce™ BCA protein assay kit (Thermo Scientific). The supernatant was transferred to a 2 mL tube and evaporated at 45 °C. The dried lipids were redissolved in 50 μL chloroform/MeOH/H2O (60:30:4.5, v/v/v) and further diluted with 150 μL isopropyl alcohol (IPA):acetonitrile (ACN):H_2_O (2:1:1, v/v/v). Lipids were separated by using an Acquity UPLC CSH column (1.7 μm, 100 × 2.1 mm) on an Acquity UPLC system (Waters, Manchester, UK). Mobile phases consisted of 10 mM ammonium formate in water (eluent A) and 10 mM ammonium formate in methanol (eluent B). Linear gradient elution was as follows: 0–5 min from 50 to 30% eluent A, 5–15 min from 30 to 10% eluent A, and 15–25 min from 10 to 0% eluent A. This was followed by isocratic elution at 0% eluent A over the next 15 min. A conditioning cycle of 5 min with the initial proportions of eluents A and B was performed prior to the next analysis. The column temperature was set at 80 °C, and the flow rate was 0.5 mL/min. In total, 2 μL of sample was injected randomly in positive and negative modes, respectively. Mass spectrometry detection was performed using a Synapt G2-Si high-resolution QTOF mass spectrometer (Waters, Manchester, UK). Nitrogen and argon were used as desolvation and collision gas, respectively. Data were acquired over the m/z range from 50 to 2000 Da in continuum and enhanced resolution modes, at an acquisition rate of 1 spectrum/0.3 s. The source temperature was set at 120 °C, the desolvation temperature at 600 °C, the cone voltage at 30 V, and the capillary voltage was 0.50 kV for ESI(+) and 0.70 kV for ESI( − ). MS experiments were performed with MS^E^ mode. The LockSpray internal reference used for these experiments to allow operation of the instrument at high mass accuracy.

To reduce and analytical variation, each batch included randomization of the sequence order and measuring a quality control (QC) sample at regular intervals (every 6 samples) in both modes. The identified lipids were presented as sum of FA chains without precise structural identification. Data were filtered on the pooled QC samples that were included in each run (coefficient of variation < 30%). All detected metabolite intensities were normalized to appropriate internal standards, as well as total protein content in samples, determined using a Pierce™ BCA Protein Assay Kit.

### RNAi screening

RNAi screening experiments were performed on 6 cm NGM agar plates containing 1 mM isopropylthio-β-D-galactoside (IPTG) and 50 mg/mL ampicillin that were seeded with RNAi bacteria. RNAi plates were kept at room temperature for at least 3 days to allow the bacteria to produce dsRNA. First larval stage (L1) animals were grown on RNAi plates after hypochlorite bleaching and used for the experiments. At L4 stage after synchronization, add 1 mL FUDR (125 μg/mL) to the NGM plates to inhibit growth of offspring. The RNAi clones used were verified by sequencing the insert using L4440 primers and subsequent BLAST analysis to confirm the correct sequence. After a first round in which ~62 genes were individually knocked down and motility was determined on day 5 of adulthood. The motility was analyzed by using wide field-of-view nematode tracking platform (WF-NTP) according to our previous study (Koopman et al, 2020).

### Preparation of lipid-supplemented media

Lipid-supplemented NGM plates were prepared using modified protocols based on (Han et al, 2017) for oleic acid supplementation and (Castillo-Quan et al, 2023) for triglyceride supplementation. After autoclaving, NGM agar was cooled to 55–60 °C before NP-40S was added to a final concentration of 0.001% in both control and lipid-supplemented media. The respective lipid supplements were then added, and the medium was vigorously mixed after lipid addition and again immediately before pouring to ensure even dispersion. After seeding, plates were incubated at room temperature for 24-48 h before worm transfer to allow incorporation of the supplemented lipids into the bacterial food source.

For experiments combining oleic acid supplementation with RNAi, 6-cm NGM plates were prepared either without lipid supplementation or with 0.8 mM oleic acid (OA). Plates were seeded with *E. coli* HT115 carrying the L4440 empty vector (EV) or RNAi constructs targeting *fat-6* or *fat-7*.

For medium-chain triglyceride (MCT) and long-chain triglyceride (LCT) supplementation experiments, 9-cm NGM plates were prepared with an increased agar concentration of 25 g/L to prevent worm burrowing. Filter-sterilized MCT (glyceryl trioctanoate) or LCT (olive oil) was added to the cooled medium at final concentrations of 0.05%, 0.1%, 0.25%, 0.5%, 1%, or 2% (v/v). Plates were seeded with ×10 concentrated *E. coli* OP50, and ~1100 worms were subsequently transferred to each plate.

### Fatty acid analysis

Worms were collected from NGM plates, washed three times with deionized water, and transferred into glass screw-cap tubes after centrifugation. Fatty acids were extracted and methylated by incubation with 1 mL of 2.5% H_2_SO_2_ in methanol at 80 °C for 1 h. Following the addition of n-hexane (100 μL) and water (1.5 mL), the upper organic phase containing fatty acid methyl esters (FAMEs) was collected after centrifugation and subjected to gas chromatography analysis.

### Imaging and quantification of α-synuclein inclusions

Day-5 adult αSyn(WT)-YFP (OW40) worms were immobilized on 2% agarose pads using 50 mM levamisole (Sigma-Aldrich, Cat# L9756), and z-stack images of the head region were acquired using a Zeiss Axio Imager.M2 microscope with a ×40 oil-immersion objective. Images were randomized, and image analysis was performed with experimental conditions blinded.

Maximum-intensity projections were generated using Fiji (version 2.0.0-rc-69/1.54 f), and background fluorescence was removed using rolling-ball subtraction with a radius of 25 pixels. Images were thresholded using a dynamic lower threshold corresponding to the brightest 1.2% of pixels and a fixed upper threshold set to the maximum intensity value. α-Synuclein inclusions larger than 0.5 µm² were quantified from the anterior tip of the head to the end of the terminal pharyngeal bulb using the Analyze Particles function. Overlapping inclusions were manually corrected. At least 20 worms per condition were analyzed in each of three independent biological experiments.

### BODIPY staining

Muscle lipid droplet was visualized using BODIPY 558/568 C12 (4,4-Difluoro-5-(2-Thienyl)-4-Bora-3a,4a-Diaza-s-Indacene-3-Dodecanoic Acid, Molecular Probes) staining (Mehlem et al, 2013). BODIPY 558/568 C12 was dissolved in DMSO to produce a 5 mM stock solution. The stock solution was then freshly diluted in M9 buffer (1:1000) and spread on the surface of NGM agar plates seeded with OP50 to final concentration ~125 nM and dried in the dark. The animals were then transferred to the BODIPY plates, incubated at 20 °C, and subjected to fluorescence microscopy.

### Western blot

Worms (approximately 20 per sample) were transferred directly into Laemmli buffer (5x) and boiled at 95 °C for 10 min. The denatured samples were then loaded onto a 12% Tris-Glycine acrylamide gel and electrophoresed. Proteins were transferred onto a 0.2 mm nitrocellulose membrane. Membranes were blocked with 5% milk in PBS-T (0.1%) for 1 h. For protein detection, primary antibodies against synuclein (1:1000 dilution) and GFP (1:1000 dilution) were applied in 5% milk, and anti-tubulin antibody (1:10,000 dilution) in fresh 5% milk, and incubated overnight at 4 °C. Antibody binding was visualized using Amersham ECL Prime Western Blotting Detection Reagent and imaged with the ImageQuant LAS400 Imaging unit (GE Healthcare).

### RNA isolation and quantitative PCR

To assess gene expression levels after RNAi knockdown, total RNA was extracted from *C. elegans* using TRIzol reagent according to the manufacturer’s instructions. RNA quality and concentration were determined using a NanoDrop 2000 spectrophotometer (Thermo Scientific). cDNA was synthesized from 1 µg of total RNA using the RevertAid H Minus First Strand cDNA Synthesis Kit with random hexamer primers. Quantitative real-time PCR (qRT-PCR) was performed using the Roche LightCycler 480 Instrument II (Roche Diagnostics) with 2 µL of tenfold diluted cDNA as template and SYBR Green dye for detection. The PCR cycling conditions were as follows: initial denaturation at 95 °C for 10 min; 40 cycles of 95 °C for 15 s, 60 °C for 30 s, and 72 °C for 15 s; followed by a melting curve analysis with 95 °C for 5 s, 65 °C for 1 min, and a gradual increase to 97 °C. Relative transcript levels were calculated using a standard curve from pooled cDNA samples. Gene expression was normalized to the endogenous reference gene *pmp-3*.

### Microscopy analysis

Worms were anaesthetized using 50 mM levamisole and high magnification (40x objective) z-stack images of the head region were obtained by using a Zeiss Axio imager 2 microscope or Confocal Microscopy (ZEISS LSM 900 with Airyscan). Lipid droplets were quantified manually, assisted by the Fiji analyze particle function applied to maximum intensity projections of the z-stacks. At least 10 worms were quantified per time point per condition. Each experiment was performed in triplicate, unless stated otherwise.

### Processing and segmentation of mitochondrial images

Airyscan 3D processing was applied to the acquired z-stacks using Zeiss ZEN Blue 3.5. The mRFP channel was isolated using Fiji, and maximum-intensity projections were generated, converted to 8-bit, and exported as TIFF files. Mitochondria were segmented using the 2D Threshold function of the Mitochondria Analyzer plugin (https://github.com/AhsenChaudhry/Mitochondria-Analyzer). Preprocessing included rolling-ball background subtraction with a radius of 1.25 µm, Sigma Filter Plus, local-contrast enhancement with a maximum slope of 2.4, and gamma correction with a gamma value of 0.80. Weighted Mean local thresholding was applied using a block size of 1.25 µm and a C-value of 1.5 for the YFP and αSyn(Δ)-YFP strains or 3.5 for the αSyn-YFP strain. Despeckle and Remove Outliers were enabled during post-processing.

The resulting masks were visually inspected against the source images and orthogonal projections. Images in which background fluorescence was incorrectly segmented as mitochondria were reprocessed using a higher C-value.

### Quantification of mitochondrial morphology

Mitochondrial morphology was quantified from the final masks using the 2D Analysis function of the Mitochondria Analyzer plugin. Mean aspect ratio, defined as the ratio of the major axis length to the minor axis length of each mitochondrial object (AR = major axis/minor axis), and mean mitochondrial area were used as the primary readouts of mitochondrial elongation and mitochondrial size, respectively. Higher mean aspect ratio values indicate more elongated, tubular mitochondria, whereas values approaching 1 reflect more spherical or fragmented mitochondrial structures. All parameters were interpreted as measures of 2D projected mitochondrial morphology, and individual worms were treated as the experimental units for statistical analysis.

### Mitochondrial respiration measurement

Oxygen consumption was assessed using a Bioscience Seahorse XF96 Flux Analyser (or XFe96) as previously described (Koopman et al, 2016). Synchronized 5-day-old worms were collected in M9 buffer and washed several times to remove bacteria and larvae. Each experiment was repeated three times.

### ATP measurements

To determine ATP content, 1000 synchronized day-5 adult worms were collected from plates supplemented with oils, plates seeded with RNAi bacteria, or control plates seeded only with *E. coli* OP50, as described before. Worms were collected in M9 buffer and washed three times. Three independent biological replicates were analyzed for each condition. After removal of the supernatant, worm pellets were snap-frozen in liquid nitrogen and stored at −80 °C.

Frozen pellets were resuspended in 250 µL of ice-cold M9 buffer, snap-frozen in liquid nitrogen, and heated at 99 °C for 15 min while shaking at 300 rpm. Samples were subsequently incubated on ice for 5 min. To maximize protein extraction, samples were subjected to three rounds of sonication, each consisting of five cycles of 15 s sonication followed by 30 s rest. Samples were then centrifuged at 14,800×*g* for 10 min at 4 °C.

A 50 µL aliquot of the supernatant was collected and diluted 1:100. Immediately before measurement, 50 µL of the diluted sample was mixed with 50 µL of luciferase reagent, and luminescence was measured using a GloMax®-Multi+ Detection System (Promega). ATP concentrations were calculated from a standard curve using the ATP Bioluminescence Assay Kit CLS II (Roche) and normalized to protein concentrations.

### Meta-analysis of TAG levels in patients

This systematic review and meta-analysis were conducted in accordance with the Preferred Reporting Items for Systematic Reviews and Meta-Analyses (PRISMA) guidelines. To identify randomized controlled trials (RCTs) examining serum TAG levels in PD patients, we systematically searched PubMed, Embase, Web of Science, the Cochrane Library, and previous meta-analyses for relevant articles published up to November 25, 2022. The search strategy employed the following terms: [(((triacylglycerol and parkinson disease) OR (TAG and parkinson disease)) OR (TG and parkinson disease) AND (clinicaltrial[Filter] OR meta-analysis[Filter] OR randomizedcontrolledtrial[Filter] OR review[Filter] OR systematicreview[Filter])) OR (lipid and parkinson disease)]. Filters were applied to include only clinical trials, randomized controlled trials, and English-language publications, with no restrictions on population or publication date. Additionally, we manually screened reference lists from included studies and prior reviews to identify further relevant trials.

Following the retrieval process, studies were screened based on titles, abstracts, and full texts for eligibility. For dichotomous outcomes, we calculated pooled effect estimates using relative risk with 95% confidence intervals (CIs), applying the Mantel-Haenszel method, which is particularly suitable for small-scale studies. For continuous outcomes, we extracted either the mean change from baseline or post-intervention values along with standard deviations. Weighted mean differences were used when measurements were consistent, whereas standardized mean differences were employed for heterogeneous measures (e.g., differing scales or units). A random-effects model (DerSimonian–Laird method, based on the inverse-variance approach) was utilized to account for both within-study and between-study variability. Mean differences and SDs between baseline and endpoint were extracted for each trial. If these were not directly reported, the mean difference was calculated by subtracting baseline values from endpoint values and derived SDs from standard errors, 95% CIs, medians, or interquartile ranges where necessary. All statistical analyses were performed using Review Manager 5.4.

### Statistical analysis

All experiments were replicated three times. Microscopy images were selected from random samples to avoid any artefacts and improve the robustness of the data. The investigators were not blinded to the experimental conditions during data collection and analysis. Statistical analyses were done in GraphPad Prism 9 (10.1.2). Based on the distribution of the data the appropriate test was selected (parametric or non-parametric). Principal component analysis (PCA) was performed using the FactoMineR package. For group comparisons, an unpaired two-tailed *t* test or one-way ANOVA was conducted, followed by Tukey’s multiple comparison test. Significance values are indicated in the figures or figure legends. All data were visualized with GraphPad Prism 9 (10.1.2) or R (3.6.0).

## Supplementary information

Table EV1

Peer Review File

Source data Fig. 1

Source data Fig. 2

Source data Fig. 3

Source data Fig. 4

Source data Fig. 5

Source data Fig. 6

Source data Fig. 7

Figure EV1 Source Data

Figure EV2 Source Data

Figure EV3 Source Data

Figure EV4 Source Data

Figure EV6 Source Data

Figure EV7 Source Data

Figure EV8 Source Data

Figure EV9 Source Data

Expanded View Figures

## Data Availability

All raw data are deposited in Figshare and can be accessed through the following link: https://figshare.com/s/2ead9e6fbd84b007e3c3. The code used in data analysis in this manuscript has been posted publicly on GitHub: https://github.com/tzhang922/LipidSyn/tree/main/R_code. The source data of this paper are collected in the following database record: biostudies:S-SCDT-10_1038-S44319-026-00954-7.
