# Supplementary material for: Condensate-driven triglyceride reduction links α-synuclein to mitochondrial dysfunction
Source: EMBO Rep. 2026 Sep 29;27(21):6277–304. doi: 10.1038/s44319-026-00954-7 (PMC13743766; doi:10.1038/s44319-026-00954-7)
Supplement: Supplementary file 1 [file 44319_2026_954_MOESM18_ESM.pdf]

Expanded View Figures

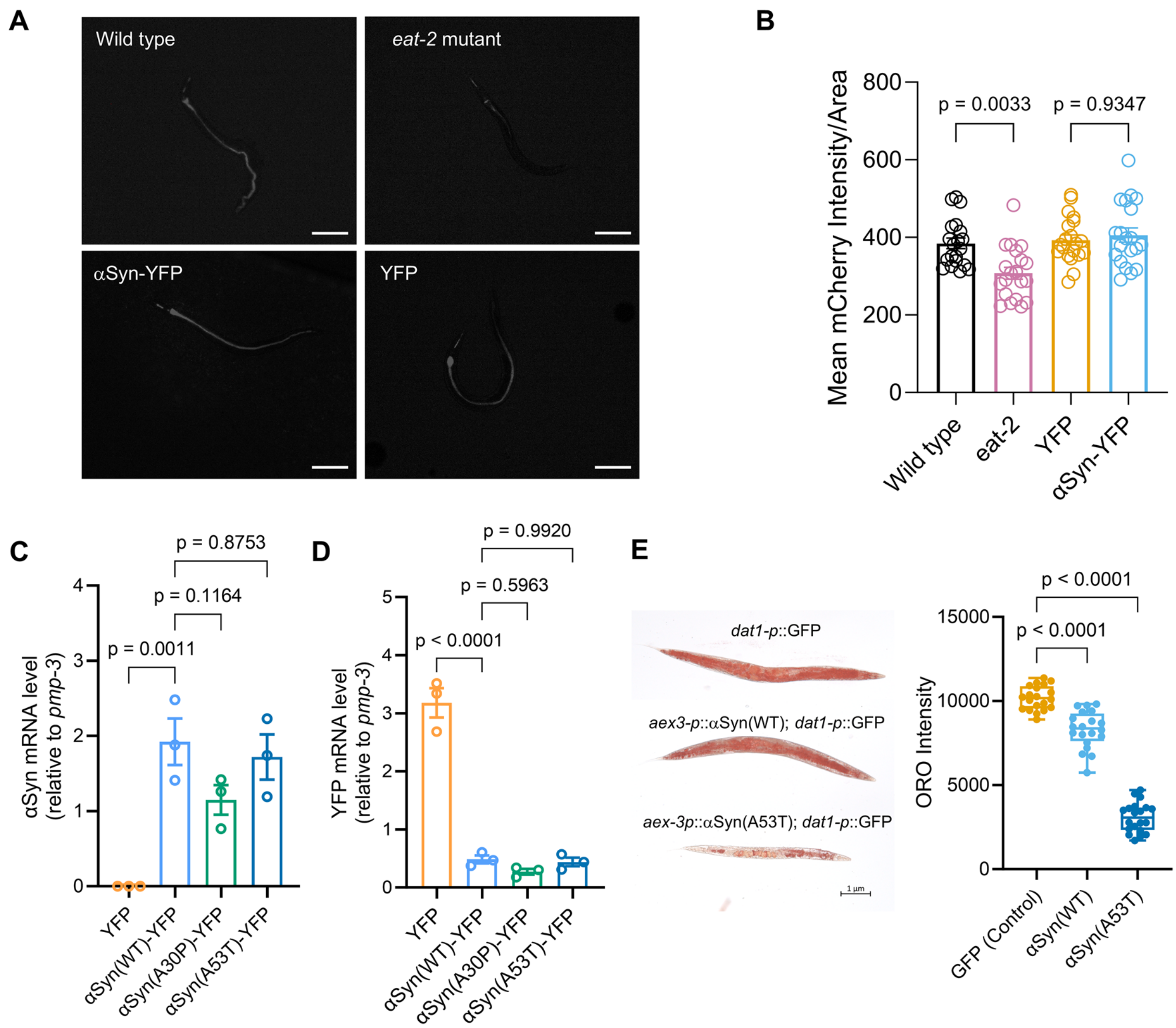

Figure EV1. Comparable food intake and αSyn/YFP transcript levels across strains, with neuronal αSyn expression associated with reduced TAG content.

(**A**) Representative images of intestinal fluorescence in αSyn-YFP and YFP control worms fed mCherry-expressing *E. coli* OP50. Scale bar, 20 µm. (**B**) Quantification of intestinal OP50::mCherry fluorescence intensity shows comparable bacterial intake between αSyn-YFP and YFP worms ($n = 20$ animals per group; one-way ANOVA, error bars represent s.e.m.). (**C**) αSyn mRNA levels were comparable among the indicated αSyn-expressing strains (one-way ANOVA, error bars represent s.e.m.). (**D**) YFP mRNA levels were likewise comparable among the indicated αSyn-expressing strains (one-way ANOVA, error bars represent s.e.m.). (**E**) Pan-neuronal expression of αSyn(WT) or αSyn(A53T), driven by the *dat-1* promoter, reduced TAG levels relative to *dat-1p*::GFP controls at day 5 of adulthood. Box plots show the median, IQR, and min–max whiskers ($n = 20$ animals; one-way ANOVA). (**A**, **E**) One representative image is shown in each condition. All experiments were performed in triplicate. Source data are available online for this figure.

© The Author(s)

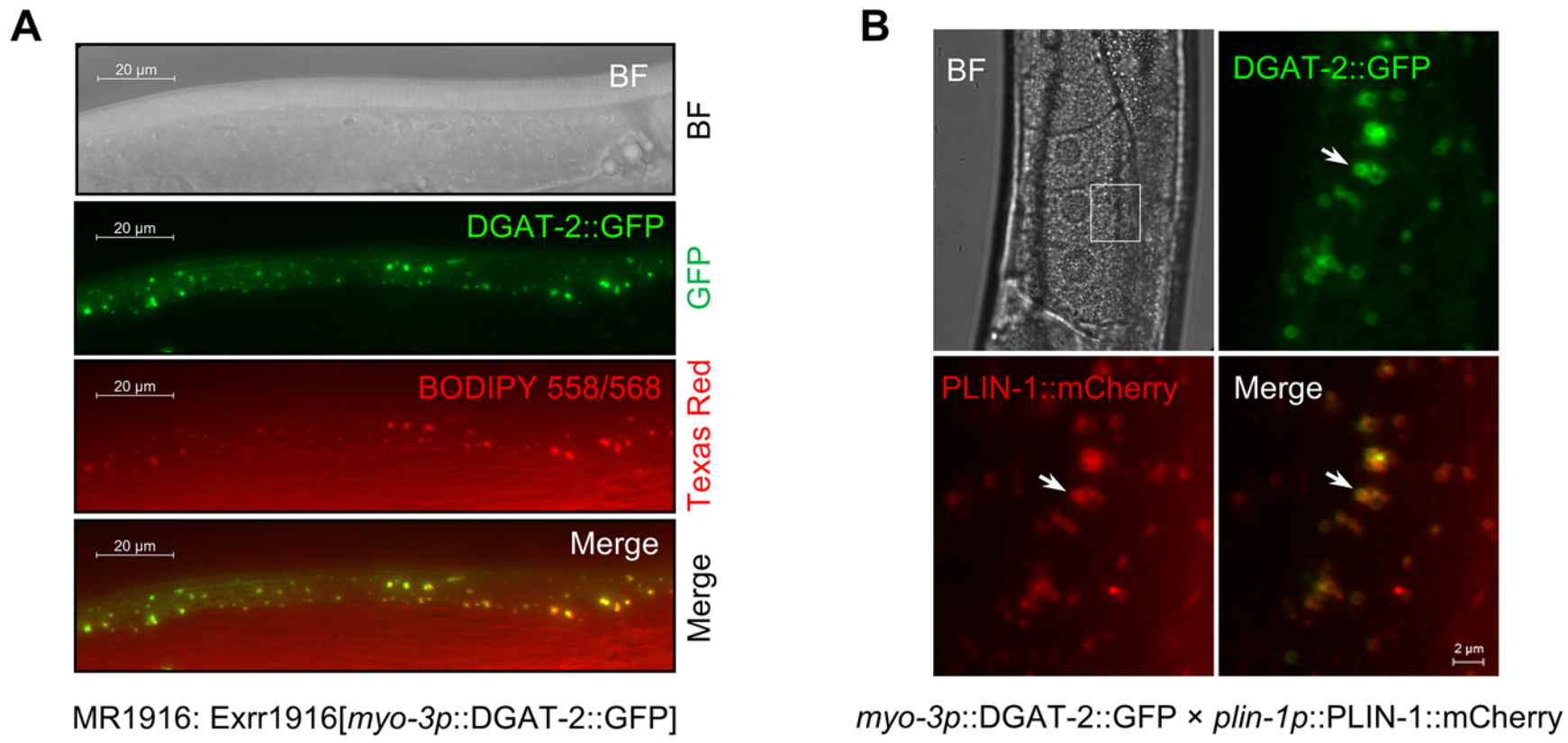

Figure EV2. BODIPY staining identifies muscle LDs and confirms PLIN-1 localization to muscle-specific LDs.

(**A**) Muscle-specific lipid droplets (LDs), visualized by BODIPY 558/568 C12 staining, colocalize with DGAT-2::GFP expressed under the *myo-3* promoter in strain MR1916, confirming the identity of BODIPY-positive structures as LDs. (**B**) Muscle-specific co-expression of DGAT-2::GFP (green) and PLIN-1::mCherry (red) demonstrates localization of PLIN-1 to muscle LDs. BF brightfield. Source data are available online for this figure.

 © The Author(s)

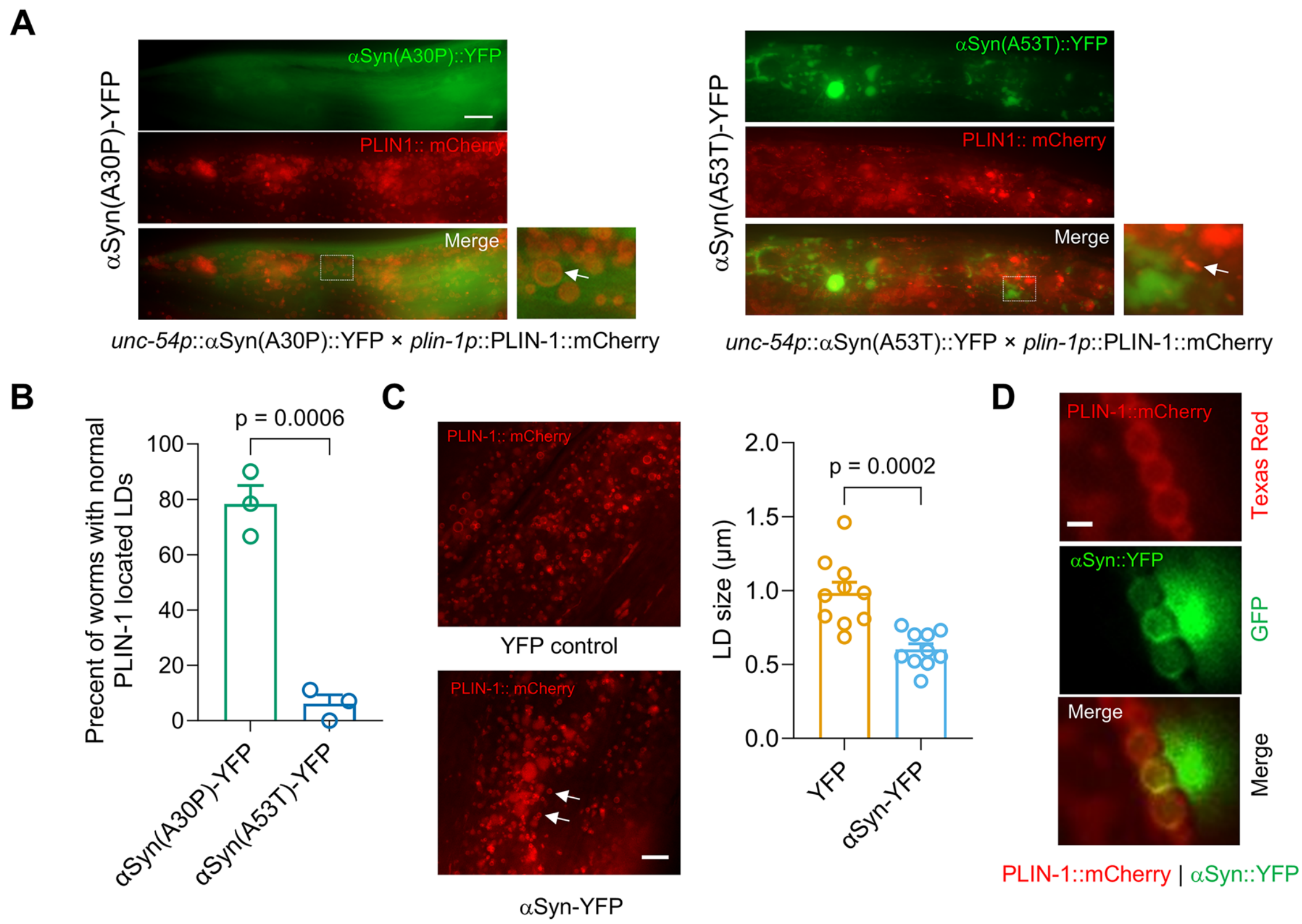

Figure EV3. αSyn-YFP expression and condensation disrupts PLIN-1 localization and LD organization.

(**A**) Compared with αSyn(A30P)-YFP, αSyn(A53T)-YFP expression is associated with abnormal PLIN-1 distribution and the appearance of filamentous PLIN-1 structures (white arrows). Insets show magnified views of the indicated regions (white dashed boxes). Scale bar, 10 µm. (**B**) αSyn(A53T) significantly reduces the proportion of worms displaying normal PLIN-1-coated lipid droplets (LDs) at day 5 of adulthood (percentages calculated for three biological replicates; $n = 15$ animals per replicate; unpaired *t* test, error bars represent s.e.m.). (**C**) αSyn-YFP worms exhibit significantly smaller LDs than YFP controls at day 11 of adulthood ($n = 10$ animals; unpaired *t* test, error bars represent s.e.m.). Scale bar, 5 µm. (**D**) Co-localization between αSyn::YFP and PLIN-1::mCherry is observed at day 1 of adulthood. Scale bar, 1 µm. Source data are available online for this figure.

© The Author(s)

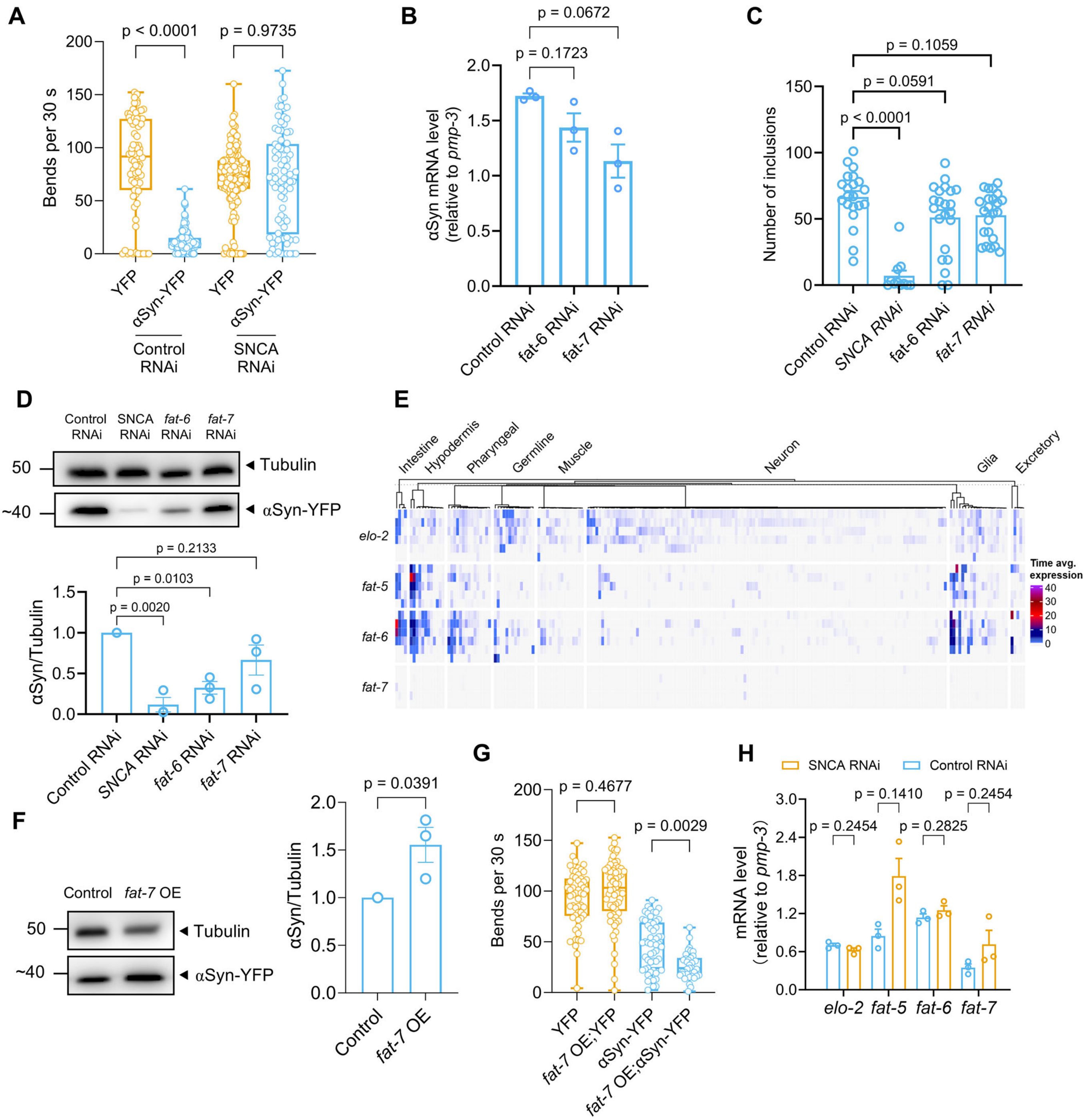

Figure EV4. Differential effects of *fat-6* and *fat-7* modulation on αSyn toxicity.

(**A**) αSyn-YFP worms exhibit significantly reduced body-bend frequency compared with YFP controls at day 5 of adulthood. Box plots show the median, IQR, and min–max whiskers ($n = 50$–100 animals per condition; one-way ANOVA). (**B**) qPCR analysis shows that *fat-6* or *fat-7* RNAi does not significantly alter *SNCA* mRNA levels in day-5 adult αSyn-YFP worms (one-way ANOVA, error bars represent s.e.m.). (**C**) Quantification of αSyn inclusions shows no significant change following *fat-6* or *fat-7* RNAi in day-5 adult αSyn-YFP worms (one-way ANOVA, error bars represent s.e.m.). (**D**) Western blot analysis shows that *fat-6* RNAi reduces αSyn protein abundance in day-5 adult αSyn-YFP worms (one-way ANOVA, error bars represent s.e.m.). (**E**) Tissue-resolved expression analysis in wild-type worms shows broad and relatively high *fat-6* expression across multiple tissues, whereas *fat-7* expression is low or undetectable in most examined tissues. (**F**) *fat-7* overexpression (OE) increases αSyn protein abundance in αSyn-YFP worms (unpaired *t* test, error bars represent s.e.m.). (**G**) *fat-7* OE exacerbates the motility defect of αSyn-YFP worms. Box plots show the median, IQR, and min–max whiskers ($n = 50$–100 animals per condition; one-way ANOVA). (**H**) Expression levels of *fat-6* and *fat-7* are not significantly altered by *SNCA* RNAi (one-way ANOVA, error bars represent s.e.m.). All experiments were performed in triplicate. Source data are available online for this figure.

 © The Author(s)

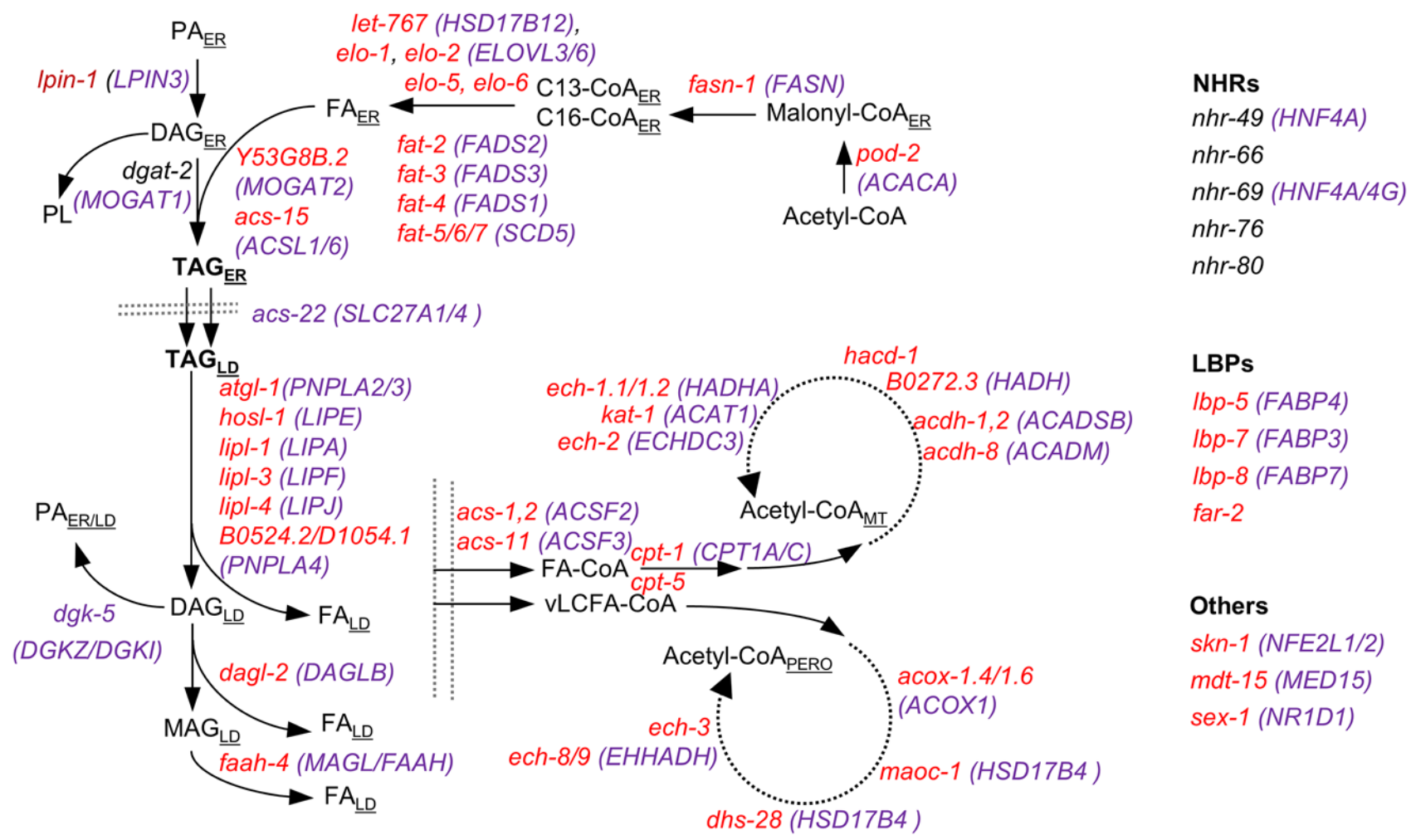

Figure EV5. The lipid metabolism pathway in *C. elegans*.

Human homologs of worm genes (red) are shown in purple.

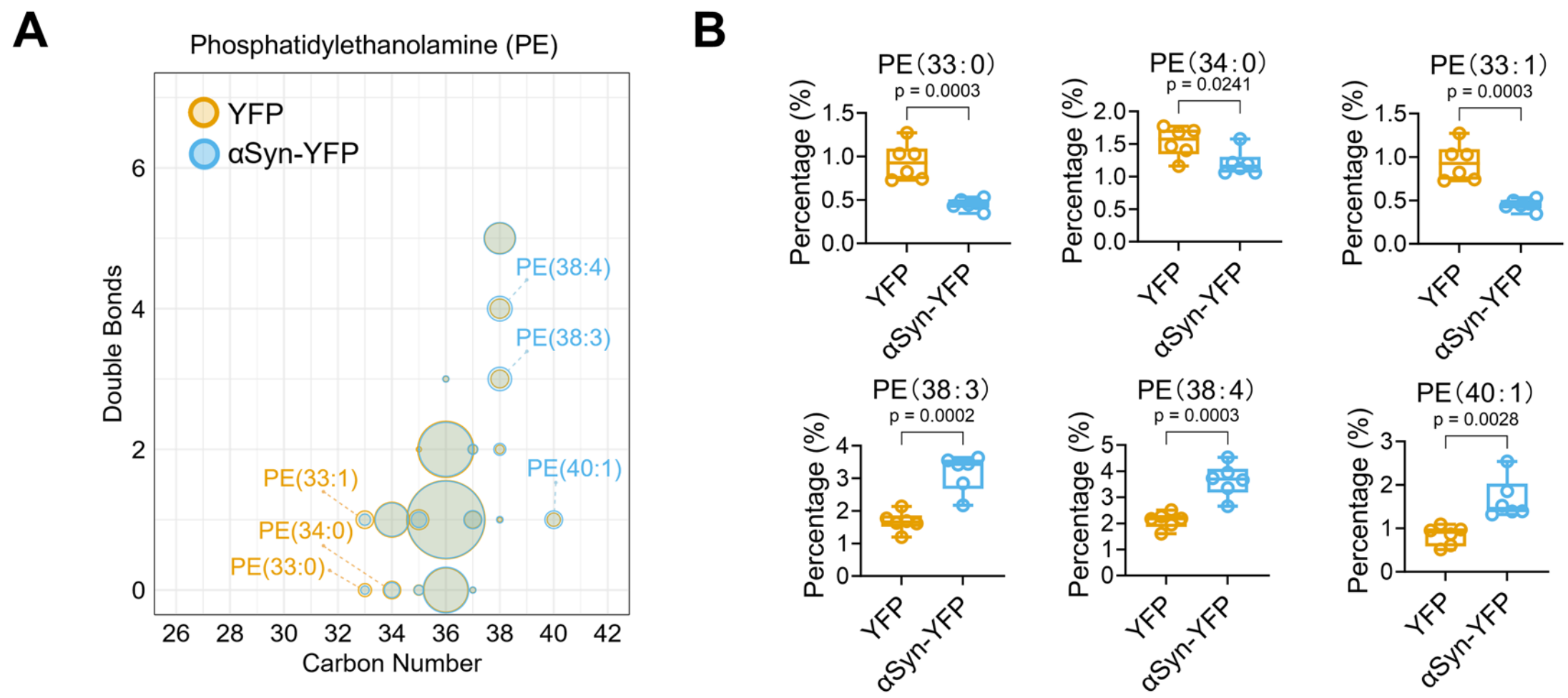

Figure EV6. αSyn proportionally increases unsaturation of fatty acyl chains in phosphatidylethanolamine.

(**A**) Lipidomics data demonstrates proportionally elevated unsaturation index (blue) in phosphatidylethanolamine (PE) species from αSyn-YFP versus YFP worms. (**B**) Percentage of six predominant PE molecular species shows higher unsaturated composition in αSyn-YFP worms compared to YFP controls. Box plots show the median, IQR, and min–max whiskers. Each lipidomics sample contained approximately 3000 worms collected from six replicate plates. Source data are available online for this figure.

 © The Author(s)

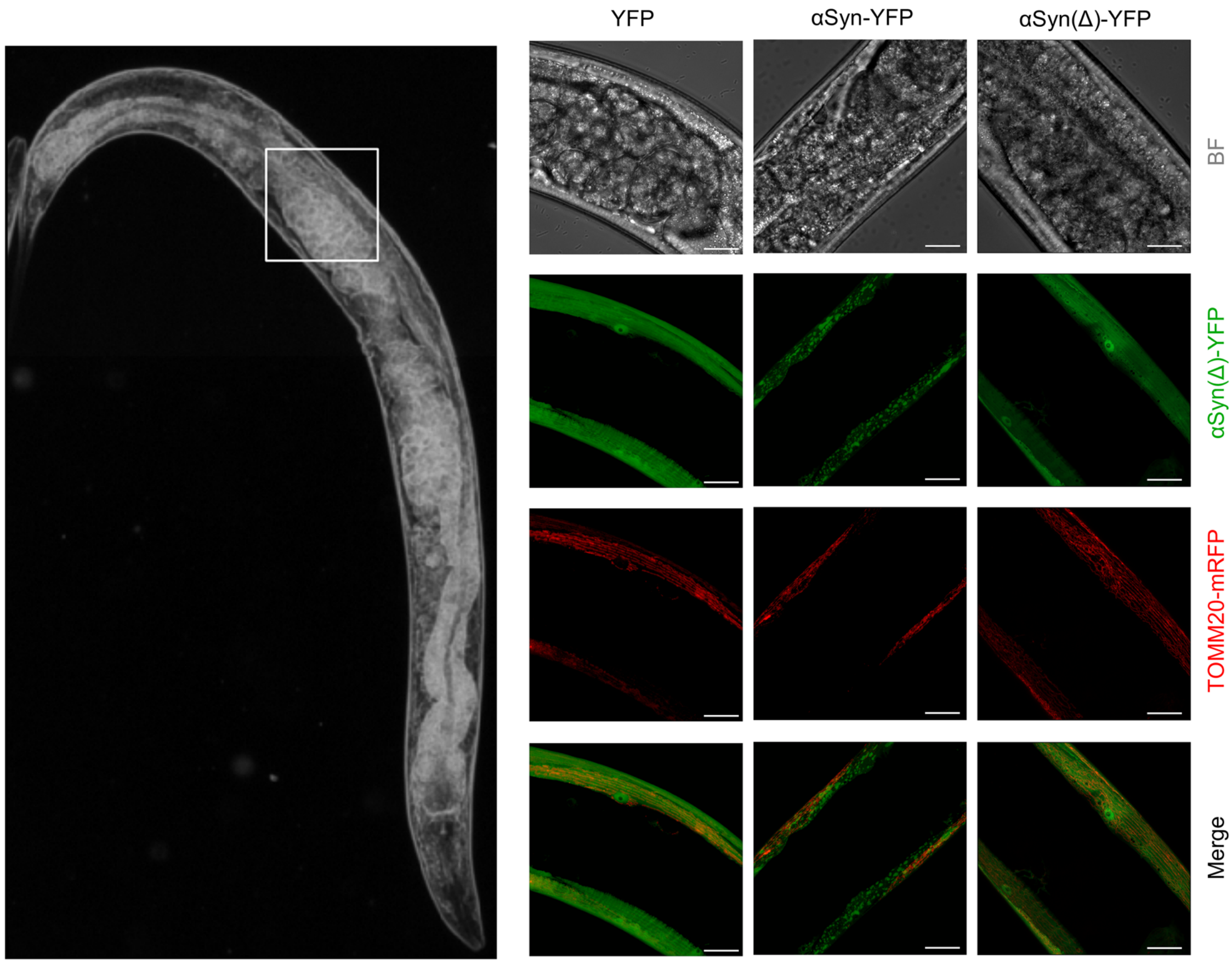

Figure EV7. Expression of αSyn(WT)-YFP, but not αSyn(Δ)-YFP, is associated with increased mitochondrial fragmentation.

Overview images of the regions used for mitochondrial morphology analysis. Scale bar, 20 µm. Images correspond to the wider-field views of those shown in Fig. 6D. Source data are available online for this figure.

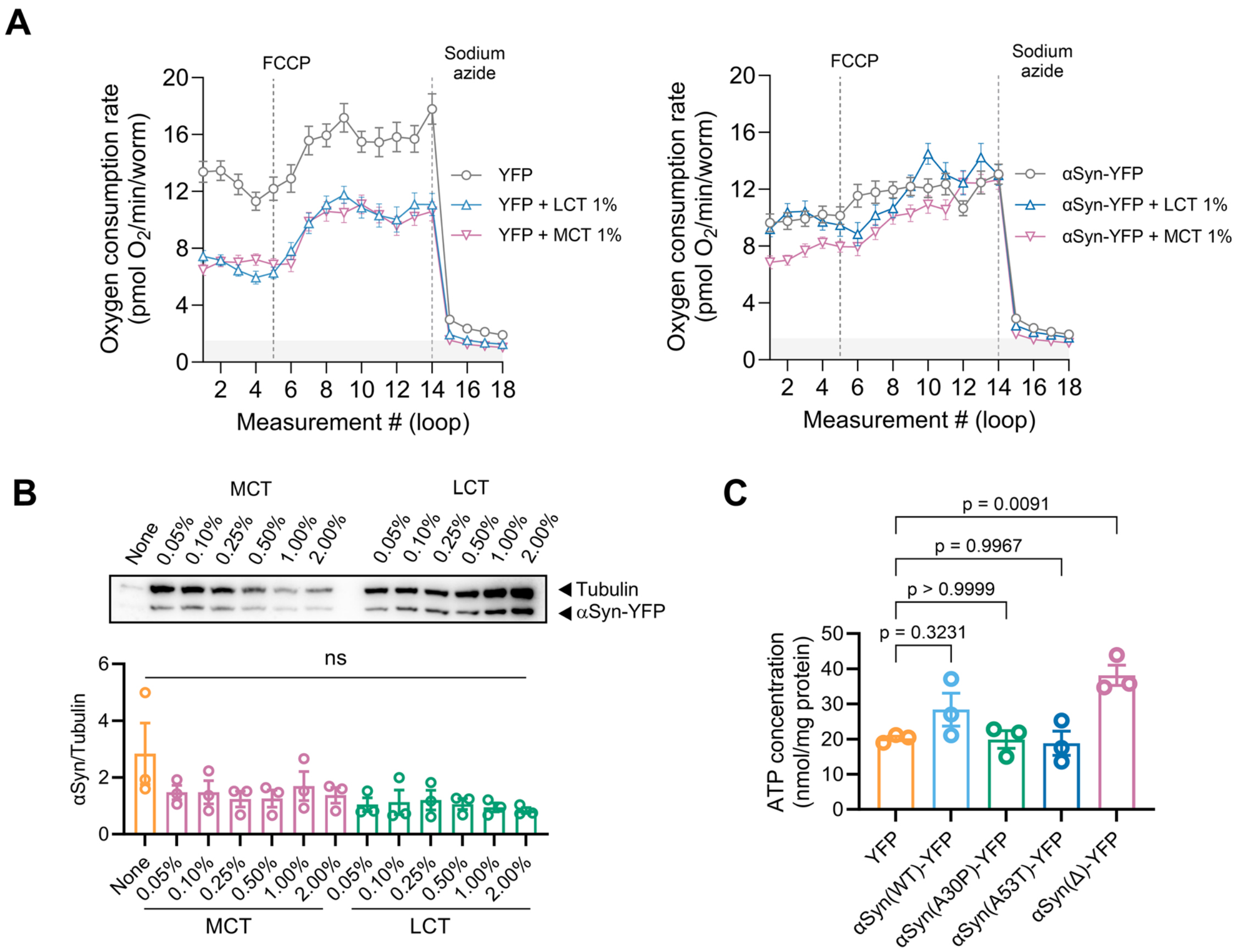

Figure EV8. Oxygen consumption rate (OCR) measurements show that αSyn-YFP worms respond selectively to MCT supplementation, but not LCT supplementation.

(**A**) Oxygen consumption rate (OCR) measurements show that αSyn-YFP worms respond selectively to MCT supplementation, but not LCT supplementation, whereas YFP control worms respond to both MCT and LCT supplementation ($n = 12$ wells, error bars represent s.e.m.). (**B**) MCT and LCT supplementation do not significantly alter αSyn protein abundance in αSyn-YFP worms (one-way ANOVA, error bars represent s.e.m.). (**C**) αSyn variants with intact membrane-binding capacity do not significantly alter ATP abundance compared with YFP controls, but αSyn(Δ)-YFP animals show significantly increased ATP abundance relative to YFP controls (one-way ANOVA, error bars represent s.e.m.). All experiments were performed in triplicate. Source data are available online for this figure.

 © The Author(s)

A

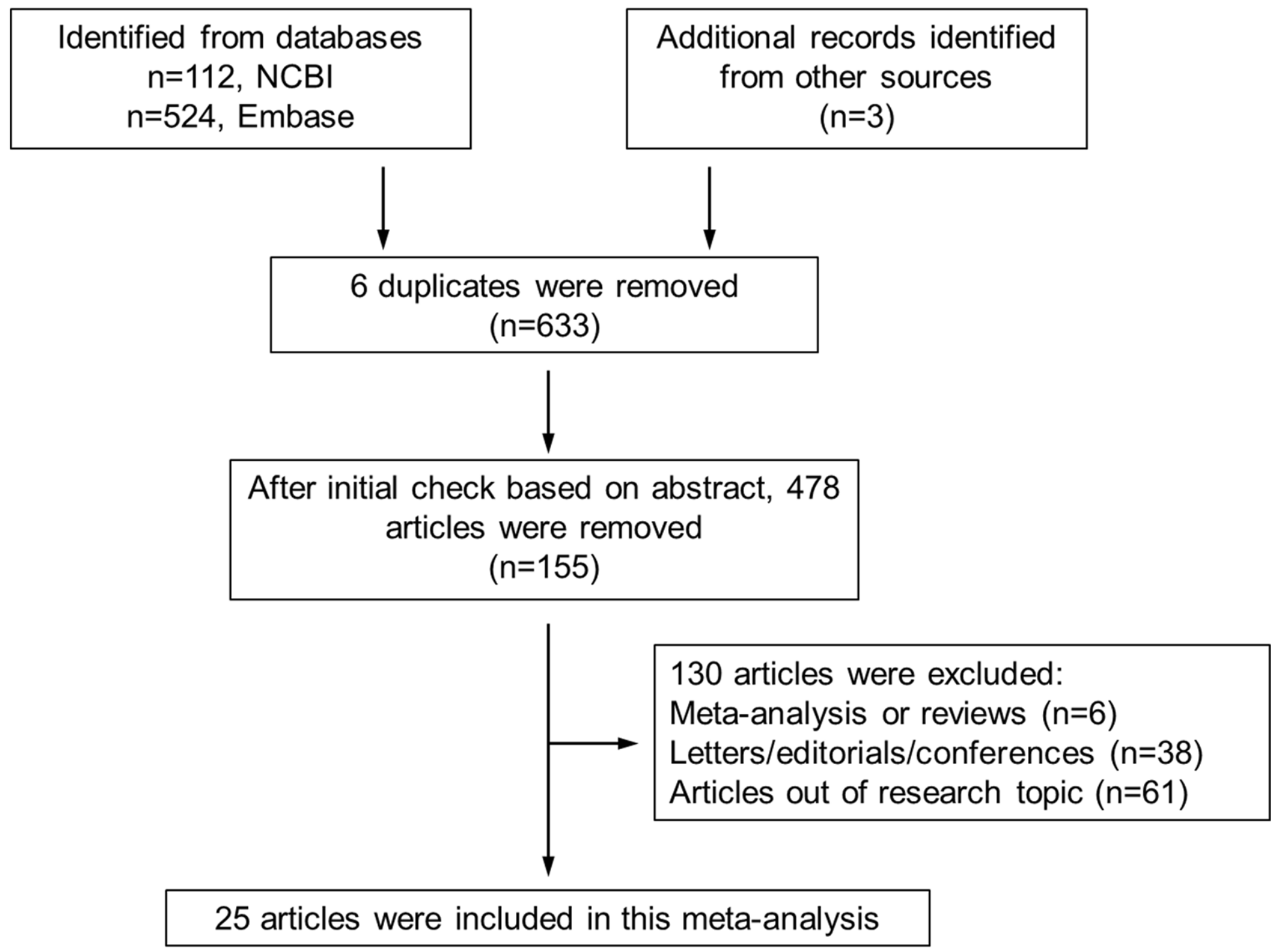

B

	PD Patients			Healthy Controls				Mean Difference
**Study or Subgroup**	**Mean**	**SD**	**Total**	**Mean**	**SD**	**Total**	**Weight**	**IV, Fixed, 95% CI**
Bai 2021	70.86	52.26	41	110.71	83.26	41	0.2%	-39.85 [-69.59, -9.76]
Brodacki 2011	113	6	10	124	2	10	14.3%	-11.00 [-14.92, -7.08]
Cereda 2012	102.7	38.1	80	128.3	60.8	80	0.9%	-26.20 [-41.92, -10.48]
Chang 2022	112.63	64.34	71	103.5	62.96	60	0.5%	9.28 [-12.58, 31.14]
Choe 2021	114.9	56.7	294	110.9	62.1	588	3.3%	4.00 [-4.20, 12.20]
Deng 2022	103.64	44.29	207	123.51	100.17	102	0.5%	-19.87 [-40.22, 0.48]
Dong 2017	110.71	67.31	75	128.43	69.97	62	0.4%	-17.72 [-40.86, 5.42]
Fukui 2016	108.3	51.5	180	116.3	63.2	219	1.7%	-8.00 [-19.25, 3.25]
Guo 2014	106.28	70.86	555	132.88	70.86	555	3.2%	-26.58 [-34.92, -18.24]
Häglin 2020	133.74	71.74	75	147.91	65.54	24	0.2%	-14.17 [-45.01, 16.67]
Ikeda 2011	116.56	56.19	120	113.2	49.58	120	1.2%	2.98 [-10.43, 16.39]
Jahed 2022	133.3	79.3	31	131.2	58.6	31	0.2%	2.10 [-26.31, 30.51]
Kirbas 2014	134.5	52.1	52	128.9	46.6	40	0.5%	5.60 [-14.63, 25.83]
Kurup 2003	78.72	8.12	15	87.38	7.33	15	7.2%	-8.66 [-14.20, -3.12]
Liu 2021	109.53	52.62	101	143.48	72.65	97	0.7%	-33.95 [-51.68, -16.22]
Macias-Garcia 2021	103.03	57.68	300	115.59	70.14	420	2.5%	-12.56 [-21.92, -3.20]
Sáksjärvi 2015	115.14	80.8	89	132.88	80.8	6522	0.8%	-17.72 [-34.62, -0.82]
Saedi 2021	111.92	8.75	75	121.64	9.97	75	24.4%	-9.72 [-12.72, -6.72]
Scigliano 2006	134	64.1	100	151	64.1	322	1.1%	-17.00 [-31.38, -2.62]
Shin 2020	123.2	67.5	38	135.4	59.2	33	0.3%	-12.20 [-41.67, 17.27]
Vikdahl 2015	117.8	42.51	84	134.63	74.4	336	1.5%	-16.83 [-28.91, -4.75]
Wu 2021	82.07	40.4	58	85.91	28.26	60	1.4%	-3.84 [-16.46, 8.78]
Ya 2017	121.34	8.86	116	131.08	10.08	100	31.7%	-9.74 [-12.37, -7.11]
Yang 2021	107.17	60.23	100	125.77	62.88	100	0.8%	-18.60 [-35.67, -1.53]
Zhang 2017	108	51	91	123.4	66.6	70	0.6%	-15.40 [-34.19, 3.39]
**Total (95% CI)**			**2958**			**10112**	**100.0%**	**-10.37 [-11.86, -8.89]**

Heterogeneity: Chi²=57.89, df=24 (P=0.0001); I²=59%

Test for overall effect: Z=13.71 (P<0.00001)

© The Author(s)

Figure EV9. Reduced serum TAG levels in patients with Parkinson's disease based on a systematic meta-analysis.

(**A**) Flow diagram of study selection process for the meta-analysis, showing identification, screening, eligibility assessment, and final inclusion of studies. (**B**) Forest plot of pooled results demonstrating significantly lower serum TAG levels in PD patients ($n = 2958$) compared to healthy controls ($n = 10,112$). The standardized mean difference with 95% confidence intervals is shown for each study and overall effect size (random-effects model). Source data are available online for this figure.

© The Author(s)
