# Supplementary material for: Condensate-driven triglyceride reduction links α-synuclein to mitochondrial dysfunction
Source: EMBO Rep. 2026 Sep 29;27(21):6277–304. doi: 10.1038/s44319-026-00954-7 (PMC13743766; doi:10.1038/s44319-026-00954-7)
Supplement: Supplementary file 2 [file 44319_2026_954_MOESM2_ESM.pdf]

EMBO reports

Condensate-Driven Triglyceride Reduction Links α-Synuclein to Mitochondrial Dysfunction

Tao Zhang, Alejandro Herron-Bedoya, Maria Eugenia Goya, Jorien van der Weerd, Dikaia Tsagkari, Suzanne Couzijn, Lale Güngördü, Anna Ainslie, Renée Seinstra, M. Rebecca Fokkema, Ming Chang, Nektarios Tavernarakis, Folkert Kuipers, and Ellen Nollen

Corresponding author(s): Ellen Nollen (e.a.a.nollen@umcg.nl), Tao Zhang (t.zhang@mail.hzau.edu.cn)

Review Timeline:

Submission Date:	12th Dec 25
Editorial Decision:	29th Jan 26
Revision Received:	14th Aug 26
Editorial Decision:	31st Aug 26
Revision Received:	11th Sep 26
Accepted:	17th Sep 26

Editor: Esther Schnapp

Transaction Report:

(Note: With the exception of the correction of typographical or spelling errors that could be a source of ambiguity, letters and reports are not edited. Depending on transfer agreements, referee reports obtained elsewhere may or may not be included in this compilation. Referee reports are anonymous unless the Referee chooses to sign their reports.)

1st Editorial Decision **29th Jan 2026**

Dear Prof. Nollen,

Thank you for the submission of your manuscript to EMBO reports. We have now received the full set of referee reports that is pasted below.

As you will see, the referees acknowledge that the findings are potentially interesting. However, they also raise some concerns and have several suggestions for how the study could be improved. I think especially referee 3's report is very good and all comments should be addressed. All other referee comments can be addressed in the ms text but naturally, if you have any data that addresses these points, please do add them to the ms. Please let me know if you have any comments or questions regarding the revisions and we can discuss them further, also in a video chat, if you like.

I would thus like to invite you to revise your manuscript with the understanding that the referee concerns must be fully addressed and their suggestions taken on board. Please address all referee concerns in a complete point-by-point response. Acceptance of the manuscript will depend on a positive outcome of a second round of review. It is EMBO reports policy to allow a single round of major revision only and acceptance or rejection of the manuscript will therefore depend on the completeness of your responses included in the next, final version of the manuscript.

We realize that it is difficult to revise to a specific deadline. In the interest of protecting the conceptual advance provided by the work, we recommend a revision within 3 months (1st May 2026). Please discuss the revision progress ahead of this time with the editor if you require more time to complete the revisions.

IMPORTANT NOTE: we perform an initial quality control of all revised manuscripts before re-review. Your manuscript will FAIL this control and the handling will be DELAYED if the following APPLIES:
1) A data availability section providing access to data deposited in public databases is missing. If you have not deposited any data, please add a sentence to the data availability section that explains that.
2) Your manuscript contains statistics and error bars based on n=2. Please use scatter blots in these cases. No statistics should be calculated if n=2.

When submitting your revised manuscript, please review the submission guidelines (https://link.springer.com/journal/44319/submission-guidelines#cms-Revised-submissions) and carefully review the instructions that follow below. Failure to include requested items will delay the evaluation of your revision.

1) a .docx formatted version of the manuscript text (including legends for main figures, EV figures and tables). Please make sure that the changes are highlighted to be clearly visible.

2) individual production quality figure files as .eps, .tif, .jpg (one file per figure). See hhttps://media.springernature.com/original/springer-cms/rest/v1/content/27825798/data/v1 for more info on how to prepare your figures.

3) We replaced Supplementary Information with Expanded View (EV) Figures and Tables that are collapsible/expandable online. A maximum of 5 EV Figures can be typeset. EV Figures should be cited as 'Figure EV1, Figure EV2" etc... in the text and their respective legends should be included in the main text after the legends of regular figures.

- For the figures that you do NOT wish to display as Expanded View figures, they should be bundled together with their legends in a single PDF file called *Appendix*, which should start with a short Table of Content. Appendix figures should be referred to in the main text as: "Appendix Figure S1, Appendix Figure S2" etc. See 'Expanded View' section of the submission guidelines.

- Additional Tables/Datasets should be labeled and referred to as Table EV1, Dataset EV1, etc. Legends have to be provided in a separate tab in case of .xls files. Alternatively, the legend can be supplied as a separate text file (README) and zipped together with the Table/Dataset file.

4) a .docx formatted letter INCLUDING the reviewers' reports and your detailed point-by-point responses to their comments. As part of the EMBO Press transparent editorial process, the point-by-point response is part of the Review Process File (RPF), which will be published alongside your paper.

5) a complete author checklist, which you can download from our author guidelines. Please insert information in the checklist that is also reflected in the manuscript. The completed author checklist will also be part of the RPF.

6) Please note that all corresponding authors are required to supply an ORCID ID for their name upon submission of a revised manuscript (<https://orcid.org/>), which can be linked to your profile in the manuscript submission system.

7) Before submitting your revision, primary datasets produced in this study need to be deposited in an appropriate public

database. Please remember to provide a reviewer password if the datasets are not yet public. The accession numbers and database should be listed in a formal "Data Availability" section placed after Methods. Please note that the Data Availability Section is restricted to new primary data that are part of this study. * Note - All links should resolve to a page where the data can be accessed. *

If your study has not produced novel datasets, please mention this fact in the Data Availability Section.

8) At EMBO Press we ask authors to provide source data for the main manuscript figures. You will receive a separate email with instructions for providing source data with your revised manuscript, including how to upload and organize the files.

9) Our journal also encourages inclusion of *data citations in the reference list* to directly cite datasets that were re-used and obtained from public databases. Data citations in the article text are distinct from normal bibliographical citations and should directly link to the database records from which the data can be accessed. In the main text, data citations are formatted as follows: "Data ref: Smith et al, 2001" or "Data ref: NCBI Sequence Read Archive PRJNA342805, 2017". In the Reference list, data citations must be labeled with "[DATASET]". A data reference must provide the database name, accession number/identifiers and a resolvable link to the landing page from which the data can be accessed at the end of the reference.

10) Regarding data quantification, the following points must be specified in each figure legend:

- the name of the statistical test used to generate error bars and P values,

- the number (n) of independent experiments (please specify technical or biological replicates) underlying each data point,

- the nature of the bars and error bars (s.d., s.e.m.),

- If the data are obtained from n Program fragment delivered error ``Can't locate object method "less" via package "than" (perhaps you forgot to load "than"?) at //ejpvfs23/sites23b/embor_www/letters/embor_decision_revise_and_review.txt line 54.' 2, use scatter blots showing the individual data points.

Discussion of statistical methodology can be reported in the materials and methods section, but figure legends should contain a basic description of n, P and the test applied.

- Please also include scale bars in all microscopy images.

11) The journal requires a statement specifying whether or not authors have competing interests (defined as all potential or actual interests that could be perceived to influence the presentation or interpretation of an article). In case of competing interests, this must be specified in your disclosure statement.

12) All Materials and Methods need to be described in the main text using our 'Structured Methods' format, which is required for all research articles. According to this format, the Methods section includes a separate Reagents and Tools Table file (listing key reagents, experimental models, software and relevant equipment and including their sources and relevant identifiers) and a Methods and Protocols section describing the methods using a step-by-step protocol format. The aim is to facilitate adoption of the methodologies across labs. More information on how to adhere to this format as well as a downloadable template (.docx) for the Reagents and Tools Table can be found in our author guidelines.

We would also welcome the submission of cover suggestions, or motifs to be used by our Graphics Illustrator in designing a cover.

As part of the EMBO publication's Transparent Editorial Process, EMBO reports publishes online a Review Process File (RPF) to accompany accepted manuscripts. This File will be published in conjunction with your paper and will include the referee reports, your point-by-point response and all pertinent correspondence relating to the manuscript.

You are able to opt out of this by letting the editorial office know (emboreports@embo.org). If you do opt out, the Review Process File link will point to the following statement: "No Review Process File is available with this article, as the authors have chosen not to make the review process public in this case."

I look forward to seeing a revised form of your manuscript when it is ready.

Best regards,
Esther

Esther Schnapp, PhD
Senior Editor

Referee #1:

This study shows that Parkinson's disease-linked α-synuclein (αSyn) disrupts lipid metabolism and cellular function in C. elegans. Lipidomic analyses reveal progressive triacylglycerol (TAG) depletion and accumulation of long-chain unsaturated fatty acids (LCUFAs) upon αSyn expression. Importantly, TAG loss depends on αSyn aggregation: inclusion-forming variants (WT, A53T) induce marked TAG depletion, whereas the non-aggregating A30P variant does not. αSyn also disrupts lipid droplet architecture and mislocalizes PLIN-1, impairing lipid storage. These lipid defects are linked to mitochondrial fragmentation, reduced respiration, and motility decline. RNAi screening shows that suppressing LCUFA synthesis (fat-6/fat-7 knockdown) rescues motility, whereas restoring total TAG levels alone does not, indicating that fatty-acid composition-not TAG abundance-drives toxicity. αSyn accumulates near mitochondria, suggesting that altered TAG availability and lipid composition underlie mitochondrial dysfunction and neurotoxicity. This manuscript is well written, and can reach the level of publication after revision.

Major issues:
1. There is a mechanistic gap between condensates and TAG depletion. While the paper demonstrates that TAG depletion depends on αSyn's ability to form condensates/inclusions, the actual mechanism remains unclear. The authors note minimal colocalization between αSyn inclusions and lipid droplets at later stages (Fig. 3c, S3a, S3d), suggesting indirect effects, but they don't adequately explain how condensates cause TAG depletion. Is it through sequestration of lipid metabolic enzymes? Disruption of ER-LD contacts? Altered gene expression? The paper needs experiments to directly test these possibilities rather than relying primarily on correlative observations.

2. There is an inconsistency for fat-6 and fat-7 results. The differential effects of fat-6 versus fat-7 manipulation are confusing and inadequately addressed. Fat-6 RNAi reduces both TAG levels and αSyn expression while also decreasing worm size, which the authors attribute to "systemic effects." However, fat-7 RNAi restores TAG levels and rescues motility without affecting size. Yet fat-7 overexpression increases αSyn levels and impairs motility. These seemingly contradictory results suggest complex, potentially indirect mechanisms that deserve dedicated experiments and discussion rather than the brief speculation provided.

Referee #2:

This study employed a nematode aging model combined with untargeted lipidomics, live imaging, RNAi functional screening, and clinical big-data meta-analysis to systematically elucidate that α-synuclein aggregates drive motor deficits through a coherent axis of "decreased total TAG levels-elevated proportion of long-chain unsaturated fatty acids-mislocalization of lipid droplet structural protein PLIN-1-impaired mitochondrial respiration." The authors demonstrated the conservation of reduced serum TAG levels in Parkinson's patients across species. They proposed an intervention strategy involving dietary medium-chain fatty acids to bypass the LCUFA synthesis blockade and directly restore mitochondrial energy metabolism. This study provides a mechanistically clear, operationally simple, and highly translatable approach for the metabolic regulation therapy of synucleinopathies. Certain concerns need to be addressed before it is considered for publication.
1. The authors only tested 0.5% and 1.0% MCT, lacking a dose-response curve. Moreover, does MCT affect αSyn aggregation per se? Similarly, while fat-6 and fat-7 influence the expression level of αSyn, what would be their effects on αSyn aggregation?
2. On page 8, line 24, "dirsupt" is a spelling error, which should be corrected to "disrupt". On page 12, line 12, "subcellullar" is a spelling error, which should be corrected to "subcellular". On page 15, line 4, "quanlity" is a spelling error, which should be corrected to "quality". On page 16, line 8, "fluoresence" is a spelling error, which should be corrected to "fluorescence". On page 17, line 9, "meaurement" is a spelling error, which should be corrected to "measurement". The author is advised to review and revise the entire text accordingly.
3. The manuscript contains some incorrect figure references, for example："Indeed, motility in αSyn-YFP worms was significantly improved when fed 0.5% or 1.0% MCT compared to YFP controls, whereas feeding LCT did not improve motility (Fig 6c)." This should be changed to "fig 6e". "In addition, mitochondrial respiration measurements revealed that αSyn-YFP worms respond to MCT feeding but not LCT, while mitochondria of YFP worms respond to both MCT and LCT feeding (Fig. 6e)." This should be changed to "fig 6f".

Referee #3:

Summary: This manuscript investigates the link between aSyn toxicity and lipid metabolism in C. elegans. The authors report that aSyn reduces TAG levels and disrupts LDs, proposing that these changes lead to mitochondrial dysfunction and impaired motility. They further suggest that fat6/7 RNAi or supplementing with medium-chain triglycerides (MCT) mitigates toxicity.
While the connection between lipid metabolism and aSyn toxicity is of high interest, and the clinical correlation with PD patient serum is intriguing, the manuscript contains several logical inconsistencies and lacks critical quantitative validations. Specifically, the relationship between TAG levels, LCUFA composition, and mitochondrial bioenergetics remains contradictory. Major

revisions are required to substantiate the proposed mechanism.

Major Comments

1. The title, "Condensate-Driven Triglyceride Depletion Links...", explicitly posits that the reduction in TAG levels is the causal mechanism driving mitochondrial dysfunction. However, the data demonstrates that fat-6 RNAi rescues motility despite causing a further reduction in lipid stores (as indicated by Oil Red O staining) (Fig. 4b, c). This result creates a logical paradox: if "triglyceride depletion" were the direct driver of toxicity, fat-6 RNAi should exacerbate the phenotype rather than rescue it. Consequently, the hypothesis that absolute TAG quantity drives toxicity is contradicted by the authors' own data. It is more plausible that fat-6 RNAi rescues toxicity by preventing the formation of specific toxic lipid species (e.g., LCUFAs) rather than by addressing an energy deficit. To resolve this, the authors should perform lipidomic analysis on fat-6 and fat-7 RNAi worms in the αSyn background. Finally, the authors should verify whether fat-6 and fat-7 RNAi restores mitochondrial respiration and ATP levels to determine if the "energy rescue" mechanism holds true even when lipid stores are depleted.

2. The quantitative comparisons of Syn expression and aggregation in Figure 2 need to be more rigorous. In Figure 2a, the Western blot bands suggest unequal expression levels between WT, A53T, and A30P strains. Since the study relies on RNAi to control expression, comparable baseline expression is mandatory. The reduced signal in A53T might be due to insoluble aggregates failing to enter the SDS-PAGE gel; therefore, I suggest quantifying mRNA levels to confirm equal expression. Additionally, regarding Figure 2b, if the authors conclude that aggregation activity correlates with LD reduction, aggregation itself must be quantified rather than relying on qualitative images. Similarly, for the neuronal expression data in Figure 2d, quantitative data on expression and aggregation are needed.

3. Regarding the rescue by MCT feeding in Figure 6, there is a significant contradiction between the data and the proposed "energy deficit" model. The authors state in the Discussion that MCTs may "support mitochondrial ATP production." However, Figure 6f shows that MCT feeding reduces basal mitochondrial respiration in aSyn-YFP worms compared to untreated aSyn-YFP worms. If the primary driver of toxicity is an energy deficit, one would expect a rescue treatment to increase respiration and ATP turnover, not suppress it further. Notably, the respiratory profile presented in Figure 6f appears to show only FCCP and sodium azide injections, suggesting that oligomycin may not have been used. If oligomycin was indeed omitted, it is technically impossible to distinguish "ATP-linked respiration" from "proton leak." To support the conclusion that MCTs restore energy homeostasis despite lower respiration, the authors must measure total ATP levels. This is essential to determine whether MCT feeding maintains ATP levels via improved efficiency or if the rescue mechanism is entirely non-metabolic.

4. Throughout the manuscript (Figures 1, 2, and 4), Oil Red O (ORO) intensity is used to quantify fat content. However, the text notes that Syn expression and fat-6 RNAi significantly reduce worm size. Consequently, a reduction in total ORO intensity might simply reflect the smaller body volume rather than a metabolic defect. If such normalization has not been performed, the ORO intensity data should be normalized by body volume or size to ensure the phenotype represents a true reduction in lipid density.

5. In Figure 3c, the authors report Perilipin (PLIN-1) mislocalization. However, it is unclear from the current data whether PLIN-1 is dissociating from existing LDs, or if the LDs themselves are absent (a possibility supported by the author's note in the Discussion regarding "reduction in LD surface area"). To distinguish between a "reduction in LD number" and a "loss of PLIN-1 coating on intact LDs," the authors should perform co-staining with a neutral lipid dye alongside the PLIN-1 marker.

6. In Figure 5e, the authors show motility rescue in the membrane-binding deficient mutant (aSyn-delta) . It is essential to characterize the bioenergetic status of this mutant. Specifically, the authors should verify whether this mutant prevents mitochondrial fragmentation and, crucially, whether it restores ATP levels to baseline. Confirming that the loss of membrane binding prevents the energy deficit is necessary to substantiate the claim that membrane interaction is the upstream cause of the bioenergetic failure.

Minor Comments

7. In Figures S3b and S3c, there appears to be a discrepancy between the number of data points plotted on the graph and the 'n' number stated in the figure legend. Please ensure the data presentation and legends are accurate and consistent.

Manuscript ID: EMBOR-2025-63388V1

Title: Condensate-Driven Triglyceride Shift Links α-Synuclein to Mitochondrial Dysfunction

Dear Dr. Schnapp,

We sincerely thank you and the reviewers for the thoughtful and constructive comments on our manuscript entitled "Condensate-Driven Triglyceride Depletion Links α-Synuclein to Mitochondrial Dysfunction" (Manuscript ID: EMBOR-2025-63388V1). In response to these comments, we have carefully revised the manuscript and conducted additional experiments and analyses to strengthen the major conclusions of the study. We believe that these revisions have substantially improved the scientific rigor, clarity, and overall quality of the manuscript. Below, we provide a detailed point-by-point response to each comment, and all changes have been highlighted in the revised manuscript for ease of reference.

Referee #1:

This study shows that Parkinson's disease-linked α-synuclein (αSyn) disrupts lipid metabolism and cellular function in *C. elegans*. Lipidomic analyses reveal progressive triacylglycerol (TAG) depletion and accumulation of long-chain unsaturated fatty acids (LCUFAs) upon αSyn expression. Importantly, TAG loss depends on αyn aggregation: inclusion-forming variants (WT, A53T) induce marked TAG depletion, whereas the non-aggregating A30P variant does not. αSyn also disrupts lipid droplet architecture and mislocalizes PLIN-1, impairing lipid storage. These lipid defects are linked to mitochondrial fragmentation, reduced respiration, and motility decline. RNAi screening shows that suppressing LCUFA synthesis (*fat-6*/*fat-7* knockdown) rescues motility, whereas restoring total TAG levels alone does not, indicating that fatty-acid composition-not TAG abundance-drives toxicity. αSyn accumulates near mitochondria, suggesting that altered TAG availability and lipid composition underlie mitochondrial dysfunction and neurotoxicity. This manuscript is well written, and can reach the level of publication after revision.

Major comment 1:

There is a mechanistic gap between condensates and TAG depletion. While the paper demonstrates that TAG depletion depends on αSyn's ability to form condensates/inclusions, the actual mechanism remains unclear. The authors note minimal colocalization between αSyn inclusions and lipid droplets at later stages (Fig. 3c, S3a, S3d), suggesting indirect effects, but they don't adequately explain how condensates cause TAG depletion. Is it through sequestration of lipid metabolic enzymes? Disruption of ER-LD contacts? Altered gene expression? The paper needs experiments to directly test these possibilities rather than relying primarily on correlative observations.

We thank the reviewer for this comment. We agree that understanding how αSyn condensate formation is mechanistically translated into TAG depletion is an important question.

First, our data do not fully support a single model in which αSyn inclusions directly sequester LDs. We observed colocalization between aSyn-YFP inclusions and the LD marker PLIN-1::mCherry (Fig. S3D). We cannot exclude that monomeric or other soluble forms of lipid-binding competent alpha-synuclein, not yet associated with inclusion, do disturb LD function.

Second, regarding the possibility of altered transcription of lipid metabolic enzymes, we performed additional qRT-PCR analyses of several major regulators of fatty acid metabolism, including *elo-2*, *fat-5*, *fat-6*, and *fat-7*. As shown in the revised new Fig. S4H, SNCA RNAi did not induce a broad transcriptional alteration of these lipid metabolic genes. The expression levels of *elo-2*, *fat-6*, and *fat-7* were not significantly altered, whereas *fat-5* expression was selectively increased. These findings argue against a global transcriptional reprogramming of TAG metabolism as the primary explanation for the TAG phenotype. Rather, they suggest that selective regulation of lipid desaturation pathways or selective depletion of certain lipid species may contribute to αSyn-associated lipid dysregulation. (Revised in line 22-27, page 8).

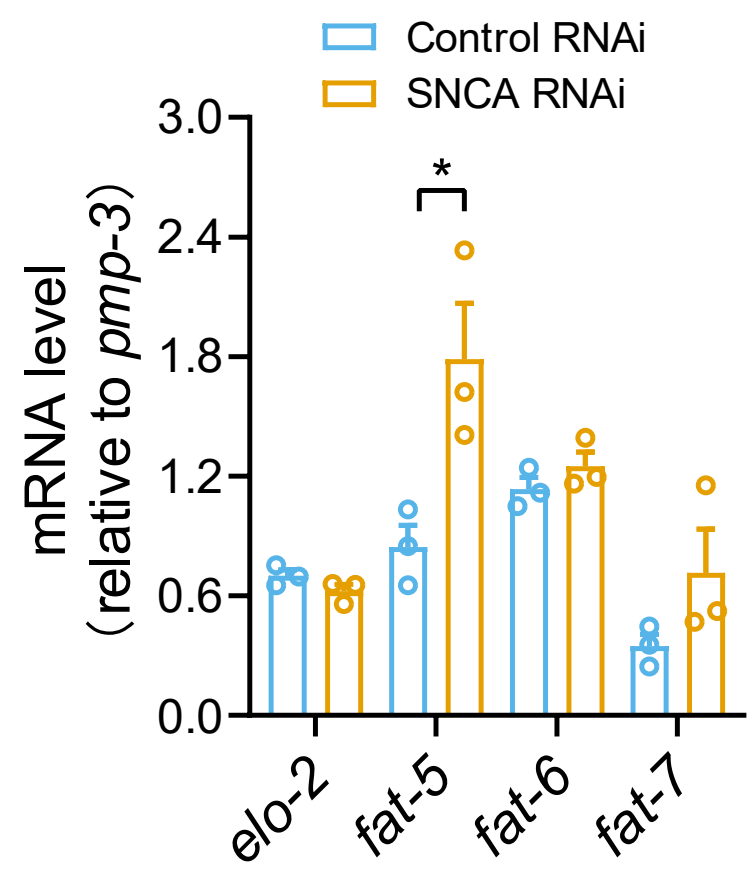

Fig. S4H. Expression levels of *elo-2*, *fat-6*, and *fat-7* show no significant changes compared with control RNAi-treated worms, whereas *fat-5* expression is selectively increased following SNCA RNAi.

Third, we agree that disruption of ER-LD contacts and sequestration of lipid metabolic enzymes cannot be excluded by our current data. Importantly, PLIN-1 is a structural LD-associated protein involved in LD stability and TAG mobilization, but it is not itself a canonical ER-LD tether. We have therefore revised the manuscript to avoid interpreting the altered PLIN-1 distribution as direct evidence of ER–LD contact disruption. Likewise, we currently do not have direct evidence demonstrating that αSyn inclusions sequester specific TAG metabolic enzymes. We now explicitly acknowledge these possibilities as remaining mechanistic questions rather than presenting them as established mechanisms.

To further investigate whether αSyn condensation is associated with cellular organelle dysfunction beyond LDs, we performed additional mitochondrial analyses. Using TOMM-20::mRFP, we observed pronounced mitochondrial fragmentation in αSyn-YFP worms at day 5 of adulthood, reflected by reduced mitochondrial aspect ratio and mitochondrial area (new Fig. 6D,E). In contrast, aSyn(Δ)-YFP worms did not exhibit apparent mitochondrial fragmentation under the same conditions. Importantly, we further observed clear colocalization between WT αSyn(WT) inclusions and TOMM-20-labeled mitochondria (new Fig. 6F). Thus, αSyn(WT), but not the lipid-binding deletion construct, is associated with mitochondria and mitochondrial morphological abnormalities. (Revised in line 29-32, page 9).

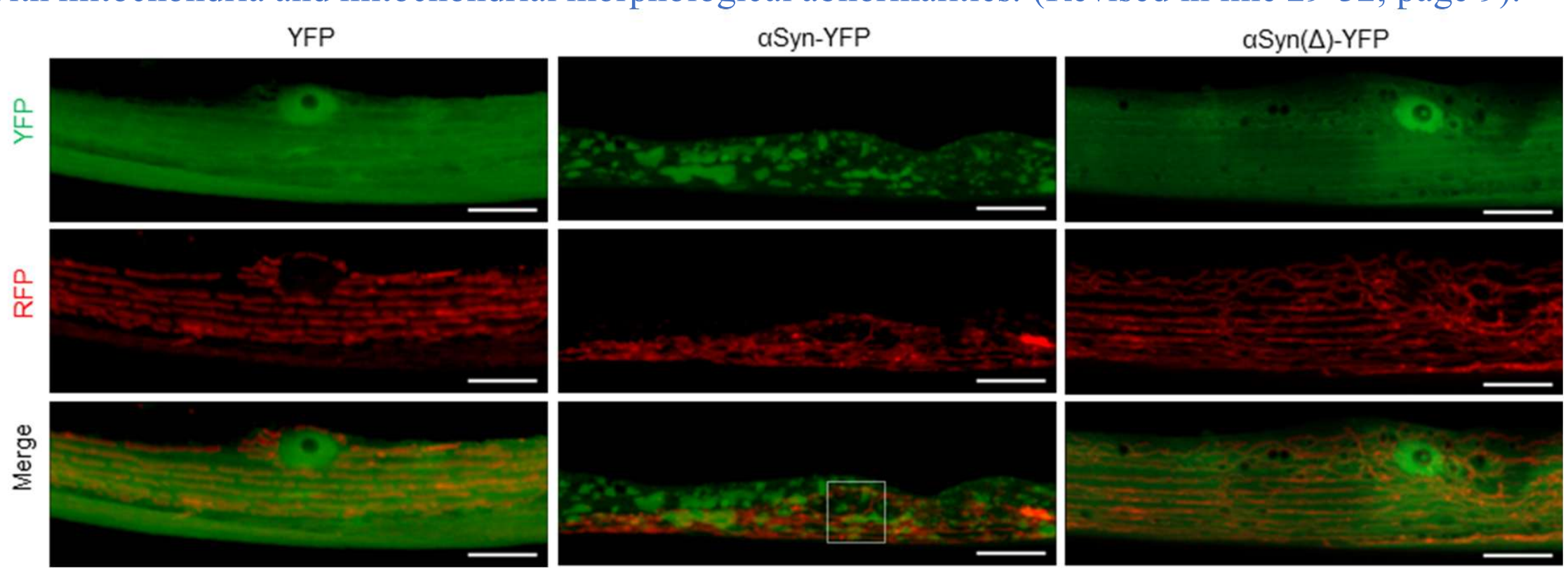

Fig. 6D. Representative TOMM-20::mRFP images reveal increased mitochondrial fragmentation in αSyn(WT)-YFP worms, whereas this phenotype is not apparent in αSyn(Δ)-YFP worms expressing a lipid-binding-deficient αSyn variant. Scale bar, 10 μm.

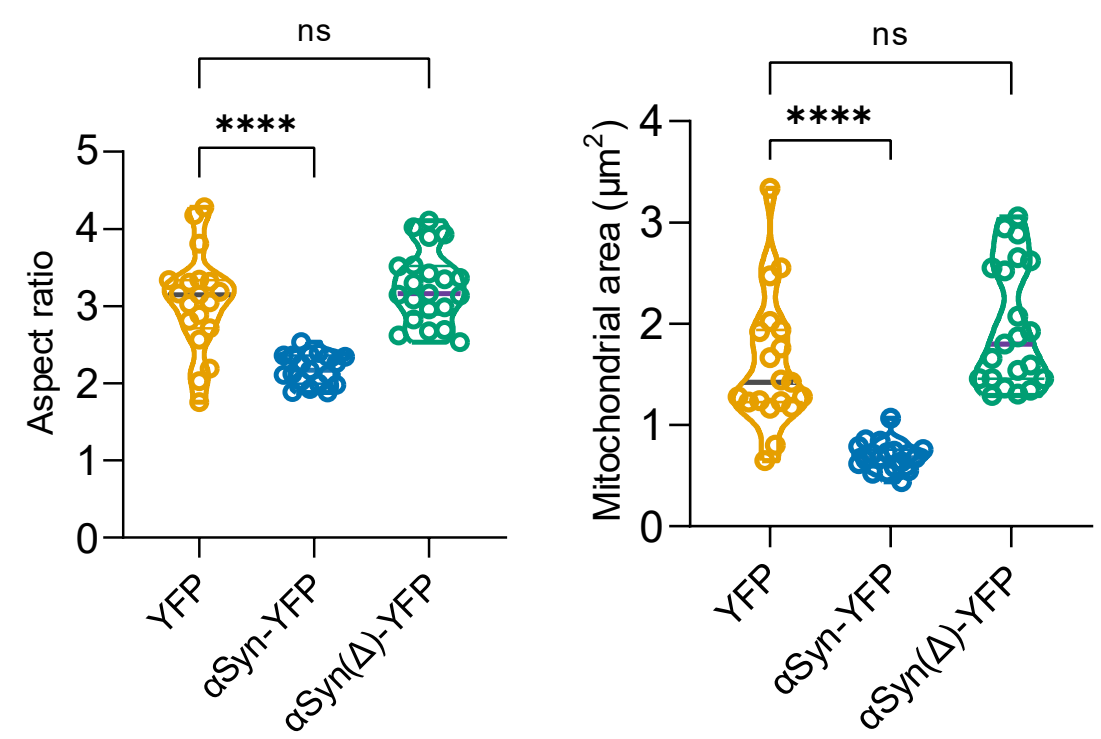

Fig. 6E. Quantification of mitochondrial morphology shows reduced mitochondrial aspect ratio and area in αSyn(WT)-YFP worms compared with YFP and αSyn(Δ)-YFP worms at day 5 of adulthood (n ≥ 19 worms per condition).

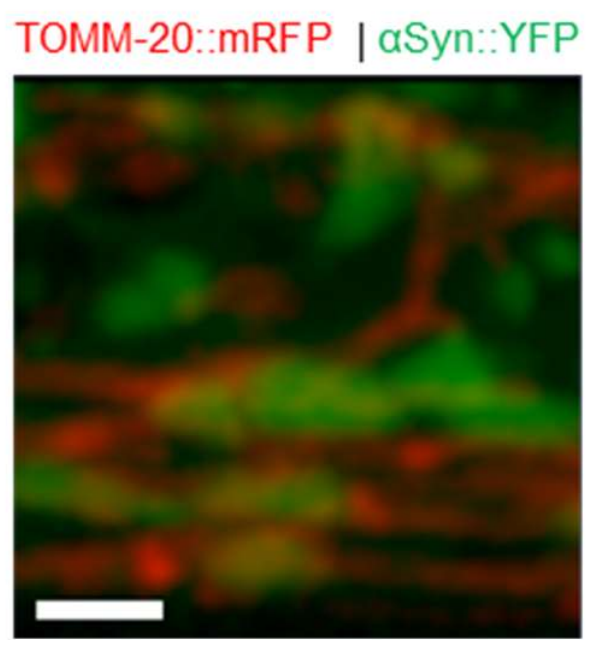

Fig. 6F. Representative image showing spatial colocalization of αSyn inclusions with mitochondria in αSyn-YFP worms. Scale bar, 2 μm.

Together with our previous observation that both lipid binding and condensation/inclusion formation are required for the TAG phenotype, these findings support a model (new Fig. 6A ; Fig. 7D, upper panel) in which αSyn condensation is associated with broader perturbation of intracellular lipid and organelle homeostasis. In particular, the mitochondrial phenotype provides evidence that αSyn toxicity is not restricted to LDs and suggests that mitochondrial dysfunction may represent an additional cellular consequence of αSyn toxicity. (Revised in line 12-18, page 11).

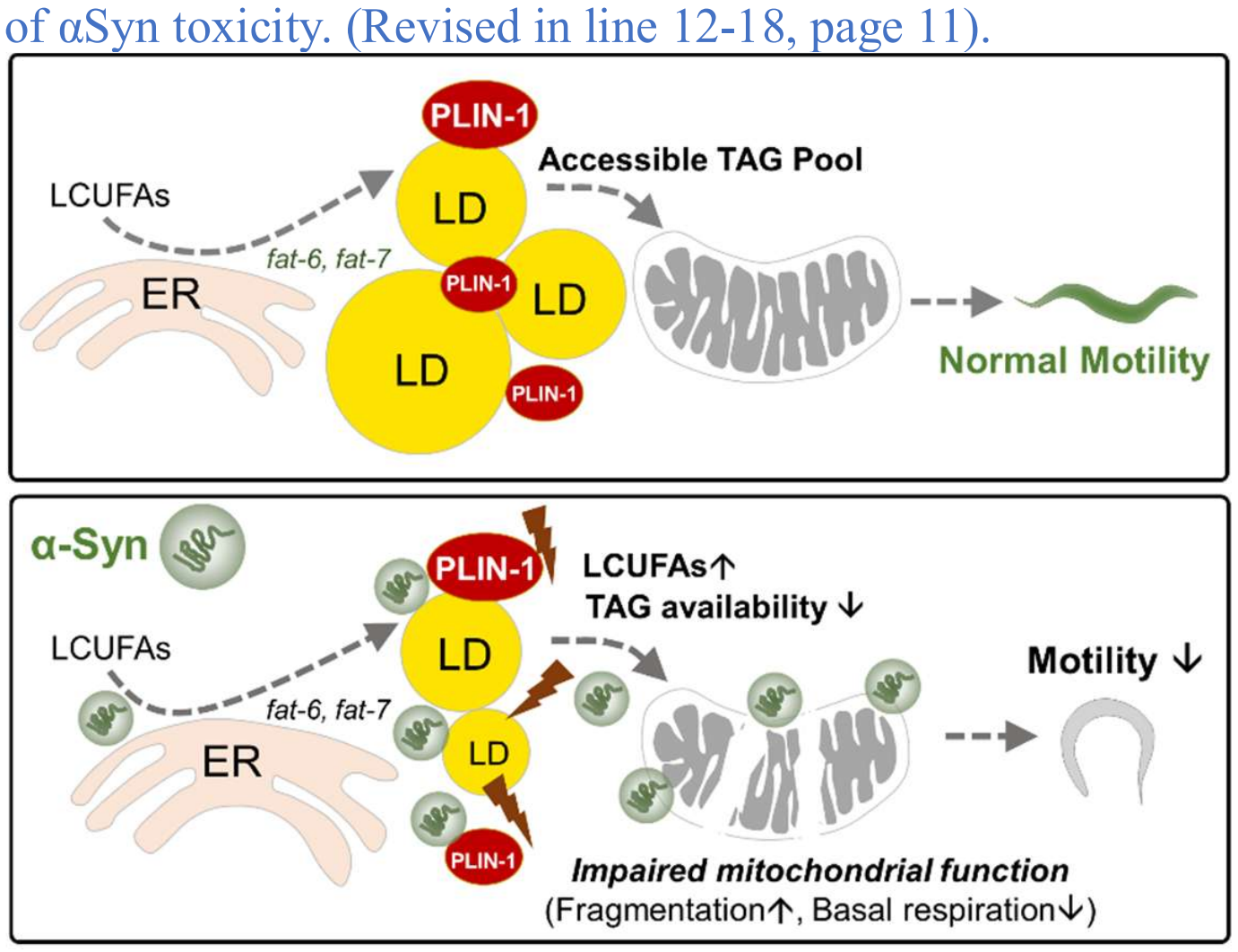

Fig. 6A and Fig. 7D. αSyn inclusion formation is associated with impaired LD biogenesis and PLIN-1 mislocalization, potentially disrupting TAG availability and mitochondrial function (upper panel).

We have revised the manuscript accordingly and emphasize that αSyn condensation is associated with coordinated defects in LD organization, TAG homeostasis, and mitochondrial function, while the precise molecular link between αSyn condensates and TAG depletion remains to be determined.

Major comment 2:

There is an inconsistency for fat-6 and fat-7 results. The differential effects of fat-6 versus fat-7 manipulation are confusing and inadequately addressed. Fat-6 RNAi reduces both TAG levels and αSyn expression while also decreasing worm size, which the authors attribute to "systemic effects." However, fat-7 RNAi restores TAG levels and rescues motility without affecting size. Yet fat-7 overexpression increases αSyn levels and impairs motility. These seemingly contradictory results suggest complex, potentially indirect mechanisms that deserve dedicated experiments and discussion rather than the brief speculation provided.

We thank the reviewer for highlighting this point. We agree that the differential effects of *fat-6* and *fat-7* manipulation represent a complex aspect of our study. Our results indicate that although both genes encode stearoyl-CoA desaturases involved in unsaturated fatty acid synthesis, their contributions to αSyn toxicity are not equivalent. We have expanded our analyses and revised the discussion to better address this distinction.

First, to further explore potential reasons underlying the different responses to *fat-6* and *fat-7* manipulation, we analyzed their basal expression patterns using the single-cell RNA-seq atlas from Roux et al. (2023). As shown in new Fig. S4E, *fat-6* exhibits broad expression across multiple tissues, whereas *fat-7* displays much lower basal expression in the tissues profiled, including body-wall muscle. These expression differences may contribute to the distinct consequences of systemic RNAi. Widespread inhibition of *fat-6* is expected to affect multiple physiological processes, consistent with the observed reduction in body size, whereas *fat-7* perturbation may produce a more selective effect on lipid metabolism and αSyn toxicity. (Revised in line 12-19, page 8).

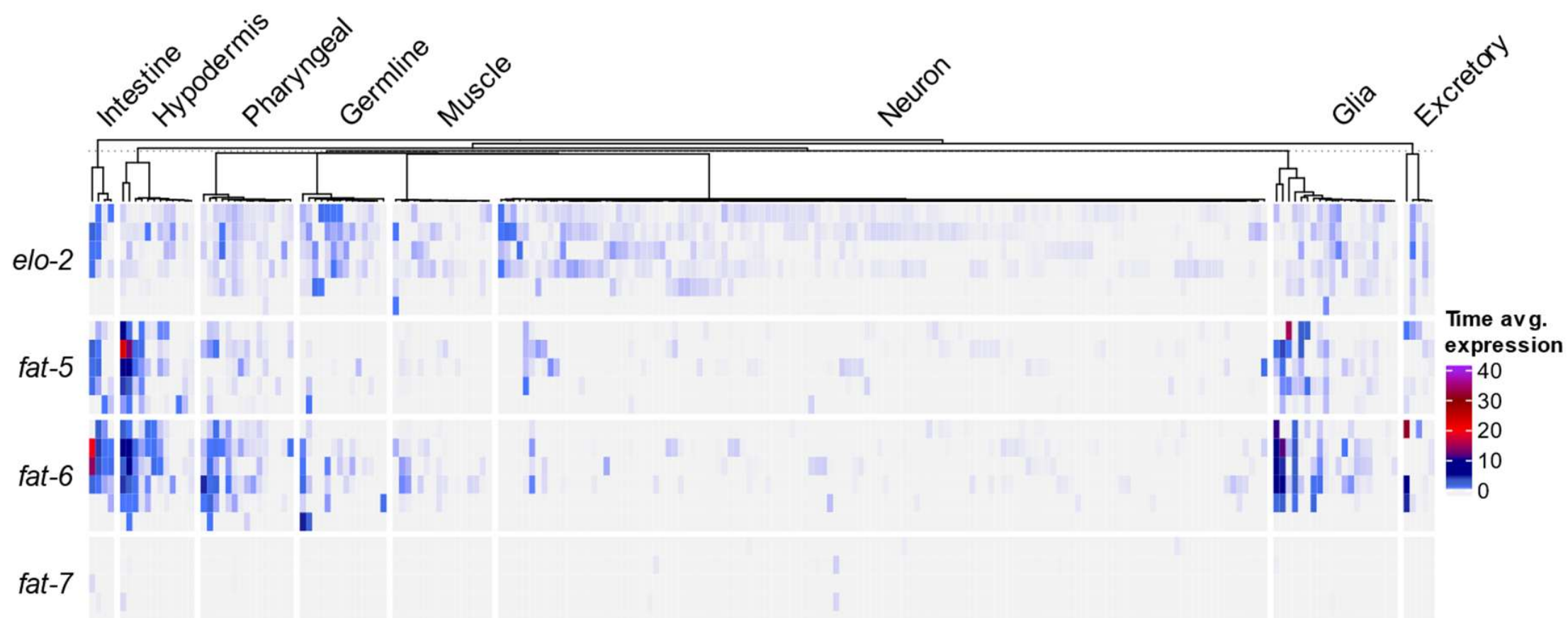

Fig. S4E. Time-averaged expression in wild-type worms reveals *fat-6* shows broad and high-level expression across most tissues, while *fat-7* is nearly undetectable in all tissues examined including body-wall muscle.

Second, to further distinguish the potential differences between *fat-6* and *fat-7* RNAi, we performed additional experiments examining multiple αSyn-associated phenotypes. We observed no significant differences in αSyn transcript levels or αSyn inclusion formation between *fat-6* and *fat-7* RNAi conditions (new Fig. S4B and Fig. S4C). These data indicate that the observed effects are unlikely to be attributable to differential regulation of αSyn aggregation between *fat-6* and *fat-7* RNAi treatments. (Revised in line 8-12, page 8).

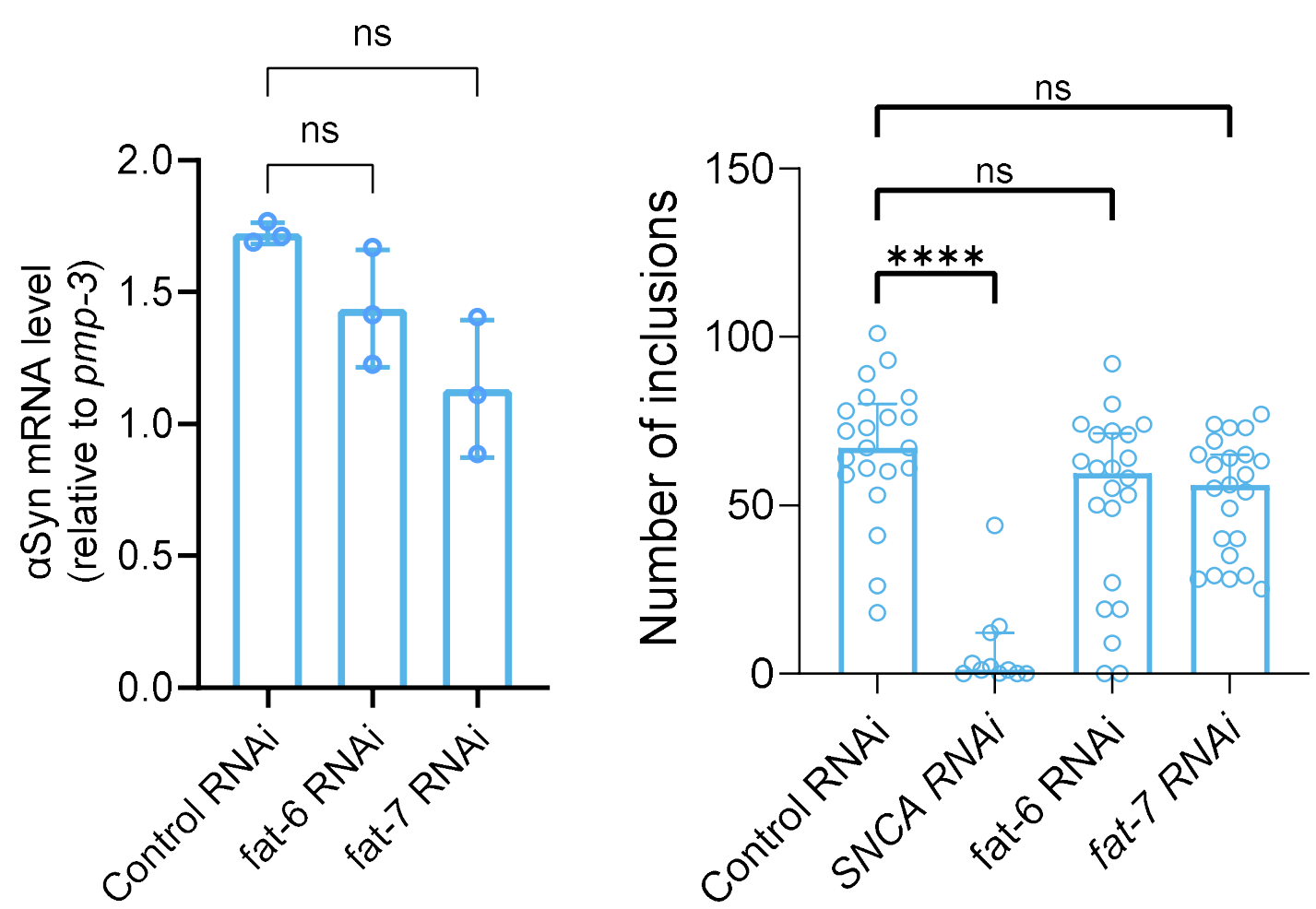

Fig. S4(B) and Fig. S4(C). qPCR analysis showing that *fat-6* or *fat-7* RNAi does not significantly altered SNCA mRNA levels in day 5 adult αSyn-YFP worms (one-way ANOVA). Quantification of αSyn inclusion numbers showing no significant effect of *fat-6* or *fat-7* RNAi in day 5 adult αSyn-YFP worms (one-way ANOVA).

Together, this result indicates that *fat-6* and *fat-7*, despite their shared biochemical function in fatty acid desaturation, have distinct physiological roles. We have revised the discussion to clarify that the *fat-6* and *fat-7* divergence likely reflects differences in tissue expression and systemic metabolic effects rather than simply differences in enzymatic function.

Referee #2:

This study employed a nematode aging model combined with untargeted lipidomics, live imaging, RNAi functional screening, and clinical big-data meta-analysis to systematically elucidate that α-synuclein aggregates drive motor deficits through a coherent axis of "decreased total TAG levels-elevated proportion of long-chain unsaturated fatty acids-mislocalization of lipid droplet structural protein PLIN-1-impaired mitochondrial respiration." The authors demonstrated the conservation of reduced serum TAG levels in Parkinson's patients across species. They proposed an intervention strategy involving dietary medium-chain fatty acids to bypass the LCUFA synthesis blockade and directly restore mitochondrial energy metabolism. This study provides a mechanistically clear, operationally simple, and highly translatable approach for the metabolic regulation therapy of synucleinopathies. Certain concerns need to be addressed before it is considered for publication.

Comment 1:

The authors only tested 0.5% and 1.0% MCT, lacking a dose-response curve. Moreover, does MCT affect αSyn aggregation per se? Similarly, while fat-6 and fat-7 influence the expression level of αSyn, what would be their effects on αSyn aggregation?

We thank Referee #2 for these complementary points. To determine whether MCT directly influences αSyn expression or aggregation, we performed additional experiments examining αSyn protein abundance, inclusion formation and motility following supplementation with a broad range of MCT concentrations (0.05%, 0.10%, 0.25%, 0.50%, 1.00%, and 2.00%), with matched LCT controls.

As shown in the revised new Fig. 7A, MCT supplementation improved the motility defect of αSyn-YFP animals in a dose-responsive manner, with maximal rescue observed at intermediate-to-high concentrations (0.25%~1.00%). However, MCT supplementation did not significantly alter αSyn-YFP protein levels across the tested concentration range (new Fig. S8B). Notably, quantitative analysis of αSyn-YFP inclusions revealed no significant differences in inclusion number following either MCT or LCT supplementation (new Fig. 7C). These results indicate that the beneficial effects of MCT are unlikely to be mediated by reducing αSyn expression or suppressing αSyn aggregation. Instead, MCT

appears to act downstream of αSyn condensation. The new data is consistent with our observation that MCT, but not LCT, improves αSyn-associated motility defects. (Revised in line 9-19, 30-32, page 10).

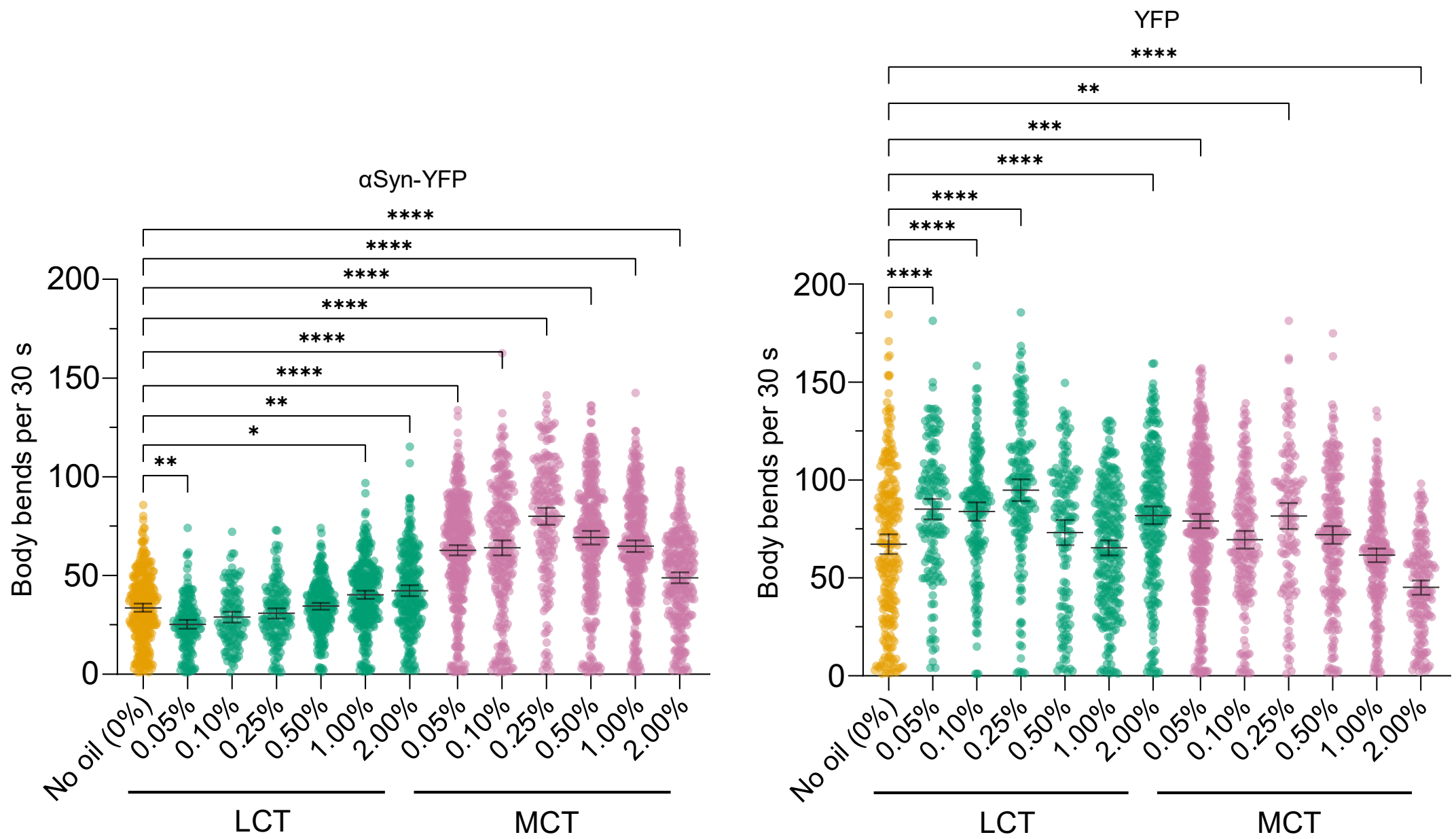

Fig. 7A. MCT supplementation dose-dependently rescues motility defects in αSyn-YFP worms but impairs motility in YFP controls at high doses.

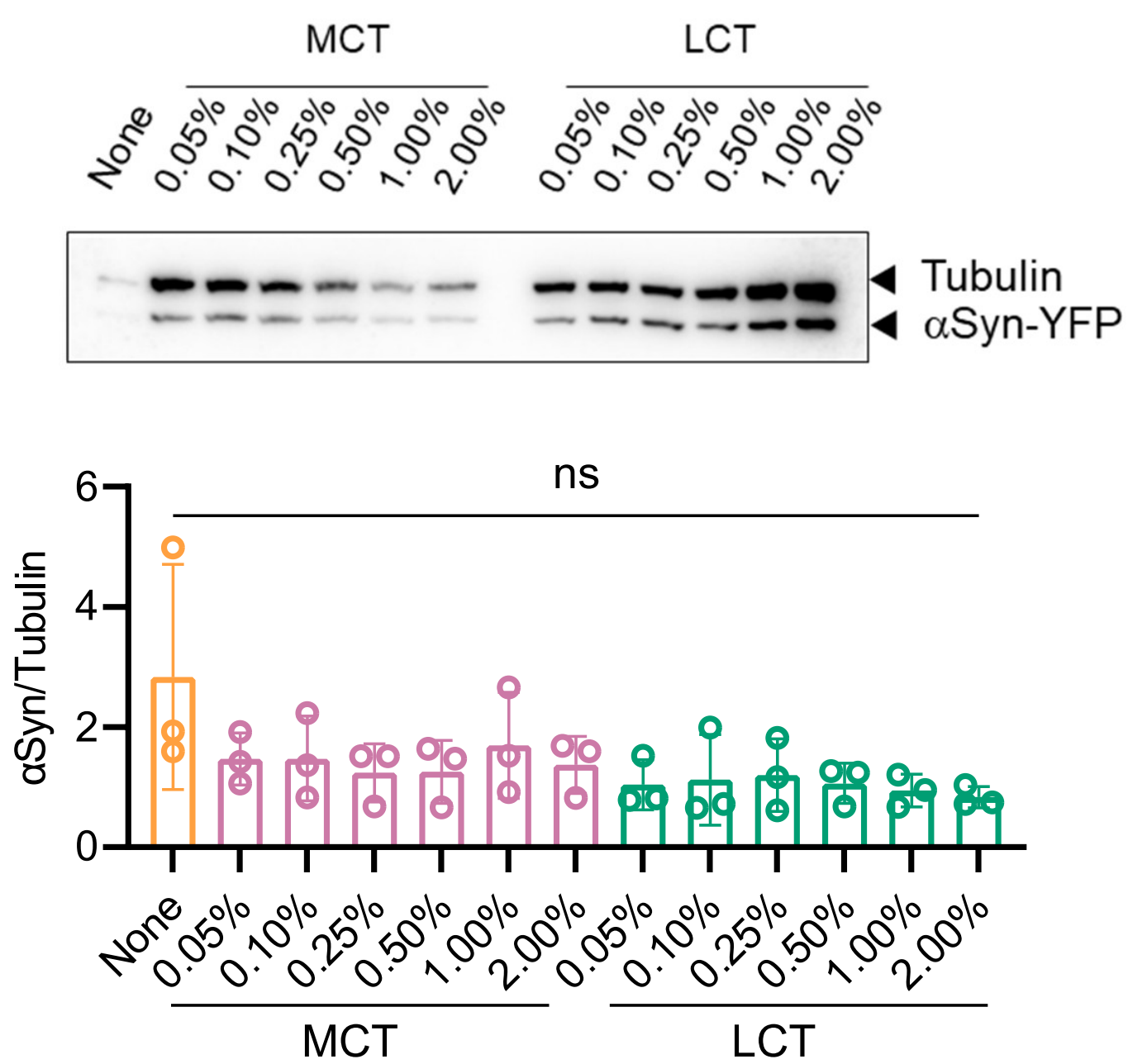

Fig. S8B. MCT and LCT supplementation do not significantly alter αSyn protein abundance in αSyn-YFP worms.

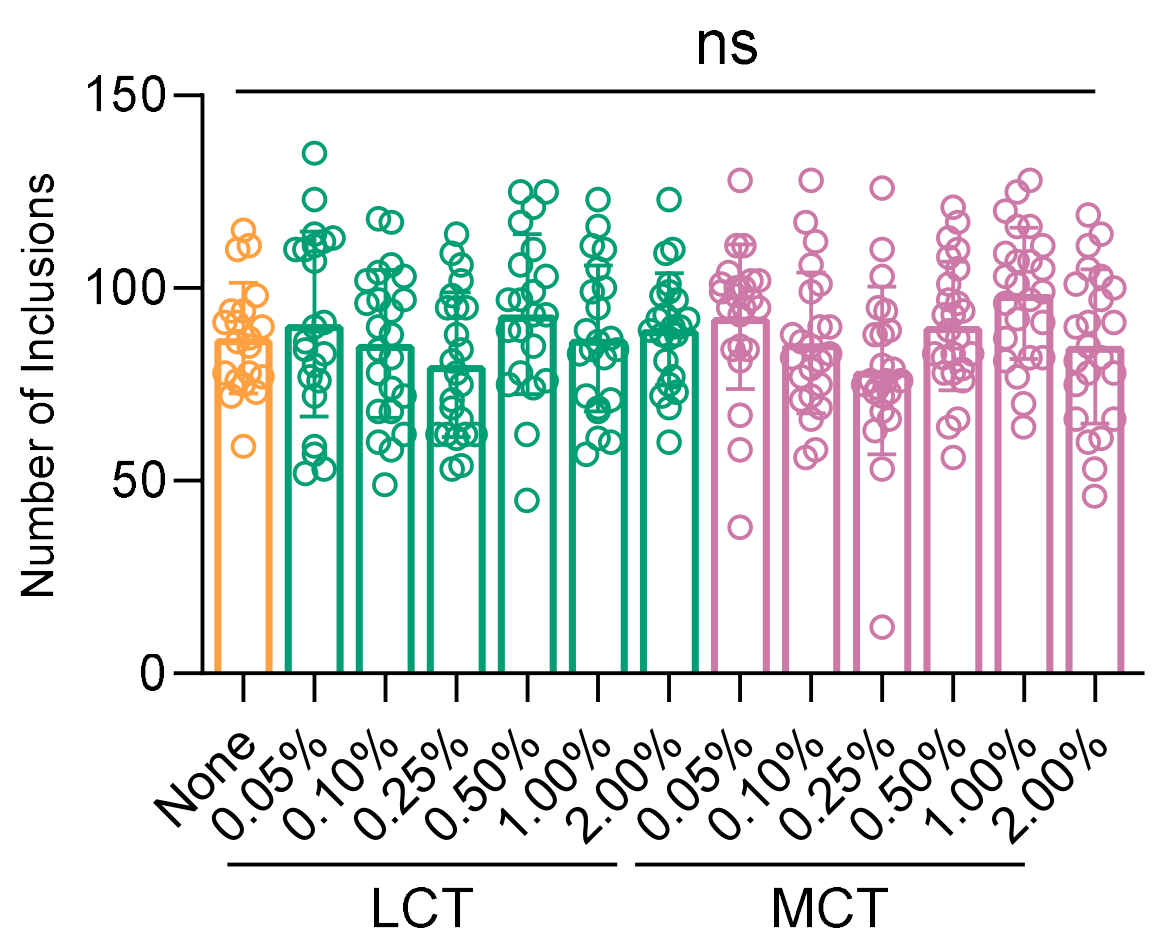

Fig. 7C. MCT and LCT supplementation did not significantly alter the number of αSyn inclusions in day-5 adult αSyn-YFP worms (one-way ANOVA).

According to comments, we also performed additional experiments to investigate whether *fat-6* and *fat-7* alter αSyn aggregation. We found that RNAi-mediated depletion of *fat-6* and *fat-7* did not significantly change the number of αSyn inclusions or mRNA level compared with the control RNAi (new Fig. S4B; Fig. S4C). Thess results demonstrate that although *fat-6* and *fat-7* regulate αSyn-associated toxicity and lipid metabolic phenotypes, their effects are unlikely to be mediated through altering αSyn mRNA level or αSyn aggregation. Instead, the new data supports a model in which lipid desaturation pathways modulate the cellular consequences of αSyn pathology. (Revised in line 8-12, page 8).

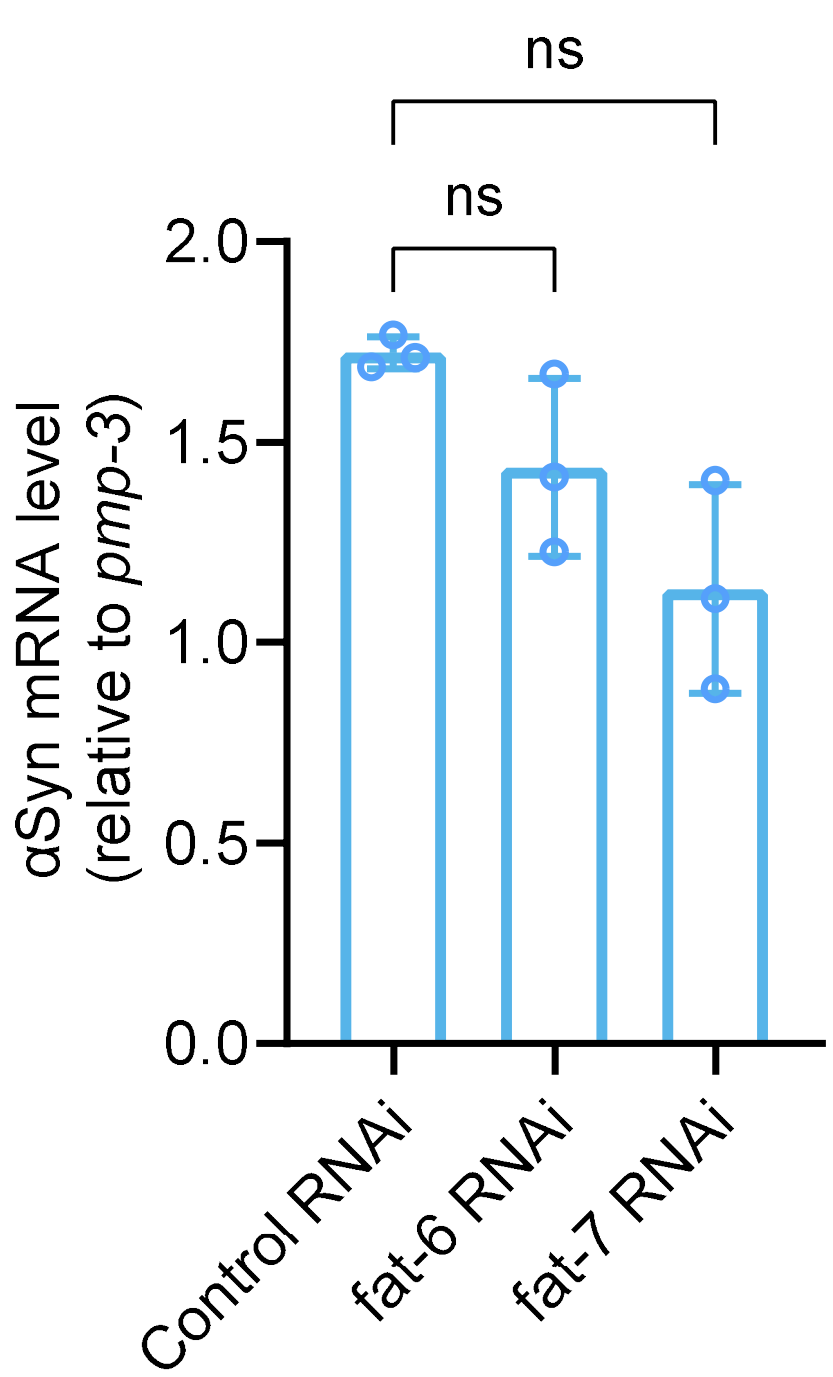

Fig. S4B qPCR analysis showing that *fat-6* or *fat-7* RNAi does not significantly alter SNCA mRNA levels in day 5 adult αSyn-YFP worms (one-way ANOVA).

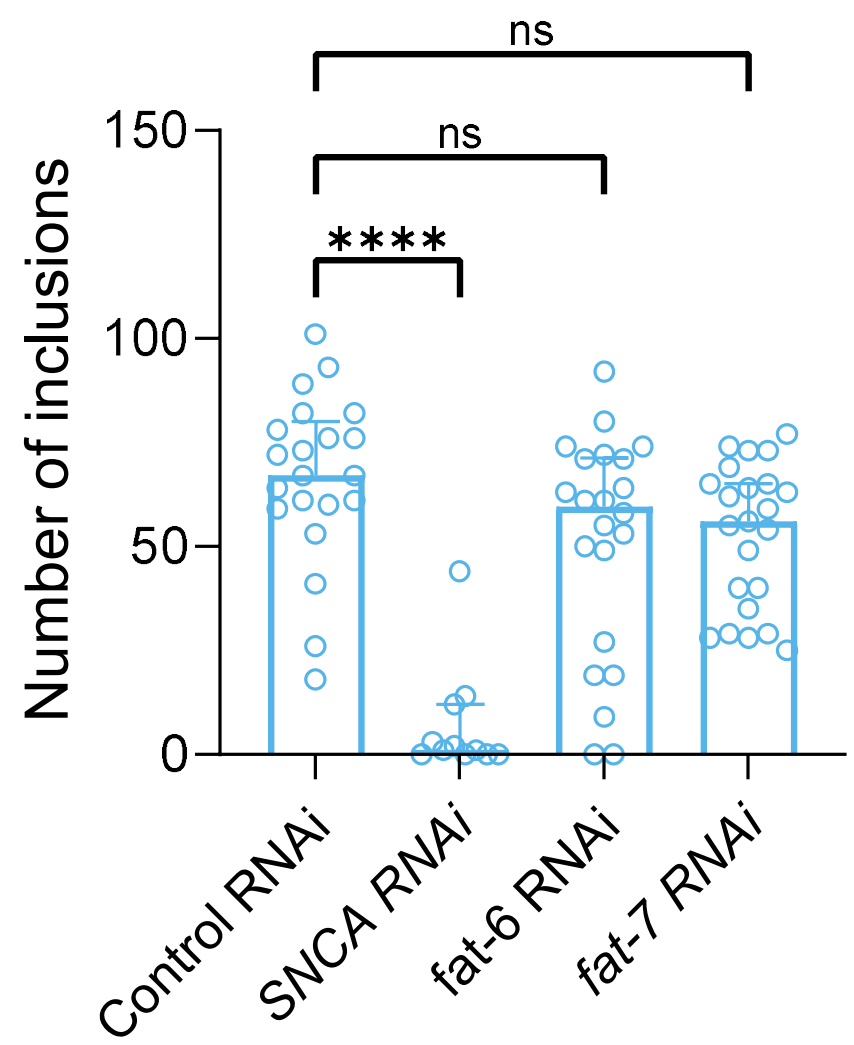

Fig. S4C. Quantification of αSyn inclusion numbers showing no significant effect of *fat-6* or *fat-7* RNAi in day 5 adult αSyn-YFP worms (one-way ANOVA).

Comments 2 and 3:

2. On page 8, line 24, "dirsupt" is a spelling error, which should be corrected to "disrupt". On page 12, line 12, "subcellullar" is a spelling error, which should be corrected to "subcellular". On page 15, line 4, "quanlity" is a spelling error, which should be corrected to "quality". On page 16, line 8, "fluoresence" is a spelling error, which should be corrected to "fluorescence". On page 17, line 9, "meaurement" is a spelling error, which should be corrected to "measurement". The author is advised to review and revise the entire text accordingly.

We thank the reviewer for identifying these typographical errors. We have corrected those errors, respectively. We have also carefully proofread the entire manuscript and corrected additional typographical and grammatical errors where necessary.

3. The manuscript contains some incorrect figure references, for example： "Indeed, motility in αSyn-YFP worms was significantly improved when fed 0.5% or 1.0% MCT compared to YFP controls, whereas feeding LCT did not improve motility (Fig 6c)." This should be changed to "fig 6e". "In addition, mitochondrial respiration measurements revealed that αSyn-YFP worms respond to MCT feeding but not LCT, while mitochondria of YFP worms respond to both MCT and LCT feeding (Fig. 6e). " This should be changed to "fig 6f".

We thank the reviewer for the careful reading. All typographical errors have been corrected, and the incorrect figure references have also been fixed.

Minor comments:

In Figures S3b and S3c, there appears to be a discrepancy between the number of data points plotted on the graph and the 'n' number stated in the figure legend. Please ensure the data presentation and legends are accurate and consistent.

We thank the reviewer for this careful check of the figure legends. The discrepancy in Figures S3b and S3c has been corrected, and all data points now accurately reflect the stated *n* numbers. The source data file has been updated accordingly.

Referee #3:

Summary: This manuscript investigates the link between aSyn toxicity and lipid metabolism in C. elegans. The authors report that aSyn reduces TAG levels and disrupts LDs, proposing that these changes lead to mitochondrial dysfunction and impaired motility. They further suggest that fat6/7 RNAi or supplementing with medium-chain triglycerides (MCT) mitigates toxicity. While the connection between lipid metabolism and aSyn toxicity is of high interest, and the clinical correlation with PD patient serum is intriguing, the manuscript contains several logical inconsistencies and lacks critical quantitative validations. Specifically, the relationship between TAG levels, LCUFA composition, and mitochondrial bioenergetics remains contradictory. Major revisions are required to substantiate the proposed mechanism.

Major comment 1:

The title, "Condensate-Driven Triglyceride Depletion Links...", explicitly posits that the reduction in TAG levels is the causal mechanism driving mitochondrial dysfunction. However, the data demonstrates that fat-6 RNAi rescues motility despite causing a further reduction in lipid stores (as indicated by Oil Red O staining) (Fig. 4b, c). This result creates a logical paradox: if "triglyceride depletion" were the direct driver of toxicity, fat-6 RNAi should exacerbate the phenotype rather than rescue it. Consequently, the hypothesis that absolute TAG quantity drives toxicity is contradicted by the authors' own data. It is more plausible that fat-6 RNAi rescues toxicity by preventing the formation of specific toxic lipid species (e.g., LCUFAs) rather than by addressing an energy deficit. To resolve this, the authors should perform lipidomic analysis on fat-6 and fat-7 RNAi worms in the αSyn background. Finally, the authors should verify whether fat-6 and fat-7 RNAi restores mitochondrial respiration and ATP levels to determine if the "energy rescue" mechanism holds true even when lipid stores are depleted.

We thank the reviewer for raising this important conceptual point. We agree that our original title may suggest that a reduction in the total TAG levels due to alpha-synuclein expression causes mitochondrial dysfunction. While we do find that depletion of total TAG levels is one of the major lipidome changes induced by αSyn, our work more specifically suggests that the accompanied change in in composition of the remaining TAGs contributes to toxicity, such as an increase in LCUFA-containing TAGs and a decrease in shorter chain saturated fatty acids (SCFA)-containing TAGs. Accordingly, the title has been revised to "Condensate-Driven Triglyceride Shift Links α-Synuclein to Mitochondrial Dysfunction". Our MCT-feeding data suggest that next to an LCUFA-induced toxicity, a reduction in MCFA's also contributes to alpha-synuclein induced toxicity. These two lipidome-change related mechanisms can co-exist and do not exclude one another. (Revised in line 1, page 1).

To understand the seemingly contradicting results of *fat-6* and fat 7 RNAi, we performed additional targeted lipid analyses in αSyn-YFP. These results revealed that *fat-6* and *fat-7* RNAi significantly reduced the levels of LCUFAs, particularly C18:1 (n-9), in αSyn-YFP worms (in new Fig. 5E), which indicates that the protective effects of *fat-6*/*fat-7* RNAi arise at least in part from remodeling of fatty acid composition and by reducing potentially detrimental lipid species. (Revised in line 6-11, page 9).

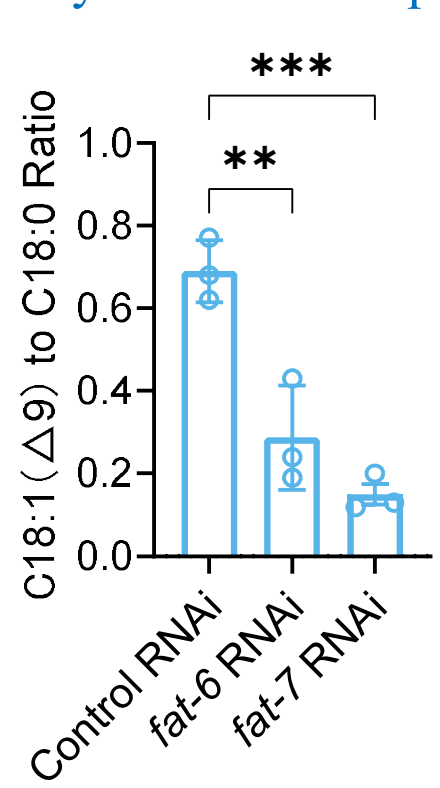

Fig. 5E. Quantification of C18:1 fatty acid abundance showing that *fat-6* and *fat-7* RNAi significantly reduces C18:1 levels in αSyn-YFP worms.

In addition, to investigate whether the protective effects of *fat-6* and *fat-7* RNAi involve restoration of cellular energy status, we measured ATP levels in αSyn-YFP worms following these interventions. Interestingly, both *fat-6* and *fat-7* RNAi resulted in reduced ATP levels in αSyn-YFP animals (Fig. R1). However, total ATP levels reflect the balance between ATP production and consumption and may thus not be representative of mitochondrial function alone. Therefore, these results do not allow us to determine whether energy status contributes to lipid-associated mechanisms of alpha-synuclein toxicity. We have revised the manuscript accordingly.

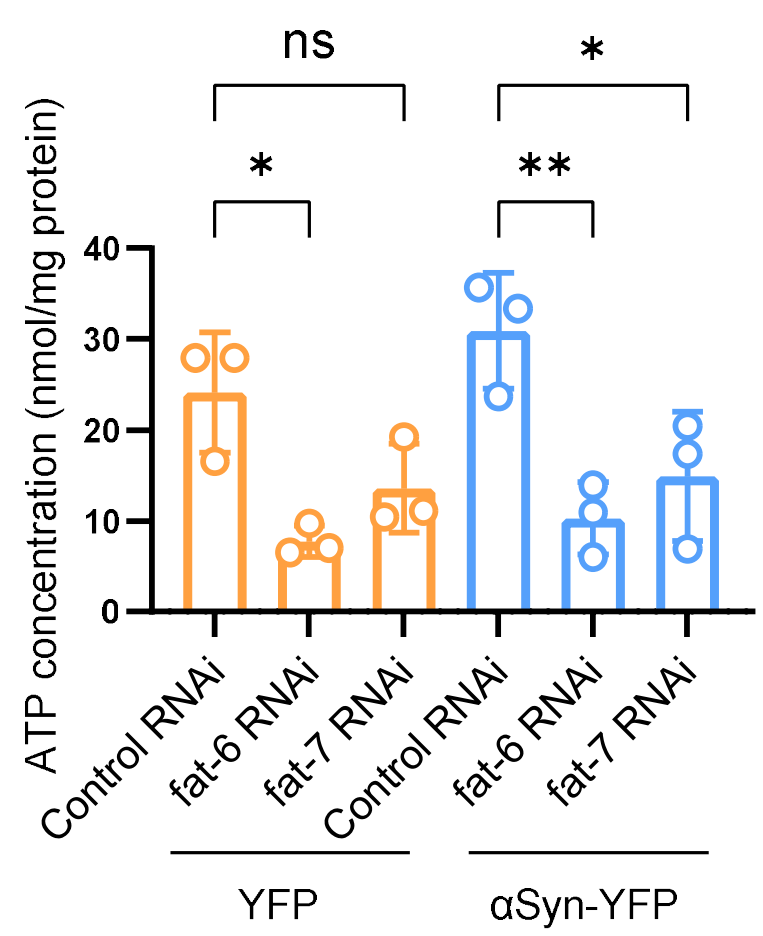

Fig. R1. *Fat-6* and *fat-7* RNAi reduce ATP abundance in αSyn-YFP worms, whereas only fat-6 RNAi significantly reduces ATP abundance in YFP control worms.

<u>Major comment 2:</u>

The quantitative comparisons of Syn expression and aggregation in Figure 2 need to be more rigorous. In Figure 2a, the Western blot bands suggest unequal expression levels between WT, A53T, and A30P strains. Since the study relies on RNAi to control expression, comparable baseline expression is mandatory. The reduced signal in A53T might be due to insoluble aggregates failing to enter the SDS-PAGE gel; therefore, I suggest quantifying mRNA levels to confirm equal expression. Additionally, regarding Figure 2b, if the authors conclude that aggregation activity correlates with LD reduction, aggregation itself must be quantified rather than relying on qualitative images. Similarly, for the neuronal expression data in Figure 2d, quantitative data on expression and aggregation are needed.

We thank the reviewer for this important comment. To address the potential differences in αSyn expression among the muscle-expressing αSyn strains, we performed additional quantitative analyses of both αSyn protein and transcript levels.

First, as shown in the new Fig. 2A in the revised manuscript, αSyn protein abundance normalized to tubulin was not significantly different among αSyn(WT)-YFP, αSyn(A30P)-YFP, and αSyn(A53T)-YFP worms. Although the raw Western blot images showed variation in band intensity, quantitative densitometric analysis demonstrated comparable αSyn protein expression across these strains. Furthermore, qRT-PCR analysis showed that αSyn-YFP mRNA and YFP levels were not significantly different among the three αSyn-expressing strains (new Fig. S1C and S1D). These results indicate that the differences in lipid phenotypes among the αSyn variants are unlikely to be attributed to differences in αSyn expression levels. (Revised in line 30-32, page 5).

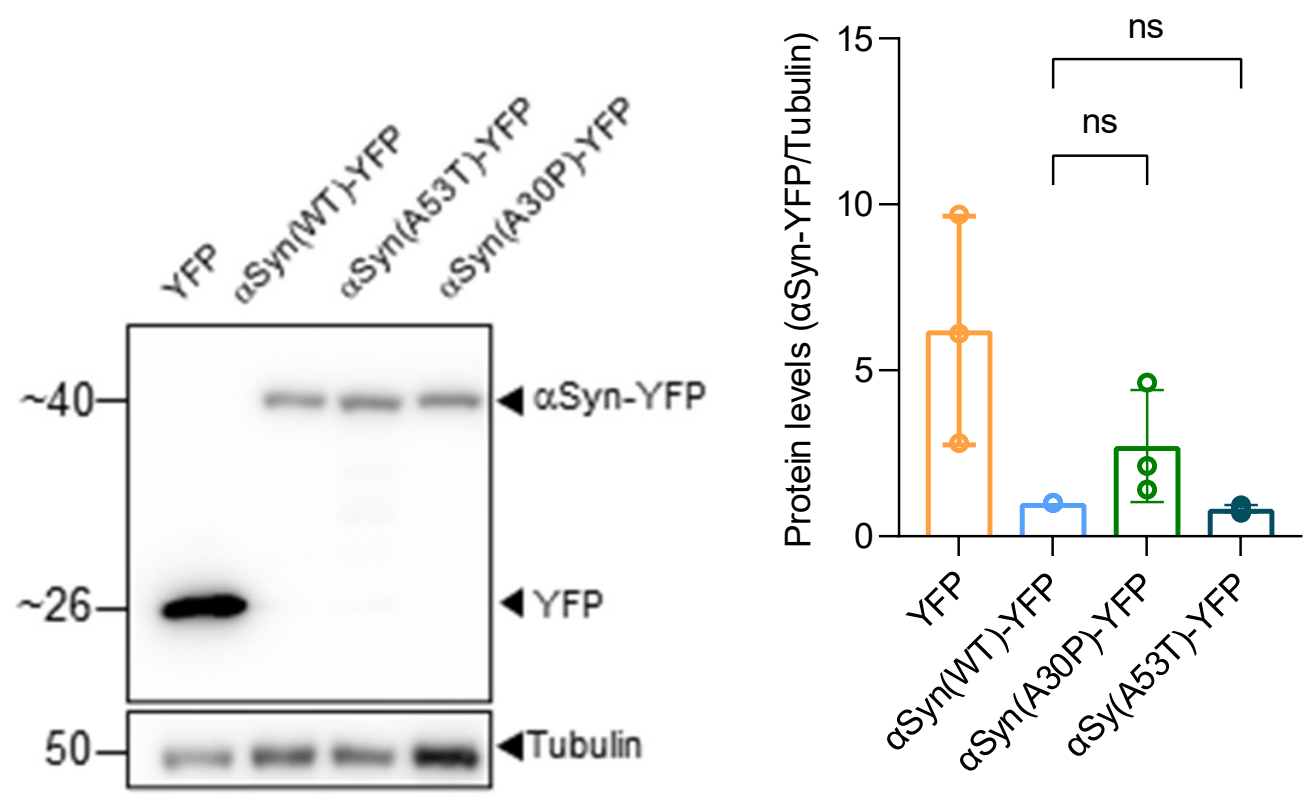

Fig. 2A. Western blot analysis shows comparable αSyn expression levels in αSyn(WT)-YFP, αSyn(A30P)-YFP, and αSyn(A53T)-YFP worms using an anti-GFP antibody. Tubulin was used as control (n=3).

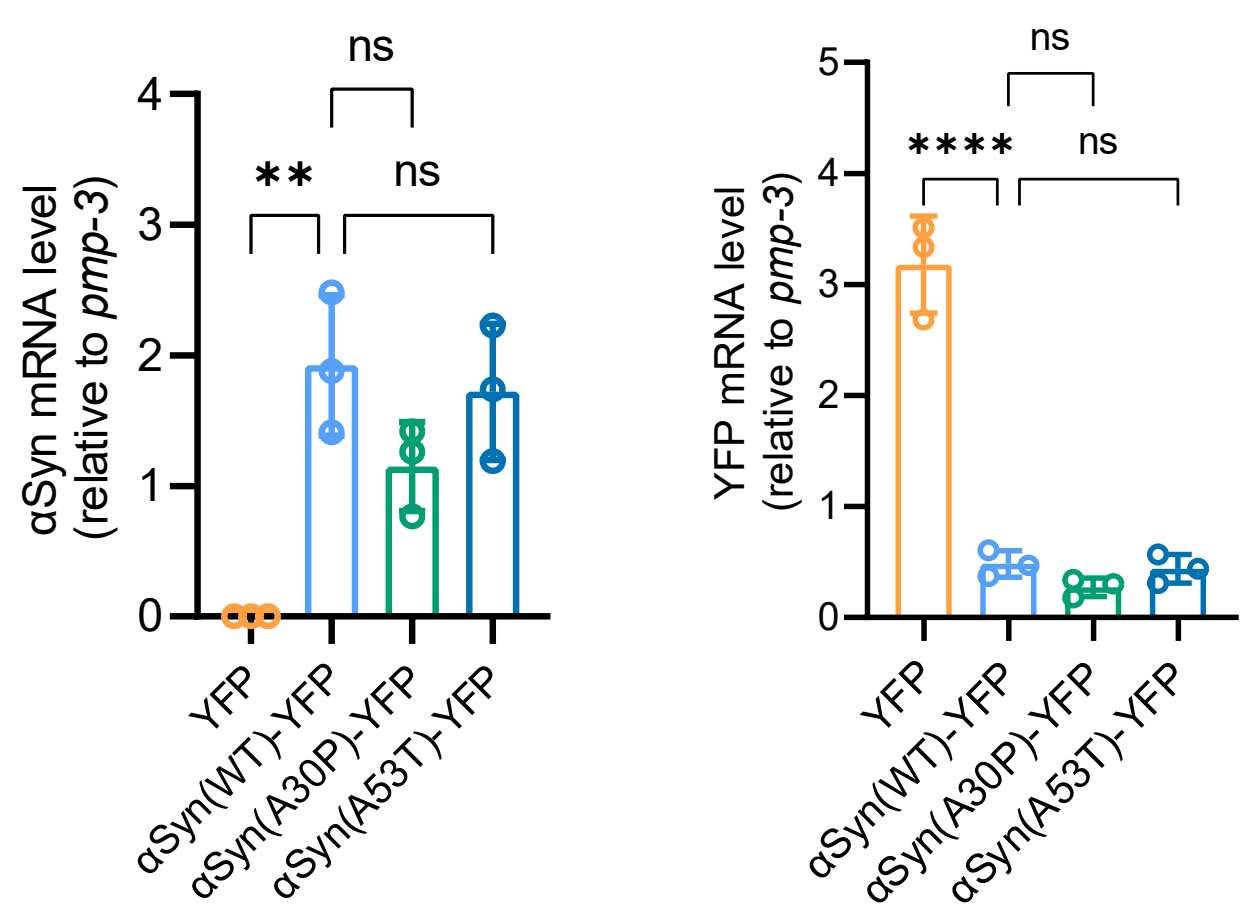

Fig. S1. (C) mRNA levels of αSyn-YFP were not significantly different among all the αSyn strains using αSyn-specific primers. **(D)** The αSyn-YFP mRNA levels were not significantly different among all the αSyn strains using YFP-specific primers.

Second, regarding αSyn aggregation, we agree that quantitative evaluation provides stronger support than qualitative fluorescence images alone. In our previous study (Perni et al., 2021, Frontiers in Cell and Developmental Biology, doi.org/10.3389/fcell.2021.552549), we quantitatively characterized aggregation behaviour in these αSyn-YFP models and demonstrated that αSyn(A53T)-YFP exhibits the strongest and earliest inclusion formation, αSyn(WT)-YFP displays an intermediate aggregation phenotype. αSyn(A30P)-YFP shows a markedly reduced and delayed inclusion formation. Inclusions are nearly absent still at day 5 of adulthood, the time at which we performed the experiments in our current manuscript and only start to increase at late adult stage. This previously established aggregation hierarchy is consistent with our current observation that the severity of TAG depletion correlates with the aggregation propensity of the different αSyn variants. We have now added this reference and clarified this point in the revised manuscript. (Revised in line 1-4, page 6).

For the neuronal αSyn expression model, we agree that quantitative confirmation of αSyn expression and aggregation would strengthen the interpretation. However, despite repeated attempts, we were unable to obtain a reliable αSyn-specific signal in the neuronal model. This limitation is likely due to the low abundance of αSyn expression in neurons and the lack of a fluorescent tag in this construct, which prevented accurate quantification of αSyn protein abundance or aggregation status under our experimental conditions. We have moved the neuronal expression data to the supplemental figures in the revised manuscript (Fig. S1E). We believe that the remaining analyses, particularly the

quantitative comparison of αSyn(WT), αSyn(A30P), and αSyn(A53T) YFP-tagged strains, provide sufficient evidence to support our conclusions regarding the relationship between αSyn inclusion-associated lipid remodeling and cellular dysfunction. (Revised in line 8, page 6).

Major comment 3:

Regarding the rescue by MCT feeding in Figure 6, there is a significant contradiction between the data and the proposed "energy deficit" model. The authors state in the Discussion that MCTs may "support mitochondrial ATP production." However, Figure 6f shows that MCT feeding reduces basal mitochondrial respiration in aSyn-YFP worms compared to untreated aSyn-YFP worms. If the primary driver of toxicity is an energy deficit, one would expect a rescue treatment to increase respiration and ATP turnover, not suppress it further. Notably, the respiratory profile presented in Figure 6f appears to show only FCCP and sodium azide injections, suggesting that oligomycin may not have been used. If oligomycin was indeed omitted, it is technically impossible to distinguish "ATP-linked respiration" from "proton leak." To support the conclusion that MCTs restore energy homeostasis despite lower respiration, the authors must measure total ATP levels. This is essential to determine whether MCT feeding maintains ATP levels via improved efficiency or if the rescue mechanism is entirely non-metabolic.

We thank the reviewer for this important observation, which prompted us to test the energetic consequences of MCT feeding. We confirm that the original OCR protocol did not include an oligomycin injection, which we now acknowledge as a limitation; distinguishing ATP-linked respiration from proton leak in that dataset is indeed therefore not possible. Unfortunately, *C. elegans* cannot take up oligomycin. We revised the text accordingly.

To further investigate the metabolic basis of MCT-mediated protection, we first expanded our supplementation experiments by testing a broader range of MCT and LCT concentrations (0.05%–2.00%) as requested by referee #1. MCT supplementation resulted in a concentration-dependent improvement in αSyn-YFP-associated motility defects, whereas LCT supplementation showed substantially weaker or no comparable rescue effects across the same concentration range (revised new Fig. 7A). These results indicate that the protective effect of MCT is unlikely to be explained simply by increased caloric availability or nonspecific lipid supplementation, but rather reflects the distinct metabolic properties of medium-chain fatty acids. (Revised in line 9-19, page 10).

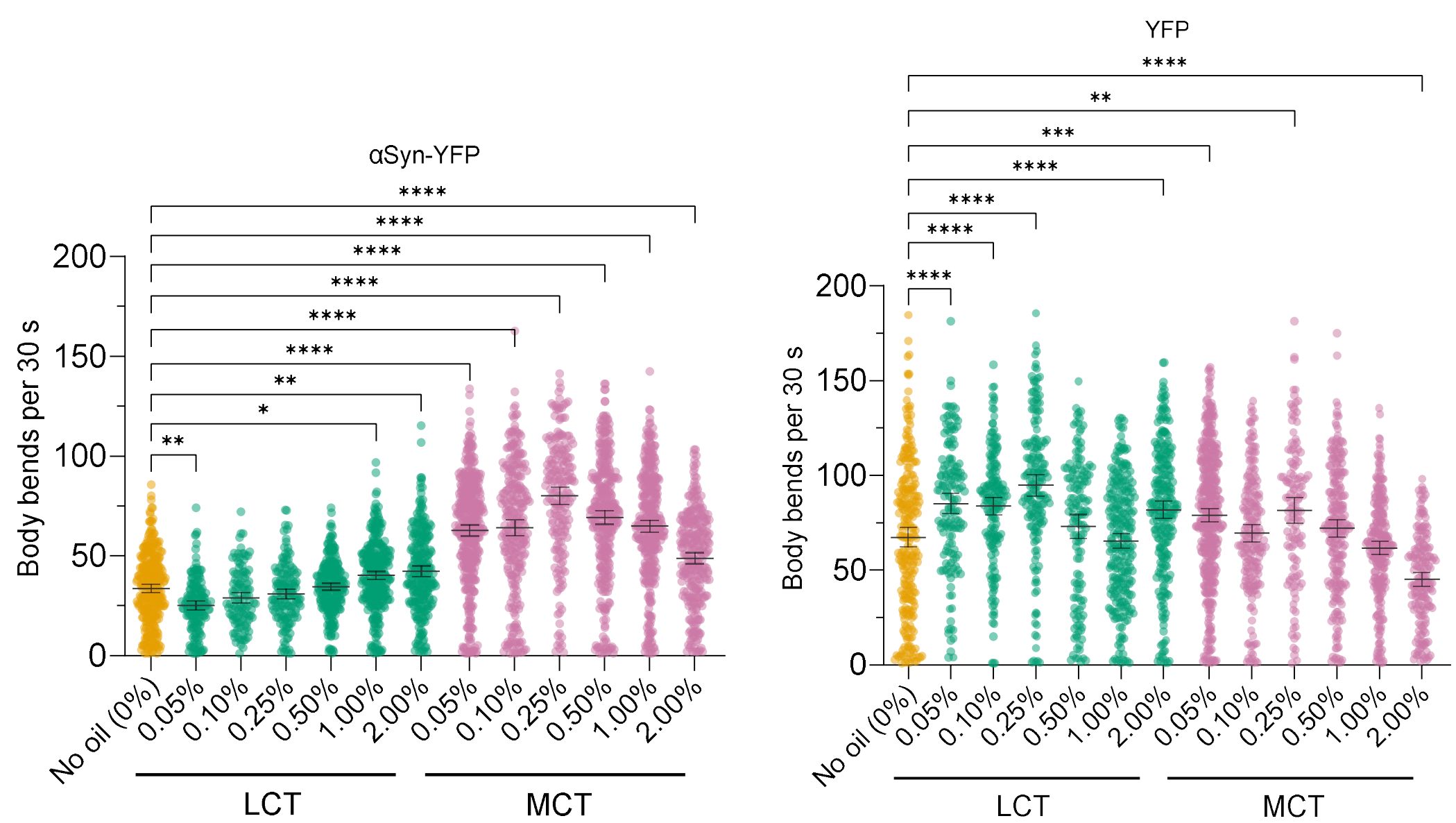

Fig. 7A. MCT supplementation improved the motility defect of αSyn-YFP worm in a dose-responsive manner, with maximal rescue observed at intermediate-to-high concentrations (*P<0.05, **P<0.01, ****P<0.0001, one-way ANOVA).

To directly determine whether MCT-mediated rescue involves restoration of cellular energy status, we measured total ATP levels following MCT and LCT supplementation Unexpectedly, both MCT and LCT, supplementation did not increase total ATP levels. Instead, increasing MCT concentrations resulted in reduced ATP levels in both αSyn-YFP and YFP control worms (Fig. R2). Importantly, this reduction occurred concomitantly with improved motility in αSyn-YFP animals. Therefore, some caution should be taken in the interpretation of these results. As stated in our response to comment #1, total ATP levels represent the balance between ATP production, consumption, and cellular energy demands; therefore, a reduced ATP pool does not necessarily indicate impaired mitochondrial function or reduced metabolic capacity. Our findings indicate that total ATP concentration cannot be used as a measure of αSyn-associated toxicity or mitochondrial function in *C. elegans* worms exposed to metabolic and motor-improving interventions. We therefore remove this suggestion from our manuscript. Our data do suggest that MCT alters metabolic substrate utilization and improves cellular resistance to αSyn-induced metabolic stress. (Revised in line 19-30, page 10).

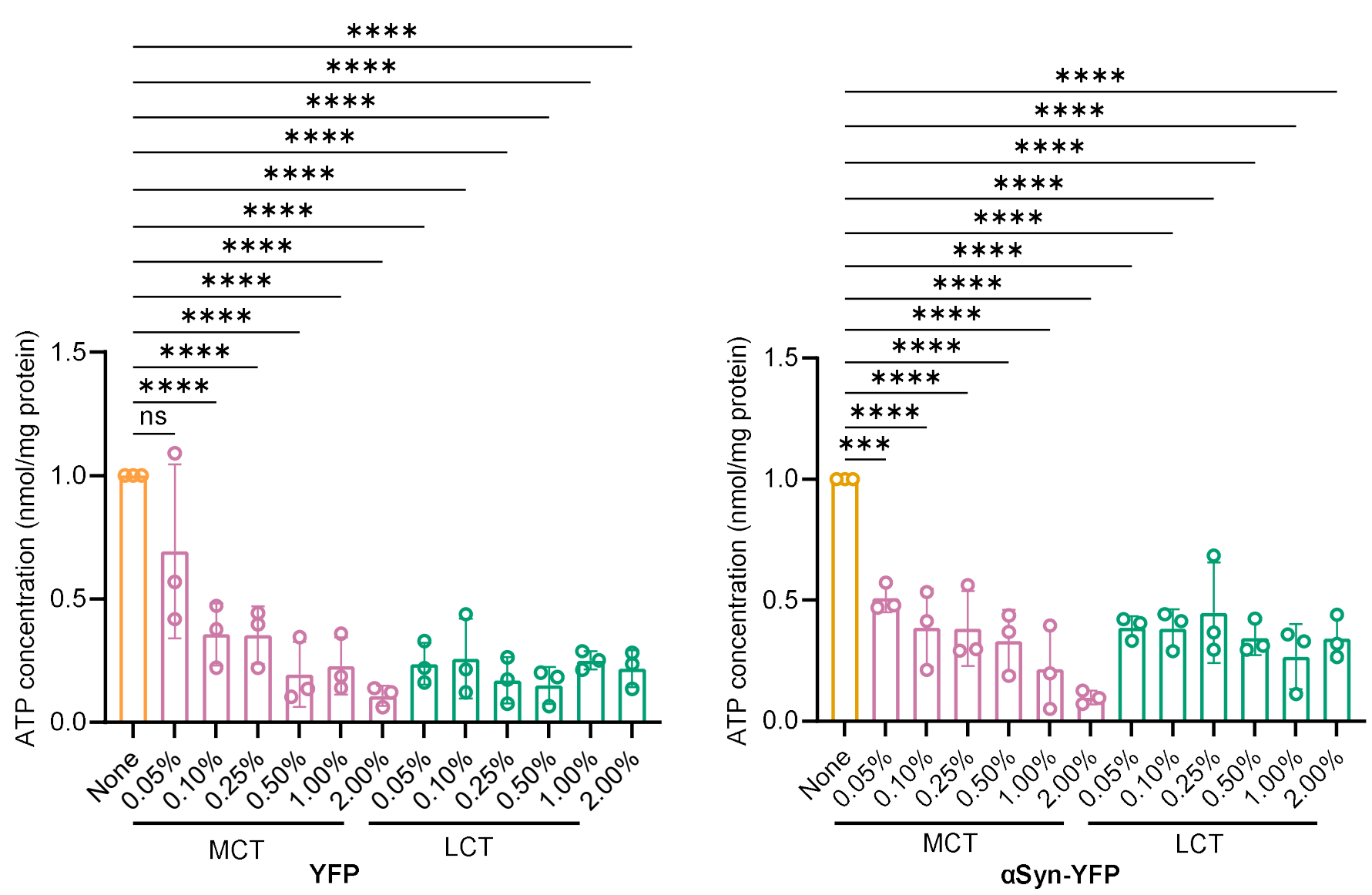

Fig. R2. MCT and LCT supplementation reduce ATP abundance, with increasing MCT concentrations resulting in a progressive reduction in ATP abundance in both αSyn-YFP and YFP worms (***P<0.001, ****P<0.0001, one-way ANOVA).

Together, these results led us to revise our mechanistic model (also visualized in combined Fig. 6A (upper panel) and 7D). Given that medium-chain fatty acids are metabolized differently from long-chain fatty acids, our results suggest that MCT's bypass specific metabolic constraints associated with long-chain lipid utilization and promote a more adaptive metabolic state under αSyn-induced lipid dysregulation. We have revised the discussion accordingly and replaced the previous "energy deficit"

model with a broader model centered on metabolic flexibility and MCT-dependent mitochondrial response. (Revised in line 12-18, page 11).

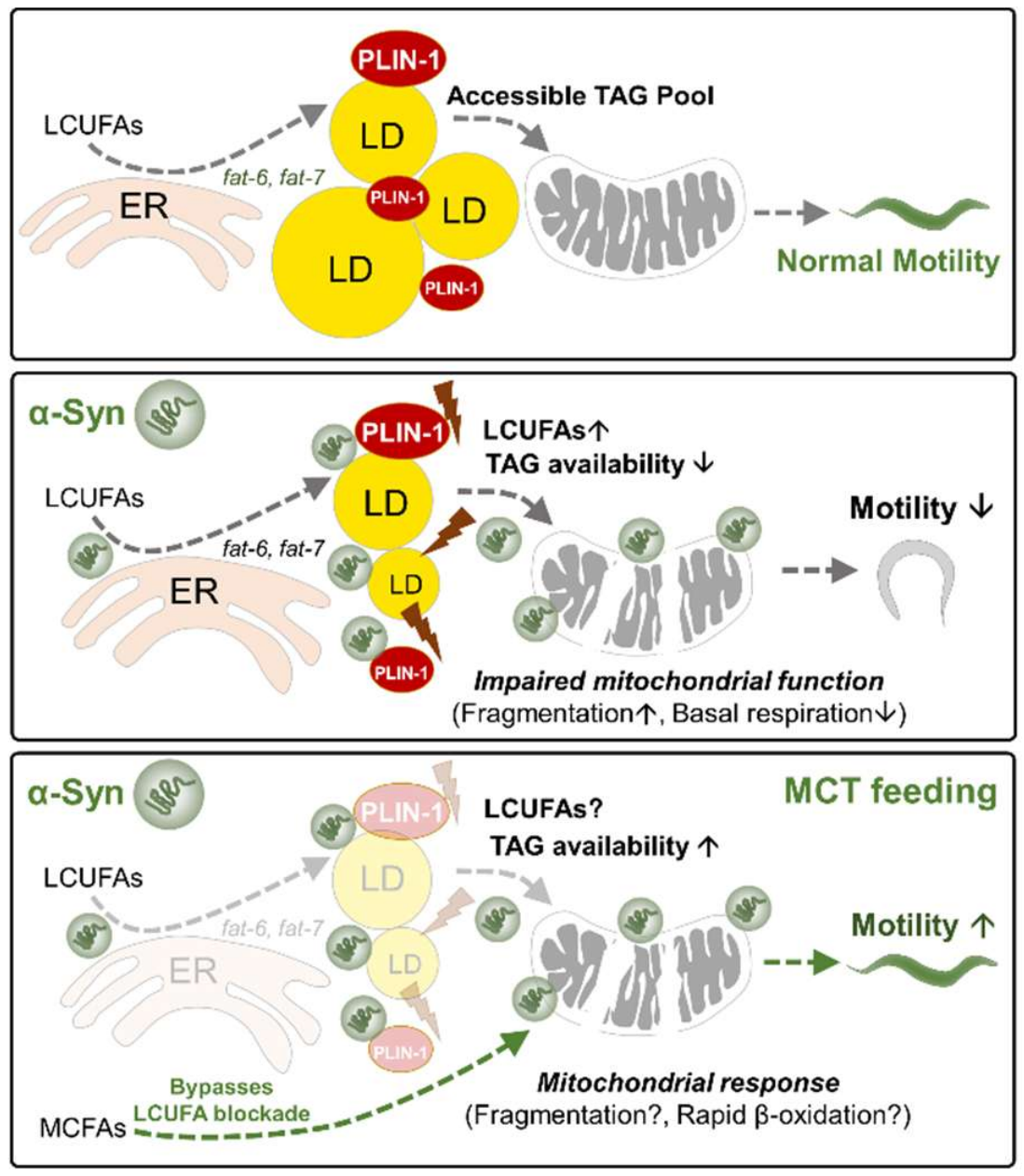

Fig. 6A (upper panel) and 7D. Proposed model illustrating that MCT supplementation bypasses LCUFA-dependent lipid metabolism, thereby promoting metabolic adaptation and improving motility. In the absence of MCT supplementation, αSyn-YFP worms exhibit mitochondrial dysfunction characterized by increased mitochondrial fragmentation.

Major comment 4:

Throughout the manuscript (Figures 1, 2, and 4), Oil Red O (ORO) intensity is used to quantify fat content. However, the text notes that Syn expression and fat-6 RNAi significantly reduce worm size. Consequently, a reduction in total ORO intensity might simply reflect the smaller body volume rather than a metabolic defect. If such normalization has not been performed, the ORO intensity data should be normalized by body volume or size to ensure the phenotype represents a true reduction in lipid density.

We thank the reviewer for raising this important technical point. We confirm that all ORO quantifications in Figures 1, 2, and 4 were normalized to the longitudinal section area corresponding to the anterior intestine, ensuring that the reported values reflect lipid content normalized by animal size. This normalization was applied in the original analysis but was not explicitly described in the Methods. We have now added the following description:

"For Oil Red O quantification, worms were imaged under identical exposure settings. The anterior intestinal region was selected for analysis for each worm. Using ImageJ, the longitudinal-sectional area of the intestine was measured from brightfield images, and the mean staining intensity within this region was quantified from corresponding ORO images. Final ORO intensity values are reported as mean staining intensity normalized to intestinal area (pixels^2), representing lipid density." (Revised in line 7-12, page 17).

Major comment 5:

In Figure 3c, the authors report Perilipin (PLIN-1) mislocalization. However, it is unclear from the current data whether PLIN-1 is dissociating from existing LDs, or if the LDs themselves are absent (a

possibility supported by the author's note in the Discussion regarding "reduction in LD surface area"). To distinguish between a "reduction in LD number" and a "loss of PLIN-1 coating on intact LDs," the authors should perform co-staining with a neutral lipid dye alongside the PLIN-1 marker.

We thank the reviewer for raising this important point. We agree that distinguishing between a reduction in LD structures and dissociation of PLIN-1 from otherwise intact LDs is essential for interpreting the PLIN-1 phenotype. Therefore, we have revised our interpretation and no longer describe the phenotype as direct PLIN-1 dissociation from intact LDs. Instead, our data support a model in which αSyn-associated lipid dysregulation alters LD organization, leading to altered PLIN-1 distribution.

Consistent with this interpretation, lipidomic and imaging analyses demonstrate reduced TAG levels and altered LD-associated structures in αSyn-expressing animals. We have revised the manuscript accordingly and clarified that PLIN-1 redistribution serves as a marker of altered LD organization rather than direct evidence of PLIN-1 dissociation from intact LDs or physical displacement by αSyn inclusions. (Revised in line 12-17, page 13).

Major comment 6:

In Figure 5e, the authors show motility rescue in the membrane-binding deficient mutant (aSyn-delta). It is essential to characterize the bioenergetic status of this mutant. Specifically, the authors should verify whether this mutant prevents mitochondrial fragmentation and, crucially, whether it restores ATP levels to baseline. Confirming that the loss of membrane binding prevents the energy deficit is necessary to substantiate the claim that membrane interaction is the upstream cause of the bioenergetic failure.

We thank the reviewer for this important suggestion. We agree that characterization of mitochondrial morphology in the membrane-binding deficient αSyn mutant is critical for determining whether membrane interaction contributes to αSyn-associated mitochondrial defects.

To address this point, we performed additional mitochondrial morphology analyses using TOMM-20::mRFP as a mitochondrial outer membrane marker in αSyn-YFP and αSyn(Δ)-YFP worms at day 5 of adulthood. Consistent with our previous observations, αSyn-YFP expression resulted in pronounced mitochondrial morphological abnormalities, characterized by increased mitochondrial fragmentation, reduced mitochondrial aspect ratio, and decreased mitochondrial area compared with YFP control animals (new Fig. 6D,E,F; new Fig. S7). In contrast, αSyn(Δ)-YFP worms, which are defective in membrane binding, exhibited largely preserved mitochondrial morphology, with no significant reduction in mitochondrial aspect ratio or mitochondrial area compared with YFP controls. These results indicate that disruption of αSyn membrane interaction prevents the mitochondrial fragmentation phenotype associated with αSyn expression. (Revised in line 30-32, page 9).

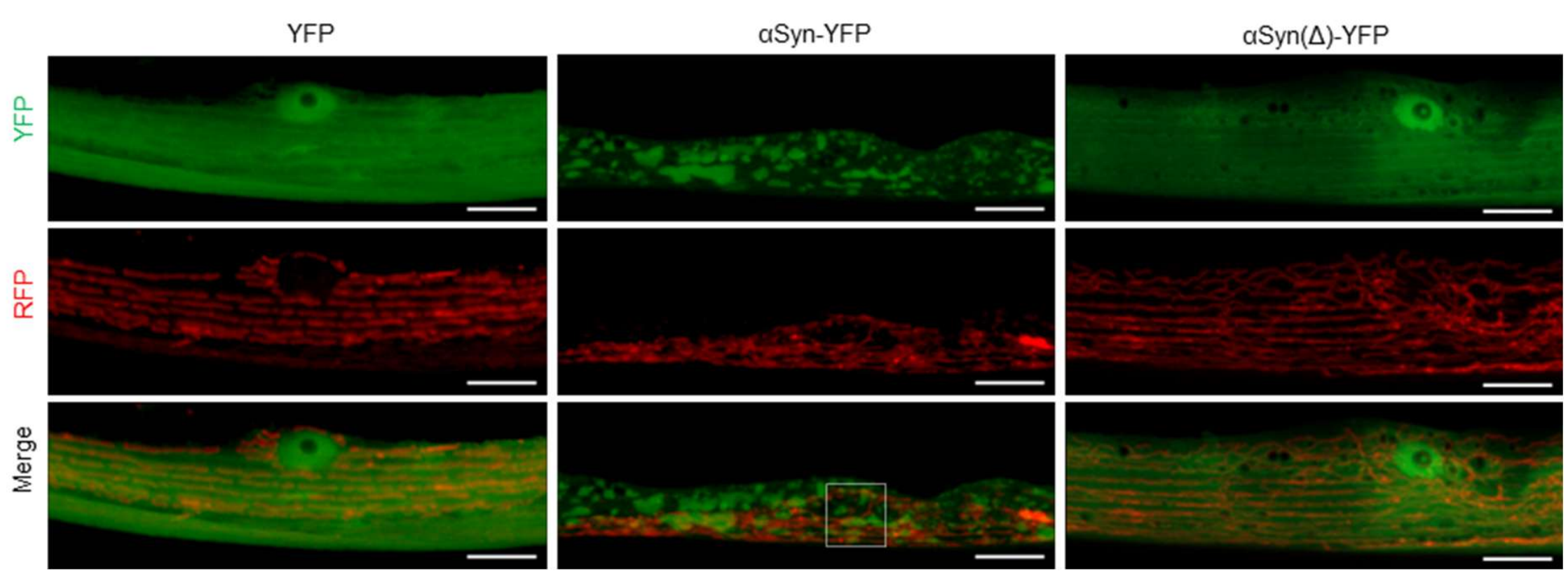

Fig. 6D. Representative TOMM-20::mRFP images reveal increased mitochondrial fragmentation in αSyn(WT)-YFP worms, whereas this phenotype is not apparent in αSyn(Δ)-YFP worms expressing a lipid-binding-deficient αSyn variant. Scale bar, 10 µm.

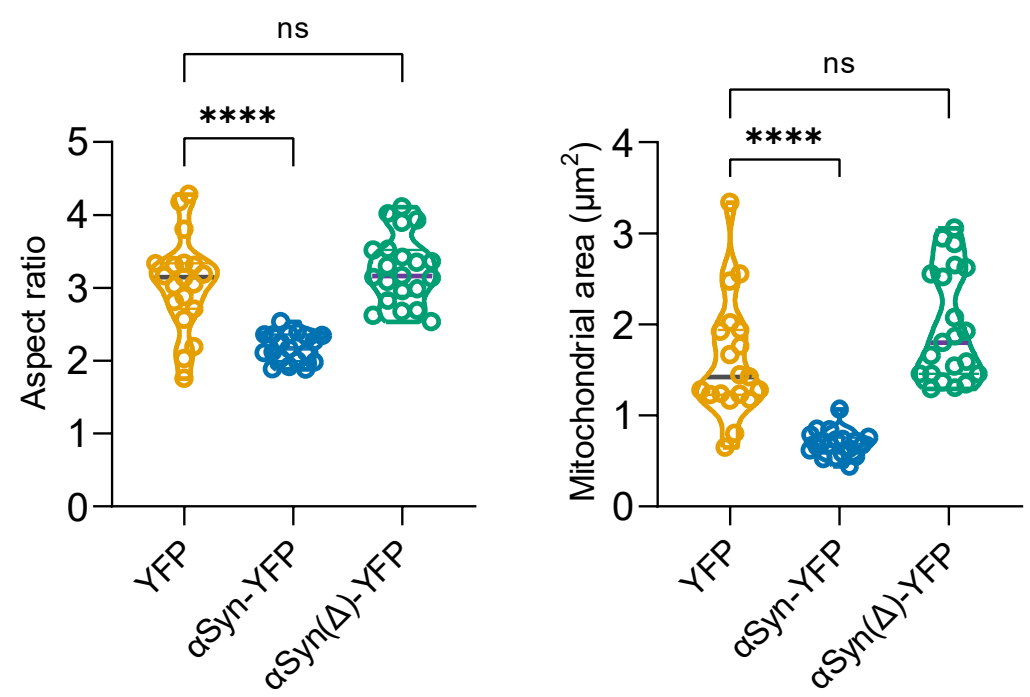

Fig. 6E. Quantification of mitochondrial morphology shows reduced mitochondrial aspect ratio and area in αSyn(WT)-YFP worms compared with YFP and αSyn(Δ)-YFP worms at day 5 of adulthood (n ≥ 19 worms per condition).

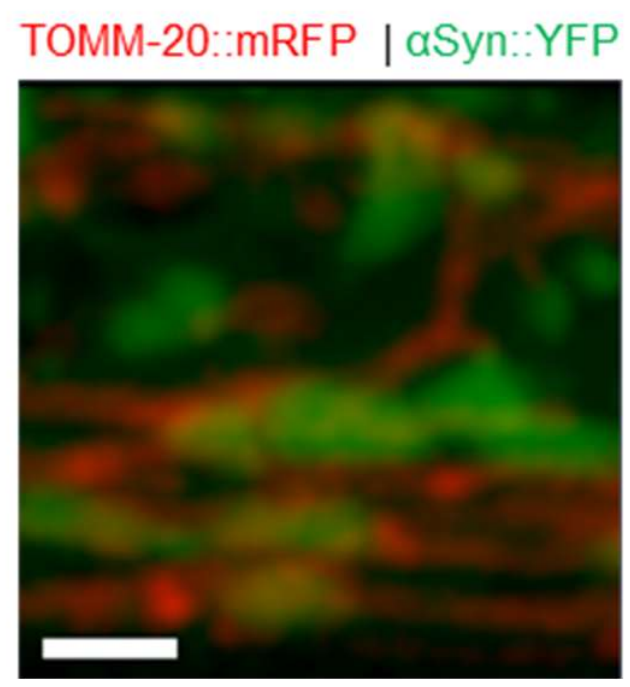

Fig. 6F. Representative image showing spatial colocalization of αSyn inclusions with mitochondria in αSyn-YFP worms. Scale bar, 2 μm.

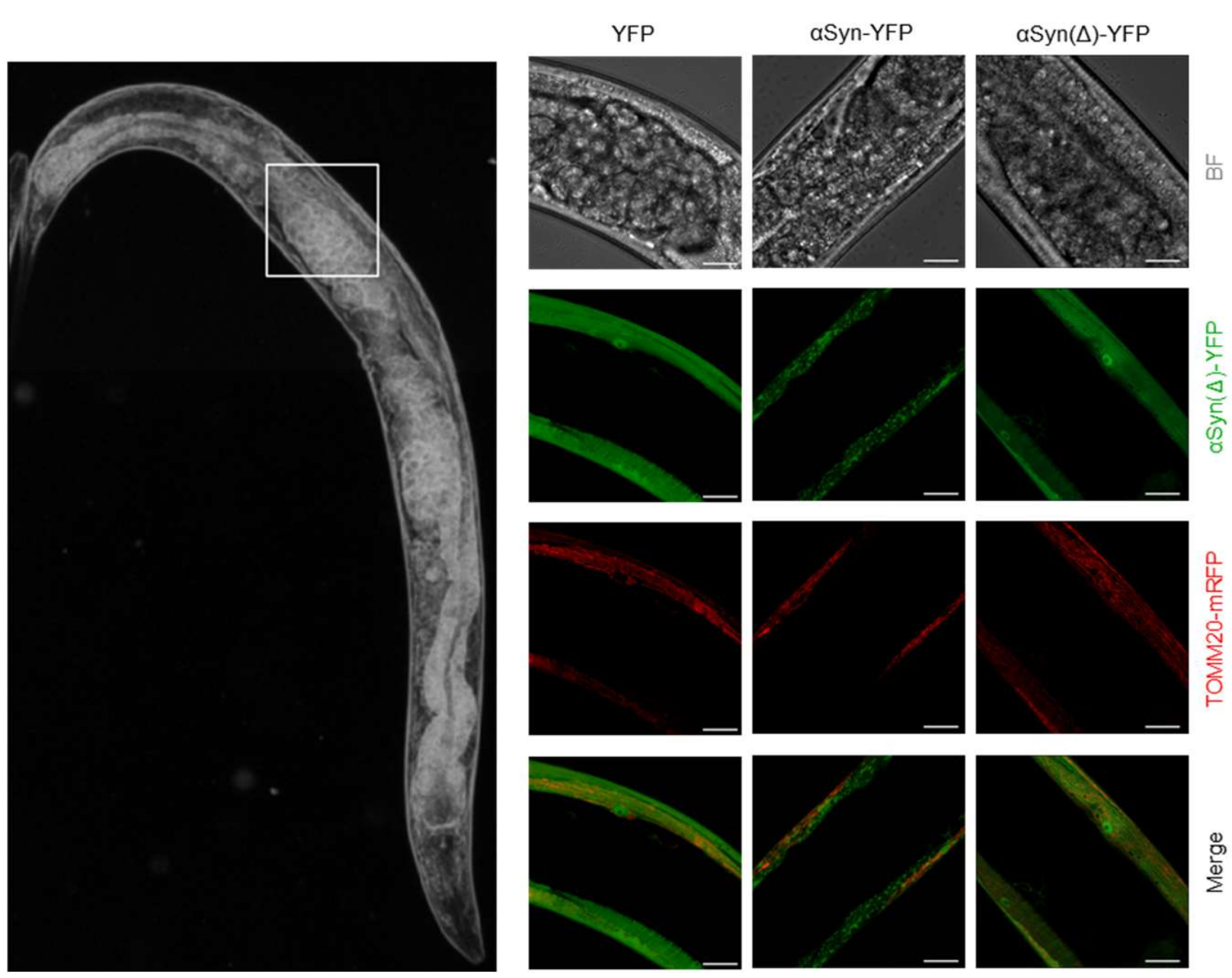

Fig. S7. αSyn(WT)-YFP expression but not αSyn(Δ)-YFP expression is associated with increased mitochondrial fragmentation. Scale bar: 20 μm.

Together with the observation that αSyn(Δ)-YFP rescues αSyn-associated motility defects (Fig. 5F), these findings support the conclusion that membrane interaction is an important upstream event

contributing to αSyn-induced mitochondrial dysfunction. Specifically, the inability of αSyn(Δ)-YFP to induce mitochondrial fragmentation suggests that membrane association is required for initiating downstream mitochondrial abnormalities.

We have incorporated these new data into the revised manuscript and have revised the discussion to clarify that membrane binding contributes to αSyn-associated mitochondrial defects, while avoiding the overstatement that membrane interaction alone fully determines bioenergetic failure. (Revised in line 1-4, page 10).

To address the ATP levels, we performed additional ATP measurements in animals expressing different αSyn variants. Interestingly, αSyn variants with intact membrane-binding capacity, including αSyn(WT)-YFP and αSyn(A53T)-YFP, did not significantly alter ATP levels compared with YFP controls. In contrast, αSyn(Δ)-YFP animals exhibited a significant increase in ATP levels relative to YFP controls (new Fig. S8C).

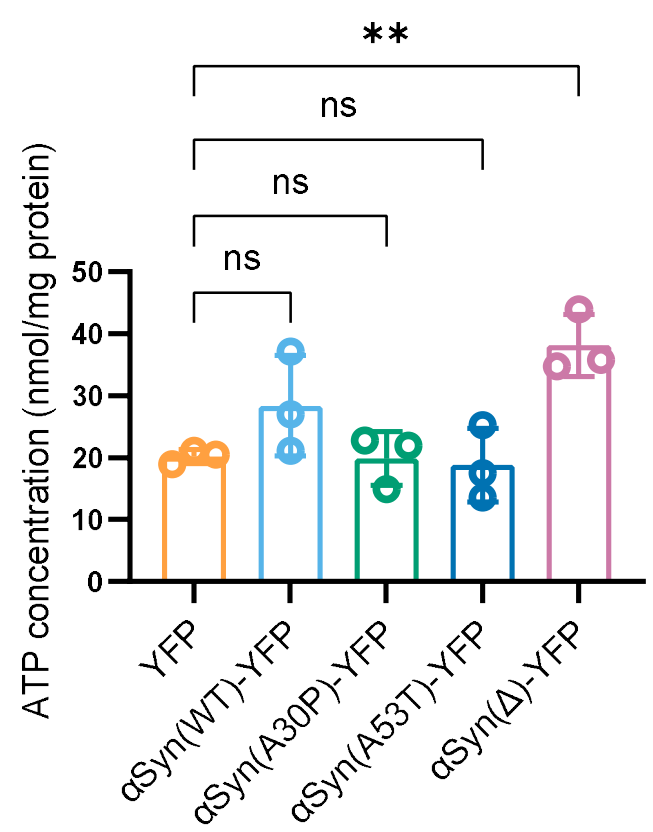

Fig. S8C. αSyn(Δ)-YFP animals show significantly increased ATP abundance relative to YFP controls (**P<0.01, one-way ANOVA).

Unlike membrane-binding competent αSyn variants, αSyn(Δ)-YFP animals exhibited increased ATP levels, suggesting that preventing membrane association permits a distinct metabolic adaptation associated with preserved cellular function. Importantly, the increased ATP level in αSyn(Δ)-YFP animals is consistent with our observation that this mutant does not induce the same degree of functional toxicity as membrane-binding competent αSyn variants.

However, we interpret these ATP changes cautiously, as explained above under previous comments. The absence of ATP reduction in membrane-binding competent αSyn strains indicates that steady-state ATP abundance is not a universal readout of αSyn toxicity. Thus, the protective effect of αSyn(Δ)-YFP is unlikely to be explained solely by ATP restoration, but instead reflects prevention of upstream membrane-associated disturbances that affect lipid homeostasis, organelle organization, or mitochondrial function. (Revised in line 3-12, page 11).

In sum, we believe these revisions have substantially strengthened the manuscript by addressing mechanistic gaps, resolving apparent contradictions, and providing more quantitative rigor. We are grateful to the reviewers for their time and expertise, which have helped us present a more accurate and compelling story. We hope that the revised manuscript is now suitable for publication in EMBO Report.

Sincerely,

Ellen Nollen, Tao Zhang,

On behalf of all co-authors.

e.a.a.nollen@umcg.nl; t.zhang@mail.hzau.edu.cn

References:

Girard, V. *et al.* Abnormal accumulation of lipid droplets in neurons induces the conversion of alpha-Synuclein to proteolytic resistant forms in a Drosophila model of Parkinson's disease. *PLoS Genet.* 17, e1009921 (2021).

Perni, M. *et al*. Comparative Studies in the A30P and A53T α-Synuclein C. elegans Strains to Investigate the Molecular Origins of Parkinson's Disease. *Front. Cell Dev. Biol*. **9**, (2021).

Roux, A. E. *et al*. Individual cell types in C. elegans age differently and activate distinct cell-protective responses. *Cell Rep*. **42**, (2023).

1st Revision - Editorial Decision 31st Aug 2026

Dear Ellen,

Thank you for the submission of your revised manuscript. We have now received the enclosed reports from the referees that were asked to assess it and I am happy to say that both support its publication now. Only a few editorial requests will need to be addressed before we can proceed with the official acceptance of your manuscript:

- Both the main and suppl. figures need to be uploaded as individual, production quality figure files without their legends. Please add the figure legends to the ms file.

- Please delete this sentence from the Data Availabilty Statement: "All materials generated in this study will be shared upon request."

- Please correct the conflict of interest subheading to "Disclosure and Competing Interests Statement"

- There are some author name discrepancies: Lale Gürgöndü in the ms vs. Lale Güngördü in our online system; Anna Ainslie Castellanos in the ms vs. Anna P Ainslie in our system; M. Rebecca Heiner Fokkema in the ms vs. M. Rebecca Fokkema in our system, please correct.

- The author credits need to be removed from the ms file. All credits need to be entered during online ms submission.

- The FUNDING INFO National Natural Science Foundation of China (Grant No. 32402082) is missing in our online system; the funders provided in the Comments box need to be removed and provided as separate entries via the More Funders option.

- A callout is missing for Fig. 6E, please add. Callouts for the suppl. figures need correction as "supplementary" should not be used. Table S1 needs to be corrected to Table EV1 in all places. There are 9 suppl. figures, all of them can be EV figures and their names need to be corrected in all places. The legends of the EV figures need to be in the main ms file after the main figure legends. Alternatively, you can have one Appendix pdf file for all supplementary figures. Please see our guide to authors for more information.

- The Reagents & Tools TABLE needs to be removed from the ms file and uploaded separately.

- The Source Data for main and EV figures need to be uploaded separately, i.e. one SD folder per main and per EV figure. The SD for the Appendix figures (if there will be an Appendix) should be combined into one zip folder.

- The manuscript sections should be in the following order: Title page - Abstract - Introduction - Results - Discussion - Methods - Data Availability - Acknowledgments - Disclosure Statement & Competing Interests - References - Figure Legends - (Main Tables with legends if applicable) - Expanded View Figure Legends.

* Figure Legends - Comments *

- Please note that the exact p values are not provided in the legends of figures 1D-G; 2D, 3D, 4B, C; 5C, D, E, F; 6 C, E; 7A, B. Please provide exact values as reasonable.
- Please indicate the statistical test used for data analysis in the legend of figure 2A
- Please note that the box plots need to be defined in terms of minima, maxima, centre, bounds of box and whiskers, and percentile in the legends of figures 1D-G; 2C, D.
- Please note that the error bars are not defined in the legends of figures 2A, 3D, 4B, C; 5C, D, E, F; 6B, C; 7A, B, C, E.

I would like to suggest a minor change to the title. Do you agree with:

Condensate-Driven Triglyceride Reduction Links α-Synuclein to Mitochondrial Dysfunction

And do you agree with these changes to these 2 sentences in the abstract:

Through lipidomic profiling of Caenorhabditis elegans, we find that αSyn progressively alters lipid metabolism in aging worms. αSyn reduces the overall content of triacylglycerols (TAG) and disrupts the structure of lipid droplets (LD) and mitochondria.

EMBO press papers are accompanied online by A) a short (1-2 sentences) summary of the findings and their significance, B) 2-3 bullet points highlighting key results and C) a synopsis image that is exactly 550 pixels wide and 300 pixels high. The synopsis

image should provide a sketch of the major findings, like a graphical abstract. Please note that text needs to be readable at the final size. Please send us this information along with the final manuscript.

I look forward to seeing a final version of your manuscript as soon as possible.

Best wishes,
Esther

Esther Schnapp, PhD
Senior Editor
EMBO reports

Referee #1:

My concerns have been addressed.

Referee #3:

The authors have successfully addressed all of my concerns raised in the previous review. By performing the requested experiments and carefully revising the mechanistic model, they have resolved the logical inconsistencies present in the original manuscript.

All minor editorial requests have been addressed by the authors.

Prof. Ellen Nollen
UMC Groningen
European Research Institute for the Biology of Ageing (Eriba)
PO Box 196
Groningen 9700AD
Netherlands

Dear Prof. Nollen,

I am very pleased to accept your manuscript for publication in the next available issue of EMBO reports. Thank you for your contribution to our journal.

Your manuscript will be processed for publication by EMBO Press. It will be copy edited and you will receive page proofs prior to publication. Please note that you will be contacted by Springer Nature Author Services to complete licensing and payment information.

You may qualify for financial assistance for your publication charges - either via a Springer Nature fully open access agreement or an EMBO initiative. Check your eligibility: https://link.springer.com/journal/44319/how-to-publish-with-us

Should you be planning a Press Release on your article, please notify the production team via this form in order to coordinate publication and release dates: https://forms.cloud.microsoft/e/eH4A8JVP6g

If you have any questions, please do not hesitate to contact the Editorial Office. Thank you for your contribution to EMBO Reports.

Best regards,
Esther

Esther Schnapp, PhD
Senior Editor
EMBO reports

>>> Please note that it is EMBO Reports policy for the transcript of the editorial process (containing referee reports and your response letter) to be published as an online supplement to each paper. If you do NOT want this, you will need to inform the Editorial Office via email immediately. More information is available here: https://link.springer.com/partners/embo-press/editorial-policies#Peer%20review

EMBO Press Author Checklist

Corresponding Author Name: Ellen A. A. Nollen, Tao Zhang	
Journal Submitted to: EMBO Reports	
Manuscript Number: EMBOR-2025-63388V1	

USEFUL LINKS FOR COMPLETING THIS FORM
The EMBO Journal - Author Guidelines
EMBO Reports - Author Guidelines
Molecular Systems Biology - Author Guidelines
EMBO Molecular Medicine - Author Guidelines

Reporting Checklist for Life Science Articles (updated January
This checklist is adapted from Materials Design Analysis Reporting (MDAR) Checklist for Authors. MDAR establishes a minimum set of requirements in transparent reporting in the life sciences (see Statement of Task: 10.31222/osf.io/9sm4x). Please follow the journal's guidelines in preparing your
Please note that a copy of this checklist will be published alongside your article.

Abridged guidelines for figures
1. Data
The data shown in figures should satisfy the following conditions:
- the data were obtained and processed according to the field's best practice and are presented to reflect the results of the experiments in an accurate and unbiased manner.
- ideally, figure panels should include only measurements that are directly comparable to each other and obtained with the same assay.
- plots include clearly labeled error bars for independent experiments and sample sizes. Unless justified, error bars should not be shown for technical
- if n<5, the individual data points from each experiment should be plotted. Any statistical test employed should be justified.
- Source Data should be included to report the data underlying figures according to the guidelines set out in the authorship guidelines on Data

2. Captions
Each figure caption should contain the following information, for each panel where they are relevant:
- a specification of the experimental system investigated (eg cell line, species name).
- the assay(s) and method(s) used to carry out the reported observations and measurements.
- an explicit mention of the biological and chemical entity(ies) that are being measured.
- an explicit mention of the biological and chemical entity(ies) that are altered/varied/perturbed in a controlled manner.
- the exact sample size (n) for each experimental group/condition, given as a number, not a range;
- a description of the sample collection allowing the reader to understand whether the samples represent technical or biological replicates (including how many animals, litters, cultures, etc.).
- a statement of how many times the experiment shown was independently replicated in the laboratory.
- definitions of statistical methods and measures:
  - common tests, such as t-test (please specify whether paired vs. unpaired), simple χ2 tests, Wilcoxon and Mann-Whitney tests, can be unambiguously identified by name only, but more complex techniques should be described in the methods section;
  - are tests one-sided or two-sided?
  - are there adjustments for multiple comparisons?
  - exact statistical test results, e.g., P values = x but not P values < x;
  - definition of 'center values' as median or average;
  - definition of error bars as s.d. or s.e.m.

Please complete ALL of the questions below.
Select "Not Applicable" only when the requested information is not relevant for your study.

Materials

Newly Created Materials	Information included in the manuscript?	In which section is the information available? (Reagents and Tools Table, Materials and Methods, Figures, Data Availability Section)
New materials and reagents need to be available; do any restrictions apply?	Yes	Reagents and Tools Table, Materials and Methods

Antibodies	Information included in the manuscript?	In which section is the information available? (Reagents and Tools Table, Materials and Methods, Figures, Data Availability Section)
For **antibodies** provide the following information:<br>- Commercial antibodies: RRID (if possible) or supplier name, catalogue number and or/clone number<br>- Non-commercial: RRID or citation	Yes	Reagents and Tools Table

DNA and RNA sequences	Information included in the manuscript?	In which section is the information available? (Reagents and Tools Table, Materials and Methods, Figures, Data Availability Section)
**Short novel DNA or RNA including primers, probes**: provide the sequences.	Yes	Reagents and Tools Table

Cell materials	Information included in the manuscript?	In which section is the information available? (Reagents and Tools Table, Materials and Methods, Figures, Data Availability Section)
**Cell lines:** Provide species information, strain. Provide accession number in repository **OR** supplier name, catalog number, clone number, and/**OR** RRID.	Not Applicable	
**Primary cultures:** Provide species, strain, sex of origin, genetic modification status.	Not Applicable	
Report if the cell lines were recently **authenticated** (e.g., by STR profiling) and tested for mycoplasma contamination.	Not Applicable	

Experimental animals	Information included in the manuscript?	In which section is the information available? (Reagents and Tools Table, Materials and Methods, Figures, Data Availability Section)
**Laboratory animals or Model organisms:** Provide species, strain, sex, age, genetic modification status. Provide accession number in repository **OR** supplier name, catalog number, clone number, **OR** RRID.	Yes	Reagents and Tools Table
**Animal observed in or captured from the field:** Provide species, sex, and age where possible.	Not Applicable	
Please detail **housing and husbandry conditions**.	Not Applicable	

Plants and microbes	Information included in the manuscript?	In which section is the information available? (Reagents and Tools Table, Materials and Methods, Figures, Data Availability Section)
**Plants:** provide species and strain, ecotype and cultivar where relevant, unique accession number if available, and source (including location for collected wild specimens).	Not Applicable	
**Microbes:** provide species and strain, unique accession number if available, and source.	Not Applicable	

Human research participants	Information included in the manuscript?	In which section is the information available? (Reagents and Tools Table, Materials and Methods, Figures, Data Availability Section)
If collected and within the bounds of privacy constraints report on age, sex and gender or ethnicity for all study participants.	Not Applicable	

Core facilities	Information included in the manuscript?	In which section is the information available? (Reagents and Tools Table, Materials and Methods, Figures, Data Availability Section)
If your work benefited from core facilities, was their service mentioned in the acknowledgments section?	Yes	Materials and Methods

Design

Study protocol	Information included in the manuscript?	In which section is the information available? (Reagents and Tools Table, Materials and Methods, Figures, Data Availability Section)
If study protocol has been **pre-registered, provide DOI in the manuscript**. For clinical trials, provide the trial registration number **OR** cite DOI.	Not Applicable	
Report the **clinical trial registration number** (at ClinicalTrials.gov or equivalent), where applicable.	Not Applicable	

Laboratory protocol	Information included in the manuscript?	In which section is the information available? (Reagents and Tools Table, Materials and Methods, Figures, Data Availability Section)
Provide DOI OR other citation details if **external detailed step-by-step protocols** are available.	Yes	References

Experimental study design and statistics	Information included in the manuscript?	In which section is the information available? (Reagents and Tools Table, Materials and Methods, Figures, Data Availability Section)
Include a statement about **sample size** estimate even if no statistical methods were used.	Yes	Figures
Were any steps taken to minimize the effects of subjective bias when allocating animals/samples to treatment (e.g. **randomization procedure**)? If yes, have they been described?	Yes	Materials and Methods
Include a statement about **blinding** even if no blinding was done.	Not Applicable	
Describe **inclusion/exclusion criteria** if samples or animals were excluded from the analysis. Were the criteria pre-established?<br><br>If sample or data points were omitted from analysis, report if this was due to attrition or intentional exclusion and provide justification.	Not Applicable	
For every figure, are **statistical tests** justified as appropriate? Do the data meet the assumptions of the tests (e.g., normal distribution)? Describe any methods used to assess it. Is there an estimate of variation within each group of data? Is the variance similar between the groups that are being statistically compared?	Yes	Materials and Methods

Sample definition and in-laboratory replication	Information included in the manuscript?	In which section is the information available? (Reagents and Tools Table, Materials and Methods, Figures, Data Availability Section)
In the figure legends: state number of times the experiment was **replicated** in laboratory.	Yes	Figures
In the figure legends: define whether data describe **technical or biological replicates**.	Yes	Figures

Ethics

Ethics	Information included in the manuscript?	In which section is the information available? (Reagents and Tools Table, Materials and Methods, Figures, Data Availability Section)
Studies involving **human participants**: State details of **authority granting ethics approval** (IRB or equivalent committee(s), provide reference number for approval.	Not Applicable	
Studies involving **human participants**: Include a statement confirming that **informed consent** was obtained from all subjects and that the experiments conformed to the principles set out in the WMA Declaration of Helsinki and the Department of Health and Human Services Belmont Report.	Not Applicable	
Studies involving **human participants:** For publication of **patient photos**, include a statement confirming that consent to publish was obtained.	Not Applicable	
Studies involving experimental **animals**: State details of **authority granting ethics approval** (IRB or equivalent committee(s), provide reference number for approval. Include a statement of compliance with ethical regulations.	Not Applicable	
Studies involving **specimen and field samples:** State if relevant **permits** obtained, provide details of authority approving study; if none were required, explain why.	Not Applicable	

Dual Use Research of Concern (DURC)	Information included in the manuscript?	In which section is the information available? (Reagents and Tools Table, Materials and Methods, Figures, Data Availability Section)
Could your study fall under dual use research restrictions? Please check biosecurity documents and list of **select agents and toxins** (CDC): https://www.selectagents.gov/sat/list.htm	Not Applicable	
If you used a select agent, is the security level of the lab appropriate and reported in the manuscript?	Not Applicable	
If a study is subject to dual use research of concern regulations, is the name of the **authority granting approval and reference number** for the regulatory approval provided in the manuscript?	Not Applicable	

Reporting

The MDAR framework recommends adoption of discipline-specific guidelines, established and endorsed through community initiatives. Journals have their own policy about requiring specific guidelines and recommendations to complement MDAR.

Adherence to community standards	Information included in the manuscript?	In which section is the information available? (Reagents and Tools Table, Materials and Methods, Figures, Data Availability Section)
State if relevant guidelines or checklists (e.g., **ICMJE, MIBBI, ARRIVE, PRISMA**) have been followed or provided.	Not Applicable	
For **tumor marker prognostic studies,** we recommend that you follow the **REMARK** reporting guidelines (see link list at top right). See author guidelines, under 'Reporting Guidelines'. Please confirm you have followed these guidelines.	Not Applicable	
For **phase II and III randomized controlled trials**, please refer to the **CONSORT** flow diagram (see link list at top right) and submit the CONSORT checklist (see link list at top right) with your submission. See author guidelines, under 'Reporting Guidelines'. Please confirm you have submitted this list.	Not Applicable	

Data Availability

Data availability	Information included in the manuscript?	In which section is the information available? (Reagents and Tools Table, Materials and Methods, Figures, Data Availability Section)
Have **primary datasets** been deposited according to the journal's guidelines (see 'Data Deposition' section) and the respective accession numbers provided in the Data Availability Section?	Yes	Data Availability Section
Were **human clinical and genomic datasets** deposited in a public access-controlled repository in accordance to ethical obligations to the patients and to the applicable consent agreement?	Not Applicable	
Are **computational models** that are central and integral to a study available without restrictions in a machine-readable form? Were the relevant accession numbers or links provided?	Not Applicable	
If publicly available data were reused, provide the respective **data citations in the reference list**.	Yes	Figures
