# Supplementary material for: Condensate-driven triglyceride reduction links α-synuclein to mitochondrial dysfunction
Source: EMBO Rep. 2026 Sep 29;27(21):6277–304. doi: 10.1038/s44319-026-00954-7 (PMC13743766; doi:10.1038/s44319-026-00954-7)
Supplement: Supplementary file 11 [file 44319_2026_954_MOESM3_ESM.zip › Source_Data_Figure_1/Source Data Fig 1G Unprocessed western blots.docx]

Unprocessed western blots Fig. 1G

Anti-αSynuclein

Anti-Tubulin
