# Supplementary material for: Condensate-driven triglyceride reduction links α-synuclein to mitochondrial dysfunction
Source: EMBO Rep. 2026 Sep 29;27(21):6277–304. doi: 10.1038/s44319-026-00954-7 (PMC13743766; doi:10.1038/s44319-026-00954-7)
Supplement: Supplementary file 12 [file 44319_2026_954_MOESM4_ESM.zip › Source_Data_Figure_2/Source Data Fig 2A Unprocessed western blots.docx]

Unprocessed western blots Fig. 2A

Anti-GFP

Anti-Tubulin
